# Copper Single‐Atom Catalyst for Efficient C─S Coupling in Thioether Synthesis

**DOI:** 10.1002/anie.202510632

**Published:** 2025-08-06

**Authors:** Theodore A. Gazis, Shilpa Palit, Luis A. Cipriano, Nicolò Allasia, Sean M. Collins, Quentin M. Ramasse, Ik Seon Kwon, Martin Sterrer, Giovanni Di Liberto, Gianvito Vilé

**Affiliations:** ^1^ Department of Chemistry, Materials, and Chemical Engineering “Giulio Natta” Politecnico di Milano Piazza Leonardo da Vinci 32 Milano 20133 Italy; ^2^ Bragg Centre for Materials Research, School of Chemical and Process Engineering & School of Chemistry University of Leeds Woodhouse Lane Leeds LS2 9JT UK; ^3^ SuperSTEM Laboratory SciTech Daresbury Campus Keckwick Lane Daresbury WA4 4AD UK; ^4^ School of Chemical and Process Engineering & School of Physics University of Leeds Woodhouse Lane Leeds LS2 9JT UK; ^5^ Department of Energy Science and Engineering Kunsan National University 558 Daehak‐ro Gunsan‐si Republic of Korea; ^6^ Institute of Physics University of Graz Universitätsplatz 5 Graz 8010 Austria; ^7^ Department of Materials Science University of Milan Bicocca via Roberto Cozzi 55 Milano 20125 Italy

**Keywords:** C─S Bond formation, Cross‐coupling reactions, Density functional theory, Fine chemical synthesis, Single‐atom catalysis

## Abstract

Carbon‐heteroatom cross‐coupling reactions have become indispensable tools in synthetic chemistry. However, the formation of carbon–sulfur (C─S) bonds, which are essential for producing thioethers used in pharmaceuticals, agrochemicals, and advanced materials, remains significantly underdeveloped. Industrial C─S coupling methods still rely on expensive, homogeneous catalysts that suffer from poor recyclability and are susceptible to sulfur‐induced deactivation. In this work, we report a copper single‐atom catalyst, where Cu sites are atomically dispersed on mesoporous graphitic carbon nitride, to enable efficient, selective, and recyclable C─S cross‐coupling reactions under mild conditions and on a gram scale. The catalyst exhibits excellent resistance to thiol poisoning and maintains high performance over multiple catalytic cycles. Advanced characterization techniques, including aberration‐corrected electron microscopy, X‐ray absorption spectroscopy, and single‐atom‐sensitive electron energy loss spectroscopy, confirm the atomic dispersion and stable coordination environment of Cu sites. Combined with density functional theory simulations and radical scavenging experiments, our mechanistic investigations support a concerted oxidative addition pathway, which excludes radical intermediates. These results provide key insights into heterogeneous C─S coupling and demonstrate the power of single‐atom catalysts in addressing long‐standing challenges in sulfur chemistry, paving the way toward greener and more scalable processes for fine chemical and pharmaceutical synthesis.

## Graphical abstract

We report the first single‐atom catalyst for C─S cross‐coupling, featuring atomically dispersed Cu sites on mesoporous carbon nitride. The catalyst enables efficient, selective, and recyclable thioether synthesis under mild conditions and on a gram scale. The catalyst resists sulfur poisoning and operates via a concerted, non‐radical mechanism, enabling a novel, scalable, and sustainable method for pharmaceutically relevant thioethers.

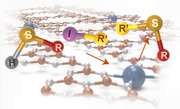

## Introduction

Carbon‐heteroatom cross‐coupling reactions are vital to contemporary synthetic chemistry, facilitating C─N, C─O, and C─S bond formation. Among these, C─S cross couplings present particular challenges, as sulfur nucleophiles readily undergo oxidation and other competing reactions.^[^ ^1^ ^]^ Despite these hurdles, ongoing research in this area remains essential, as thiocouplings provide access to thioethers—core structural motifs in natural products such as amino acids, vitamins, and antibiotics, all renowned for an eclectic spectrum of biological activity.^[^ ^2^ ^]^ Consequently, thioethers serve not only as integral components of numerous active pharmaceutical ingredients (APIs),^[^ ^3^, ^4^ ^]^ but also as precursors to sulfonamide and sulfone functionalities and as versatile synthetic handles (e.g., in photocleavable protecting groups).^[^ ^5^ ^]^ Beyond pharmaceuticals, thioethers also see extensive use in soft materials, where they are frequently utilized as functional fragments and precursors for polymerization,^[^ ^5^ ^]^ play roles in semiconductor fabrication,^[^ ^6^ ^]^ and find a broad array of scientific and industrial applications.^[^ ^7^ ^]^

Given the permeating reach of thioethers in modern society, synthetic chemists are continuously refining methods for their formation. Consequently, a wide range of strategies have emerged that are often described as sustainable, encompassing diverse starting materials and reaction conditions as well as photo‐ and metal‐free catalysis.^[^ ^8^, ^9^ ^]^ However, while these approaches hold promise, they still face significant challenges such as costly or toxic homogeneous (photo)catalysts prone to degradation, limited thiol precursor accessibility, and the requirement for stoichiometric reagents.^[^ ^10^, ^11^, ^12^, ^13^, ^14^ ^]^

Owing to these constraints, the direct transition metal‐catalyzed C─S coupling of aryl halides with thiols (or other organosulfur surrogates) remains the most common strategy for thioether synthesis, particularly at scale.^[^ ^15^ ^]^ Over the past 50 years, this process has evolved from relying on homogeneous palladium catalysts to incorporating less toxic, earth‐abundant metals such as nickel and copper.^[^ ^15^, ^16^, ^17^ ^]^ The transition to heterogeneous catalysis at the dawn of the 21^st^ century further modernized the field,^[^ ^18^ ^]^ facilitating catalyst separation, recyclability, and minimizing metal contamination—imperatives for pharmaceutical production. In this context, copper (II) oxide—in bulk, powder, or nanoparticle form—has gained prominence due to its low cost and efficacy.^[^ ^19^ ^]^

Nonetheless, a pressing need remains for more robust, sustainable catalysts, as thiols’ strong coordinating capacity can deactivate catalytic sites. Although several studies have demonstrated promising CuO nanoparticle reuse, others report performance losses over repeated catalytic cycles, highlighting the importance of developing more durable and stable catalysts.^[^ ^18^ ^]^ Moreover, while palladium‐based mechanisms in C─S couplings are well‐established, copper pathways remain contentious, with proposals including redox cycles and single‐electron transfer (SET) processes.^[^ ^9^, ^20^ ^]^ The complexity of nanoparticle‐based catalysts, featuring dynamic and multifaceted surfaces, complicates mechanistic interpretations.^[^ ^21^, ^22^ ^]^ As a result, many studies on heterogeneous thiocouplings either lack detailed mechanistic insights or present conflicting interpretations.^[^ ^18^ ^]^

To overcome these challenges and advance sustainable thioether synthesis, we developed a copper single‐atom catalyst (SAC) tailored to C─S cross coupling reactions. In this system, copper atoms are atomically dispersed across a mesoporous graphitic carbon nitride (mpgCN*_x_*) matrix, achieving maximal metal utilization and a uniform, well‐defined active site architecture. This design combines the hallmark activity and selectivity of homogeneous catalysts with the scalability and robustness of heterogeneous systems, yielding a catalyst with superior performance metrics across activity, selectivity, and durability. SACs represent a transformative frontier in catalysis, delivering complete metal atom economy, tunable electronic structures, and highly accessible active sites that collectively drive exceptional reactivity.^[^ ^23^, ^24^ ^]^ Their atomically dispersed nature and strong metal‐support interactions confer unique resistance to catalyst deactivation, including, in certain instances, sulfur poisoning.^[^ ^25^ ^]^ For instance, a Cu_1_/CeO*_x_* catalyst was reported for S‐arylation reactions leading to diaryl disulfide formation.^[^ ^26^ ^]^ Similarly, a system based on Ni embedded within the metal‐organic framework (MOF) UiO‐66 gave early indications for C─S coupling reactions.^[^ ^27^ ^]^ Nonetheless, the absence of direct spectroscopic evidence about the formation of single‐atom catalysts limits the possibility to unambiguously confirm the presence and coordination environment of isolated sites. Moreover, metals at framework nodes within MOFs (even where coordinatively unsaturated) may not be consistently accessible in catalysis. In contrast, mesoporous carbon nitrides are ideal for stabilizing single atoms on their surfaces due to their nitrogen‐rich, uniform structure, which ensures strong metal‐support interactions, enhances diffusion, reduces thiol accumulation, prevents atom clustering, and maintains high catalyst activity even at elevated loadings.^[^ ^28^ ^]^ Our Cu SAC is expected—and was experimentally shown—to maintain strong activity and selectivity over repeated thiocoupling catalytic cycles, even at gram scale. The structural homogeneity of the active sites further promotes exceptional chemoselectivity, while the precise coordination environment around each copper active center offers unprecedented mechanistic insight into C─S coupling pathways. Consequently, our findings lay essential groundwork for the next generation of robust catalysts that meet the industrial demand for efficient, scalable thioether synthesis.

## Materials and Methods

### Catalyst Synthesis

The Cu SAC was synthesized via a literature reported hard‐template polymerization protocol and named Cu_1_@mpgCN*_x_*.^[^ ^29^ ^]^ Cyanamide (3 g; Sigma–Aldrich, 99%) and Cu(II) chloride dihydrate (0.6 mmol; Sigma–Aldrich, 99%) were dissolved in an aqueous suspension of silica nanospheres (7.5 g; Sigma–Aldrich, 40% SiO_2_, Ludox HS‐40 in H_2_O) and stirred at 80 °C for 16 h. The obtained solid was finely ground and calcined in air at 520 °C for 3 h, with a temperature ramp of 2.2 °C min^−1^. Subsequently, the thermally treated material was further ground and dispersed in a 4.2 M NH_4_HF_2_ solution (12 g in 50 mL of Milli‐Q water; Sigma–Aldrich, 95%). The dispersion was stirred at room temperature for 24 h followed by vacuum filtration, washing with Milli‐Q water (150 mL) and ethanol (50 mL), and vacuum‐drying at 65 °C overnight. The bare organic support (mpgCN*_x_*) was prepared similarly, omitting Cu(II) chloride dihydrate.

### Catalyst Characterization

The catalyst's Cu content was determined by inductively coupled plasma optical emission spectroscopy (ICP‐OES) with a Perkin Elmer Optima 8300, equipped with a photomultiplier tube detector. After dissolving the material into a concentrated, strong acidic medium, the solution was nebulized and introduced into an ICP torch. Here, excitation of atoms and ions produces photon emissions at element‐specific wavelengths, enabling the precise identification and quantification of copper content. The carbon, nitrogen, and hydrogen composition of Cu_1_@mpgCN*_x_* and bare mpgCN*_x_* was assessed through conventional combustion analysis using a Vario MICRO Elemental Analyzer. The samples underwent high‐temperature decomposition (>1000 °C), resulting in complete combustion. The evolved gaseous products were separated via gas chromatography and quantified using a thermal conductivity detector. Attenuated total reflectance‐Fourier transform infrared (ATR‐FTIR) spectroscopy was conducted using a Smart iTX accessory mounted on a Thermo Scientific Nicolet iS20 FTIR spectrometer equipped with a deuterated triglycine sulfate (DTGS) detector. The materials were thoroughly ground and deposited on the ATR diamond crystal. For each sample, 128 interferograms acquired at a 4 cm^−1^ resolution were averaged over the 4000–400 cm^−1^ wavenumber range. X‐ray diffraction (XRD) analysis of the powdered Cu_1_@mpgCN*_x_* and mpgCN*_x_* materials was performed using a Bruker D2 Phaser diffractometer equipped with Cu *K*α radiation (λ = 0.15418 nm). Powdered samples were homogeneously positioned on aluminum holders and subjected to the XRD measurement under ambient conditions. The data acquisition spanned a 2θ range of 5°–60°, employing a step size of 0.016° and a counting duration of 0.4 s per step. N_2_ physisorption at −196 °C (3P Sync 400) was used to assess the porosity and surface area of CN*_x_*‐based materials. Samples were outgassed at 150 °C for 24 h to remove moisture and contaminants. Specific surface areas were calculated using the Brunauer–Emmett–Teller (BET) method over the 0.05 < *p*/*p_0_* < 0.3 range of the adsorption isotherm, where monolayer‐to‐multilayer nitrogen adsorption occurs. Solid‐state ^13^C/^15^N NMR cross‐polarization/magic angle spinning nuclear magnetic resonance (CP‐MAS NMR) spectra were acquired on a Bruker Neo spectrometer (11.7 T) with a 4‐mm MAS iProbe. Finely ground catalysts were packed into zirconia rotors and measured under the following conditions: repetition time of 4 s, contact time of 8 ms, and a spinning rate of 10 kHz. Thermal gravimetric analyses (TGA) were performed on a PerkinElmer STA 6000 analyzer in air, heating each sample from 30° to 900 °C at 10 °C min^−1^.

For X‐ray photoelectron spectroscopy (XPS) measurements, the catalyst powder was gently distributed and pressed onto a double‐sided carbon adhesive tape, which was fixed on a flag‐style sample holder, and then introduced into the ultrahigh‐vacuum (UHV) analysis chamber equipped with a dual anode X‐ray source (XR50, Specs) and a hemispherical electron energy analyzer (Argus CU, Scienta Omicron) via a load‐lock. Care was taken to fully cover the carbon tape to ensure that no additional signal contributions from the carbon tape are detected. The XP spectra of the samples were acquired employing an Al *K*α radiation source (1486.7 eV) at normal emission. For the Cu 2*p*, C 1*s*, and N 1*s* detail spectra, the analyzer pass energy was set to 50 eV. Spectral fitting of the Cu 2*p* spectra was performed with the XPS Peak software. Charging effects were considered by relating the binding energy scale to the C 1*s* signal of the aromatic carbon at 284.7 eV.

Ex situ X‐ray absorption spectroscopy (XAS) measurements were carried out at the 10C Wide XAFS beamline (BL10C) of the Pohang Light Source‐II (PLS‐II). The incident monochromatic X‐ray beam was shaped using a Si (111) double crystal monochromator, which rejected harmonics by detuning the beamline optics, reducing the incident beam intensity by 40%. The XAS measurements used 1 mm (vertical) × 5 mm (horizontal) slits, acquired at the Cu *K* edge (8979 eV) in transmission mode with ionization chamber detectors. XAS data were normalized to unity edge jump at the Cu *K* edge using the Athena software from the Demeter suite. After extracting the extended X‐ray absorption fine structure (EXAFS) functions, a Fourier transform was applied to the *k^2^*‐weighted *χ*(*k*) functions in the 2.7–7.5 Å^−1^ *k* range, yielding the final Fourier‐transform EXAFS spectra.^[^ ^17^ ^]^

High‐angle annular‐dark‐field scanning transmission electron microscopy (HAADF‐STEM) imaging and electron energy‐loss spectroscopy (EELS) were conducted on an UltraSTEM100 (Nion Co.) microscope equipped with a cold field emission gun and a quadrupole–octupole aberration corrector in the probe forming optics and operated at 60 keV (31 mrad semi‐angle convergence, 90–190 mrad semi‐angle HAADF detection, 40 mrad semi‐angle EELS collection). The microscope used an Enfina spectrometer (Gatan Inc.) and a Merlin‐Medipix (Quantum Detectors) hybrid‐pixel detector. HAADF‐STEM imaging was collected in two ways: (1) in a “slow‐scan” mode with a dwell time of 36 µs per pixel for single frame acquisition, and (2) in a “continuous scan” mode with a dwell time of 2.5 µs per pixel. Frames during continuous scanning were saved using a custom script in Gatan Microscopy Suite software (Gatan Inc.). During continuous scanning, EELS spectra were acquired simultaneously with 10 ms readouts of the Merlin‐Medipix detector, summed between 5000 and 15 000 detector frames for all spectra shown to improve the signal‐to‐noise ratio in otherwise weak Cu signals from single‐atom dispersions. Images and spectra were processed using HyperSpy 1.7.2 and ImageJ image processing software. For EELS processing in HyperSpy 1.7.2,^[^ ^30^ ^]^ the zero‐loss peak was first aligned using cross‐correlation based routines. Background subtraction was carried out using power‐law background model fitting to the pre‐edge energy loss window for Si and Cu *L_23_* edges. For visualizing the background‐subtracted Cu *L_23_* edge (due to limited signal‐to‐noise ratio), a smoothing filter was applied to plot smoothed and as‐acquired data in overlay. The smoothing was carried out as a one‐dimensional uniform filter (17–18 channels, ∼20 eV moving average) implemented in the SciKit Image Python package. The “continuous scanning” images during EELS acquisitions exhibited low signal‐to‐noise ratios, necessary to minimize beam‐induced changes to the sample. To identify single atom features, a difference of Gaussians method (implemented in the SciKit Image Python package) was employed to filter the slowly varying background from the images. The Matplotlib Python package was utilized for plotting the EEL spectra. Line profiles across single‐atom features in HAADF‐STEM images were extracted through ImageJ.

High‐resolution scanning electron microscopy (SEM) imaging was carried out using an SU‐9000 electron microscope (Hitachi High Tech), operated at 30 keV. The microscope acquired simultaneous secondary electron, HAADF‐STEM, and bright field STEM (BF‐STEM) images.

### Catalytic Experiments

An oven‐dried microwave vial was loaded with base (1.5–2 mmol) and catalyst (10–25 mg), sealed, and maintained under an inert atmosphere. Anhydrous dioxane (3.0 mL), benzyl mercaptan (1 mmol), and aryl halide (1.2–1.5 mmol) were sequentially added. The resulting mixture was stirred at 80–110 °C for 8–12 h. Post‐reaction, the crude was filtered (0.45 µm nylon) and analyzed via HPLC (Agilent 1200, G1315D UV detector, λ = 210 nm) using a Thermo Scientific C18 Hypersil GOLD column (5 µm, 175 Å) with a 60:40 MeCN:H_2_O mobile phase (0.7 mL min^−1^, 40 °C). Substrate scope evaluation was performed under optimized conditions (Cu_1_@mpgCN*_x_*:10 mg, K_2_CO_3_: 1.5 mmol, anhydrous dioxane: 3.0 mL, thiol: 1 mmol, aryl iodide: 1.2 mmol, 110 °C, 8 h). After reaction completion, the mixture was filtered and concentrated in vacuo. Subsequently, it was diluted with H_2_O (5 mL) and extracted with ethyl acetate (3 × 10 mL EtOAc). The combined organic layers were washed with brine (5 mL), dried over MgSO_4_ and concentrated in vacuo. The crude was purified by silica gel column chromatography (ethyl acetate/hexane) to afford the desired products. Catalyst recovery involved filtration, washing (2 × 5 mL EtOAc, 2 × 5 mL H_2_O), and reuse in further reaction cycles.

### Computational Details

Spin‐polarized density functional theory (DFT) calculations were performed with the Vienna ab‐initio simulation package (VASP) code,^[^ ^31^, ^32^ ^]^ using the generalized gradient approximation, as implemented in the Perdew–Burke–Ernzerhof (PBE) functional.^[^ ^33^ ^]^ Dispersion forces were included according to Grimme's D3 parametrization.^[^ ^34^ ^]^ The valence electrons have been expanded on a set of plane waves with a kinetic energy cutoff of 450 eV, whereas the core electrons were treated with the projector augmented wave approach (PAW).^[^ ^35^, ^36^ ^]^ The threshold criteria for electronic and ionic loops were set to 1 × 10^−6^ eV and 1 × 10^−^^2^ eV Å^−1^, respectively. The sampling of the reciprocal space was reduced to the gamma point because of the cell size. Single‐point PBE0 calculations were performed on top of PBE‐optimized structures to improve the description of the electronic structure.^[^ ^37^, ^38^ ^]^ This strategy enabled us to avoid intensive geometry optimizations with hybrid functionals with an acceptable error bar of about 0.1 eV.^[^ ^39^ ^]^ As a catalyst, we considered a newly elucidated CN*_x_*‐supported‐structure recently validated through experimental results and DFT simulations.^[^ ^40^ ^]^ The optimized lattice parameters for the supporting framework are: *a* = 15.593 Å, *b* = 20.698 Å, γ = 90.2°. Each intermediate's binding energies (Δ*E*) were calculated with respect to the free molecular species and the catalyst. The Gibbs energies (Δ*G*) were evaluated by adopting the thermochemistry approach of Nørskov and co‐workers, including zero‐point energy correction and entropy terms.^[^ ^41^, ^42^, ^43^ ^]^ The latter were calculated within the harmonic approximation by considering the reaction temperature (110 °C), and the entropy of solid‐state species was considered equal to zero.^[^ ^43^ ^]^

## Results and Discussion

### Catalyst Structure

**Figure 1:**
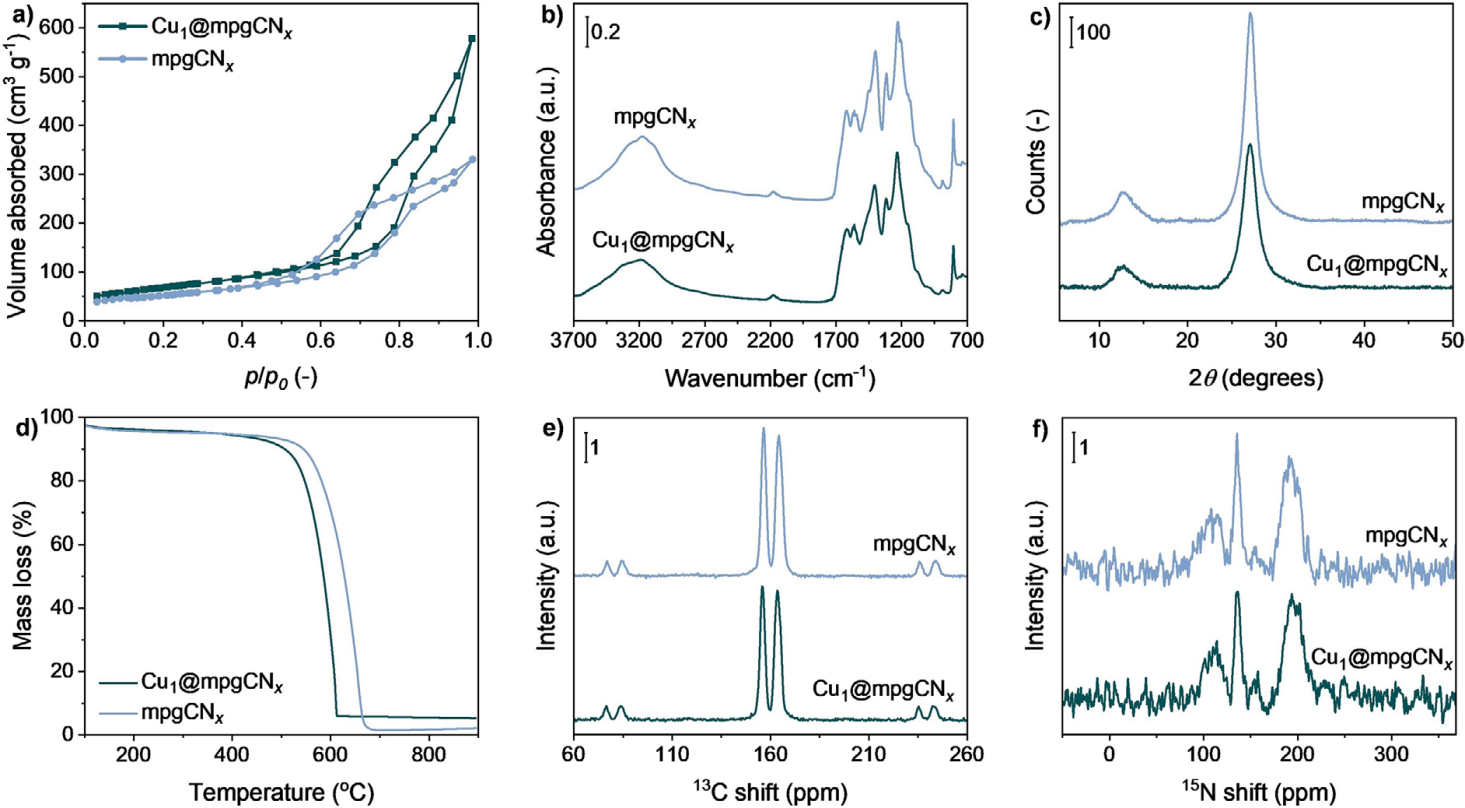
a) N_2_ adsorption isotherms, b) ATR‐FTIR spectra, c) XRD patterns, d) TGA curves, e) ^13^C and f) ^15^N CP/MAS NMR spectra of Cu_1_@mpgCN_*x*_ and metal‐free mpgCN_*x*_.

**Figure 2:**
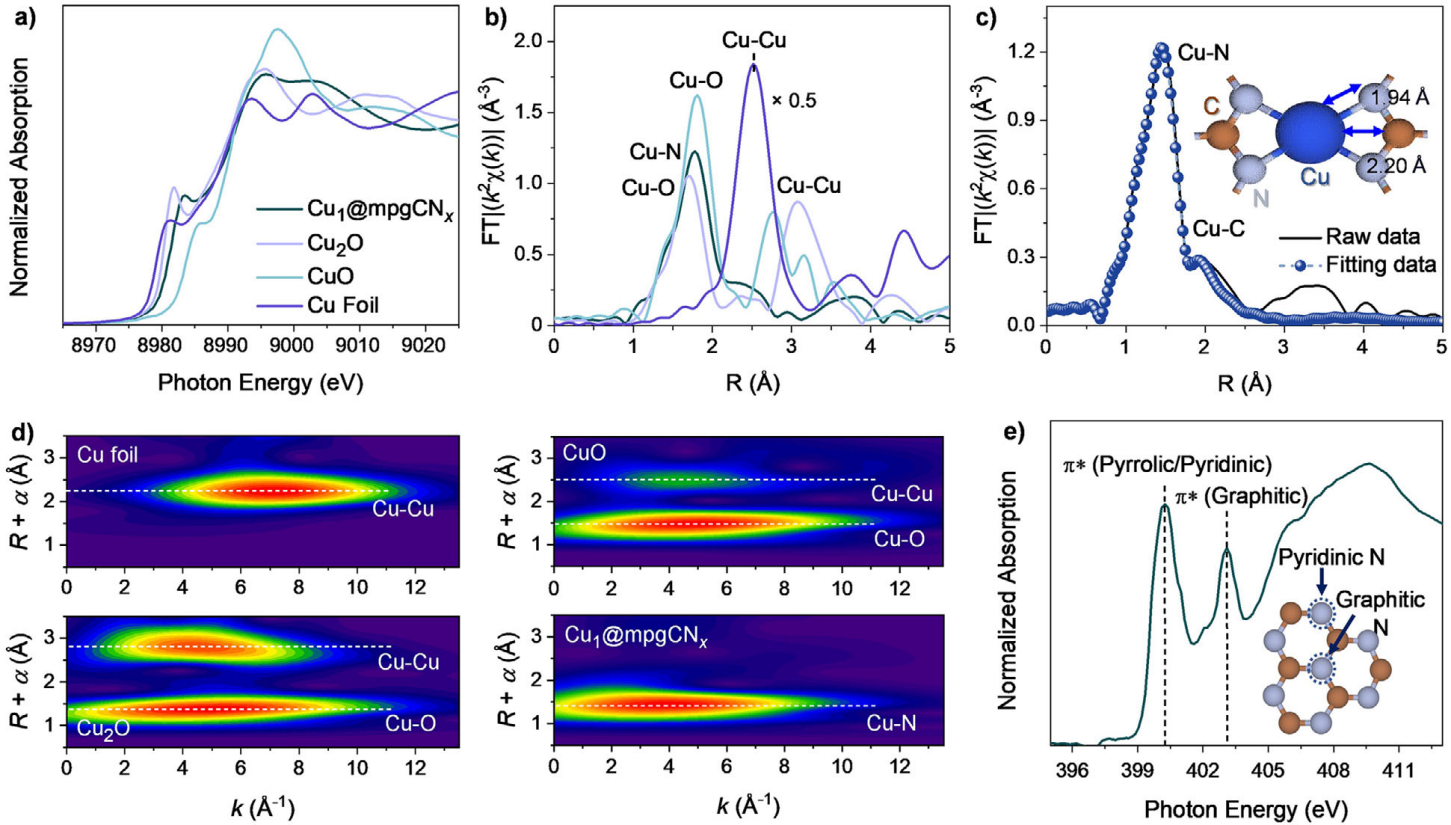
a) XANES data for Cu *K*‐edge and b) phase‐corrected FT‐EXAFS data for Cu foil, CuO, Cu_2_O, and Cu_1_@mpgCN_*x*_. c) FT‐EXAFS fitting results for Cu_1_@mpgCN*_x_*. d) WT‐EXAFS data for Cu foil, CuO, Cu_2_O, and Cu_1_@mpgCN*_x_*. e) N *K*‐edge XAS spectra of the Cu_1_@mpgCN*_x_* sample. The inset in c) reports the model structure of the CN*_x_* cavity hosting Cu single‐atoms, and employed in the FT‐EXAFS fitting process.

The pristine mpgCN*_x_* support and Cu_1_@mpgCN*_x_* catalyst, containing individually dispersed Cu atoms, were synthesized via a hard‐template preparation strategy.^[^ ^29^ ^]^ Silica nanospheres were employed as a template and subsequently removed to produce mesopores, thereby increasing the surface area of the materials and allowing for enhanced exposure of the active sites to the reaction mixture. This strategy enables anchoring of Cu atoms into the nitrogen‐rich CN*_x_* matrix, which acts as a well‐defined coordination scaffold and provides a degree of control over the local coordination environment of the Cu centers. Extensive characterization of the materials was conducted through a combination of a wide set of analytical techniques, which provided comprehensive insights into the chemical composition, electronic, and structural properties of the materials. N_2_ adsorption‐desorption measurements on both the mpgCN*_x_* support and Cu_1_@mpgCN*_x_* revealed characteristic type IV isotherms, consistent with the presence of mesoporous structures, accompanied by a pronounced hysteresis loop indicative of capillary condensation (Figure 1a).^[^ ^44^, ^45^ ^]^ Specific surface area analysis, calculated by resorting to the BET method, yielded values of 184 m^2^ g^−1^ for mpgCN*_x_* and 240 m^2^ g^−1^ for Cu_1_@mpgCN*_x_*. These results further confirmed the porous nature of the support and demonstrated that incorporating Cu species does not compromise its structural integrity. The C/N ratios of Cu_1_@mpgCN*_x_* and mpgCN*_x_* were determined to be within the range of 0.60–0.65, in agreement with the values documented in the literature for CN*_x_* materials synthesized through similar methodologies (Table S1).^[^ ^46^ ^]^ Variations in the C/N ratios have been frequently attributed to structural defects, which were here introduced during the thermal polymerization process.^[^ ^40^, ^47^, ^48^, ^49^ ^]^ The presence of such structural imperfections was further supported by the detection of trace amounts of hydrogen in the catalysts, which would not be expected if the polymerization process were fully completed. ICP‐OES measurements on Cu_1_@mpgCN*_x_* confirmed the successful incorporation of Cu species into the mesoporous CN*_x_* framework (Cu = 2.5 wt%). ATR‐FTIR spectroscopy was used to investigate the chemical nature of the organic support of the investigated materials. The infrared spectra of the two CN*_x_*‐based catalysts displayed characteristic stretching and bending vibrational modes associated with the tri‐*s*‐triazine units that form the core of the CN*_x_* framework (Figure 1b).^[^ ^40^, ^48^, ^49^ ^]^ The numerous infrared peaks observed between 1650 and 1350 cm^−1^ were attributed to single C─N or double C═N bonds within the support.^[^ ^50^, ^51^ ^]^ A broad absorption band between 3000–3500 cm^−1^ was assigned to O─H or N─H stretching vibrations, possibly originating from adsorbed water or unpolymerized amino groups at the edges of the CN*_x_* structure.^[^ ^52^, ^53^ ^]^ Additionally, the out‐of‐plane bending mode typical of the CN*_x_* aromatic units was observed at ca. 800 cm^−1^, further supporting the presence of tri‐*s*‐triazine motifs.^[^ ^29^, ^54^ ^]^ These observations confirmed that the incorporation of Cu species into the mpgCN*_x_* framework did not cause significant structural rearrangement or chemical disruption of the CN*_x_* bonding network. The phase composition and crystallinity of Cu_1_@mpgCN*_x_* and metal‐free mpgCN*_x_* were investigated by XRD measurements. The diffraction patterns displayed in Figure 1c exhibited prominent peaks at 2θ = 27° and 13°, mainly corresponding to the (002) and (100) planes of the graphitic CN*_x_* structure, respectively.^[^ ^55^, ^56^ ^]^ More specifically, the (002) plane is associated with the interlayer stacking of the conjugated aromatic systems, reflecting the layered structure of graphitic‐like architectures. The (100) plane, on the other hand, corresponds to the in‐plane structural packing within the tri‐*s*‐triazine units. The absence of diffraction peaks attributable to crystalline Cu phases, including CuO clusters or nanoparticles, suggested that the metal species in Cu_1_@mpgCN*_x_* are highly dispersed at the atomic level on the support material. TGA was conducted on the powdered catalysts to evaluate their thermal stability (Figure 1d). The resulting curves of Cu_1_@mpgCN*_x_* and mpgCN*_x_* show a first mass loss occurring at ∼100 °C, due to the desorption of water molecules from the materials. Significant mass losses of ca. 80%–90% were observed between 500° and 600 °C, mainly due to the thermal decomposition of the mesoporous support.^[^ ^57^ ^]^ Solid‐state CP/MAS NMR was used to further explore the chemical structure and environment of carbon and nitrogen atoms in Cu_1_@mpgCN*_x_* and its metal‐free counterpart. In the ^13^C NMR spectra of both samples displayed in Figure 1e, two primary peaks were observed at 164 and 156 ppm, typically observed for CN*_x_*‐based materials. More specifically, the 164‐ppm signal was attributed to carbon atoms connected to primary or secondary amino moieties^[^ ^30^, ^58^ ^]^ while the peak observed at 156 ppm was assigned to carbons bonded to three nitrogen atoms devoid of hydrogen, forming the core structure of the tri‐*s*‐triazine units.^[^ ^30^, ^58^ ^]^ For the ^15^N CP/MAS spectrum of Cu_1_@mpgCN*_x_* (Figure 1f), three main signals were detected at 195, 135, and 115 ppm. The strongest intensity, at 195 ppm, corresponds to nitrogen atoms interacting with two carbons in the aromatic framework of the support material.^[^ ^30^ ^]^ Peaks at 135 and 115 ppm were attributed to the presence of nitrogen atoms representing the nucleus of each tri‐s‐triazine unit and aminic groups resulting from incomplete polymerization of CN*_x_*, respectively.^[^ ^30^ ^]^ XPS was employed to investigate the chemical and electronic properties of Cu species in Cu_1_@mpgCN*_x_*. In the Cu 2*p* spectrum of the catalyst (Figure S1c), a broad peak was observed centered at 932.5 eV, consistent with the presence of Cu species in a Cu^1+^ state. To further examine the electronic and local structure of the Cu_1_@mpgCN*_x_*, XAS measurements at the Cu *K* edge were conducted using synchrotron radiation‐based X‐ray sources. As illustrated in Figure 2a, the X‐ray absorption near edge structure (XANES) spectrum provides valuable insights into the local structure of the material, offering a means to investigate the oxidation state and local symmetry of Cu sites. The XANES spectral features of Cu_1_@mpgCN*_x_* entirely differ from those of the reference samples (Cu_2_O, CuO, and Cu foil), suggesting the presence of Cu species in Cu_1_@mpgCN*_x_* characterized by distinct oxidation states and structural configurations compared to the references. The oxidation state was determined by analyzing the differentiated XANES spectra (8980–8990 eV) to locate the photon energy corresponding to the first differential maximum. More specifically, the average electronic state of the metal species in Cu_1_@mpgCN*_x_* was determined to be +1.3, lying between that of Cu species in Cu_2_O (1+) and CuO (2+). Figure 2b shows the phase‐corrected Fourier‐transformed extended X‐ray absorption fine structure (FT‐EXAFS) spectra for the Cu *K* edge, which were obtained by applying a Fourier transform to the XAS signal within the energy range around 1200 eV, starting from the absorption edge. The peaks observed in the FT‐EXAFS spectral dataset between 1.6 and 1.7 Å, similar to those found in Cu_2_O and CuO, can be attributed to Cu─O or Cu─N interactions, indicating that the Cu atoms in Cu_1_@mpgCN*_x_* are primarily coordinated by nitrogen or oxygen atoms. No Cu─Cu contributions were observed in the Cu_1_@mpgCN*_x_* FT‐EXAFS spectrum, while they were detected for the Cu foil, Cu_2_O, and CuO, indicating that the Cu_1_@mpgCN*_x_* lacks the interaction between Cu atoms and predominantly consists of monatomic Cu dispersed on the mpgCN*_x_* support and coordinated to nitrogen species. To ensure a more reliable comparison and strengthen the validity of the EXAFS analysis, additional fitting for both CuO and Cu_2_O reference samples was performed (Figure S6). These two oxides exhibit different first‐shell Cu─O coordination numbers based on their crystal structures: CuO has a coordination number of 4, while Cu_2_O has a coordination number of 2. Further, to gain insight into the local bonding structure of Cu_1_@mpgCN*_x_*, fitting processes were carried out to determine the bond distances and coordination numbers related to the Cu atoms and the corresponding coordinating N species within the CN*_x_* cavity. The results shown in Figure 2c indicated that the Cu─N and Cu─C bond lengths are 1.94 and 2.20 Å, respectively, with corresponding coordination numbers of 4 and 2. These values are consistent with the expected structural configuration depicted in Figure 2c, suggesting that Cu atoms in Cu_1_@mpgCN*_x_* are individually dispersed on the mpgCN*_x_* support and primarily stabilized via Cu─N bonding. The detailed fitting parameters are provided in Table S2. To gain further insight into the bonding environment, wavelet‐transformed EXAFS (WT‐EXAFS) was conducted in *k*‐space and *R*‐space. As shown in Figure 2d, the WT‐EXAFS spectrum of the Cu foil reveals a strong Cu─Cu contribution near 2.2 Å, with a corresponding intensity maximum at 7 Å^−1^ in *k*‐space. In contrast, Cu_2_O and CuO exhibit both the Cu─Cu and Cu─O bonds, with the latter showing their highest intensities in the 4–5 Å^−1^ range of *k*‐space. For the Cu_1_@mpgCN*_x_* sample, a prominent feature appears at 4 Å^−1^ in *k*‐space, indicating that the Cu atoms are likely coordinated with lighter atoms, such as oxygen or nitrogen, rather than Cu clusters or nanoparticles. The mpgCN*_x_* support, which has been designed to host Cu as single atomic species, was further explored using N *K* edge XAS, as shown in Figure 2e. Two distinct peaks were observed at approximately 400 and 403 eV, corresponding to pyridinic and graphitic nitrogen, respectively, both of which are expected to be predominant in the mpgCN*_x_* structured. Pyridinic nitrogen, in particular, with its lone electron pair, plays a critical role in stabilizing the Cu atoms via Cu─N bonds. However, it is important to note that XAS provides averaged information over all Cu sites, and that the high‐temperature synthesis (520 °C) combined with the intrinsic defects of the mpgCN*_x_* substrate could, in principle, lead to some degree of heterogeneity in Cu coordination environments. While our XANES and EXAFS analyses support the formation of atomically dispersed Cu species with predominant N coordination, absolute homogeneity cannot be fully guaranteed.

**Figure 3:**
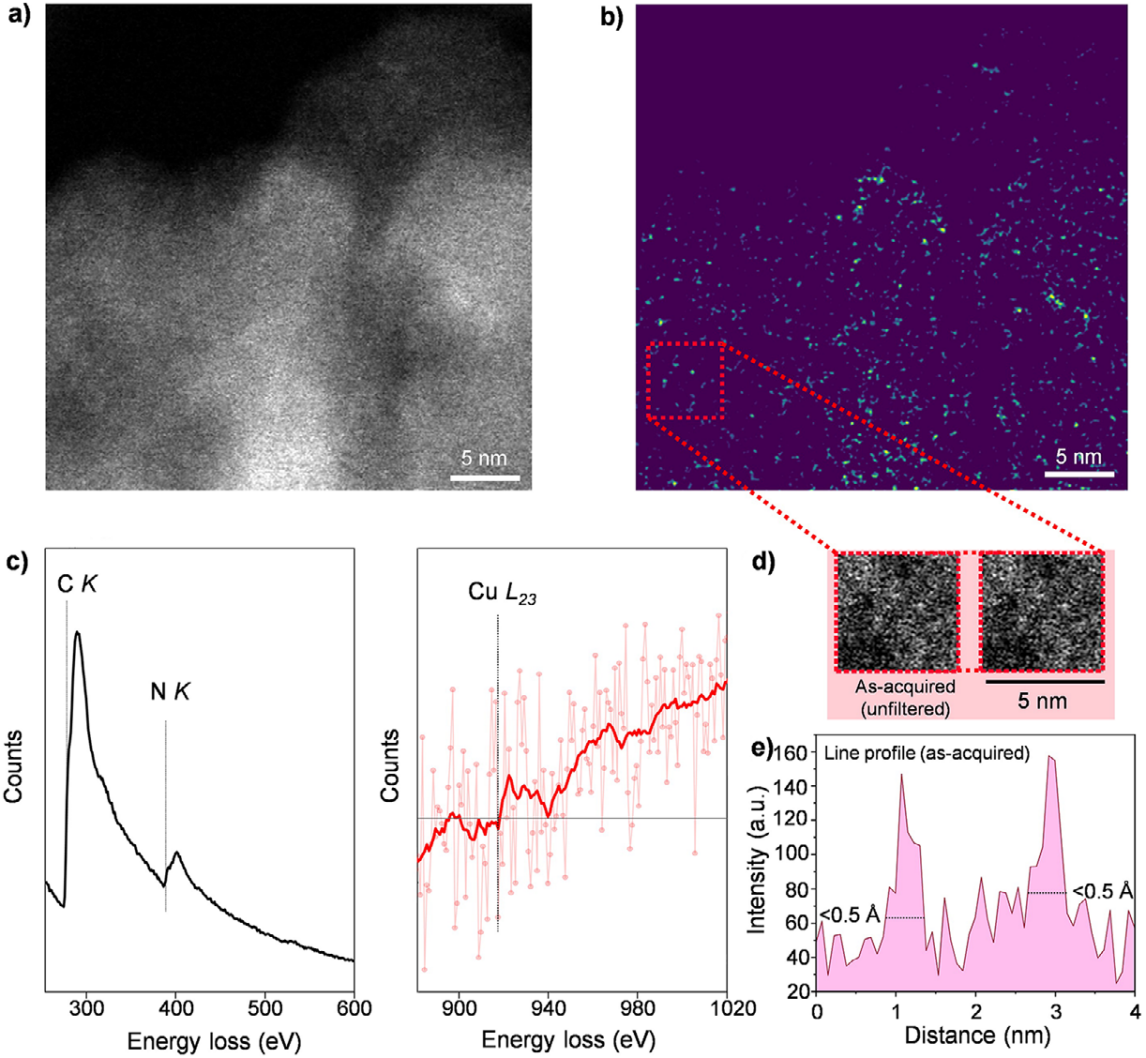
a) Atomically resolved HAADF‐STEM micrograph of Cu_1_@mpgCN*_x_*. b) The same micrograph after application of a difference‐of‐Gaussians filter to remove the slowly varying background and highlight isolated, point‐like image features (single atoms). c) Corresponding EEL spectra collected at the C *K* and N *K* edges (left panel) and the Cu *L_23_* core ionization edge (right panel). The Cu *L_23_* is shown after power law background subtraction. For the Cu *L_23_* spectra, the solid line shows a moving average as a guide to the eye. d) Area in the as‐acquired image corresponding to the area marked by the red dashed square in b). The area is shown twice, with the image on the left un‐annotated for reference and the image on the right highlighting the line profile. e) Corresponding line profile highlighting distinct features of widths less than 0.5 Å, consistent with the expected signal from isolated Cu atoms.

Finally, to further assess the single‐atom dispersion on the CN*_x_* carrier and confirm the identity of the transition metal species, aberration‐corrected HAADF‐STEM and EELS measurements were performed. However, HAADF‐STEM imaging of Cu_1_@mpgCN*_x_* revealed rapid changes in the sample during exposure times typically required for high signal‐to‐noise imaging. Sensitivity to electron exposure, as well as residual surface carbon radiolysis^[^ ^15^ ^]^ under the incident electron beam, led to structural collapse and accumulation of carbon films covering the sample, obscuring single‐atom imaging under conventional conditions. To overcome this challenge, an alternative continuous scanning technique was employed, enabling the collection of sequential HAADF‐STEM frames and simultaneous EELS acquisition over a total readout time exceeding 50 s to retrieve Cu signals at low loadings. Single atoms are weakly visible in the as‐acquired image (Figure 3a) due to the significant intensity variation due to sample thickness variations across the field of view. These were highlighted after applying a difference‐of‐Gaussians filter (Figure 3b). By selecting distinct single atoms in the filtered image (dashed box, Figure 3b), line profiles extracted from the as‐acquired image confirmed the detection of individually isolated atoms above the substrate background intensity (see also Figure 3c). Figure 3d shows the cropped region of an HAADF‐STEM image and a duplicate image with the line profile position reported as a dashed line, with the line profile shown in Figure 3e (dotted lines on the line profiles reveal atom feature widths of less than 0.5 Å). In turn, this multi‐frame HAADF‐STEM image serves to confirm the presence of isolated Cu atoms and rule out any contributions from metal aggregates. No nanoparticles were observed, as supported by high‐resolution SEM imaging, further corroborating that no significant nanoparticle formation occurred (Figure S2a). Instead, clear single‐atom features (<0.5 Å across) were resolved with intensities with peak‐to‐background ratios ∼3 or more in the line profiles (Figure 3e), despite the limited signal‐to‐noise ratio available for imaging the native sample state (Figure 3a). Although the EEL spectra collected during the extended acquisition time showed inherently low signal‐to‐noise ratios due to the low Cu loading, the expected two‐step sequential onset of the Cu *L_23_* edge was detectable (Figure 3c) upon averaging across multiple sample areas (Figures 3 and S3). Additional EEL analyses confirmed the presence of carbon and nitrogen, with negligible contributions from oxygen (O *K* edge at 530 eV energy loss onset) (Figure S3). By integrating advanced spectroscopic techniques such as XAS with high‐resolution imaging via HAADF‐STEM and STEM‐EELS, we conclusively demonstrated the successful incorporation of Cu atoms as monodispersed single atoms homogeneously distributed on the CN*_x_* support.

### Catalytic Performance

At the onset of the catalytic studies, iodobenzene was selected as the model aryl halide, while benzyl mercaptan served as the thiol partner, given its prominence in various pharmaceutical scaffolds.^[^ ^59^ ^]^ Using dimethylformamide (DMF) as the solvent and sodium hydroxide (NaOH) base at 110 °C for 21 h, the reaction afforded a 45% yield of benzyl phenyl sulfide **1a**. Subsequent optimizations focused on varying the solvent, base, time, and temperature to enhance reaction efficiency. Solvent screening revealed that more polar options, namely dimethylacetamide (DMA) and dimethyl sulfoxide (DMSO), achieved yields below 40%. In contrast, less polar solvents proved more effective, with toluene achieving a moderate yield of 60%. Notably, 1,4‐dioxane, characterized by its moderate polarity and low coordinating ability, provided the highest yield (86%), establishing it as the solvent of choice. With the optimal solvent found, we next evaluated the influence of different bases. Substituting NaOH with sodium carbonate (Na_2_CO_3_) severely reduced the yield to 15%, leaving unreacted starting material. Based on this observation, the stronger base potassium hydroxide (KOH) was tested, providing an improved yield of 76%, though still lower than NaOH. Interestingly, further increasing basicity using potassium *tert*‐butoxide (KO*t*Bu) sharply reduced the yield to 10%.^[^ ^60^ ^]^ Completing the base refinement, potassium carbonate (K_2_CO_3_) furnished the desired product within a 91% yield, making it the best base. Temperature optimization revealed that 110 °C was necessary, as lower temperatures led to incomplete starting material conversion. Finally, to further enhance sustainability and reduce reagent consumption, stoichiometric optimization showed that reducing base and iodobenzene equivalents to 1.5 and 1.2, respectively, had minimal impact on yield. Notably, decreasing the catalyst amount to 10 mg maintained high catalytic efficiency. Control experiments with the bare mpgCN*_x_* support and without catalyst (Table 1, Entry 14 and 15) resulted in negligible yields, suggesting that defects and impurities in the support have a minimal contribution to the overall catalytic activity and confirming the essential role of isolated Cu atoms in catalytic activity. Moreover, varying the Cu loading (0.72, 1.4, and 2.5 wt%) resulted in similar yields (86%–91%), indicating minimal influence on performance within this range. Accordingly, Cu loading of 2.5 wt% was selected as the representative composition for subsequent studies.

**Table 1:** Optimization of reaction conditions.

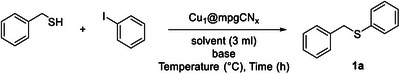					
Entry	Solvent	Base	Time	Temperature (°C)	Yield (%)^a)^
1	DMF	NaOH	21	110	45
2	DMA	NaOH	21	110	33
3	DMSO	NaOH	21	110	35
4	Toluene	NaOH	21	110	60
5	1,4‐dioxane	NaOH	21	110	86
6	1,4‐dioxane	Na_2_CO_3_	21	110	15
7	1,4‐dioxane	KOH	21	110	76
8	1,4‐dioxane	KO*t*Bu	21	110	10
9	1,4‐dioxane	K_2_CO_3_	21	110	91
10	1,4‐dioxane	K_2_CO_3_	8	110	90
11^b)^	1,4‐dioxane	K_2_CO_3_	8	110	91
12	1,4‐dioxane	K_2_CO_3_	8	80	0
13^c)^	1,4‐dioxane	K_2_CO_3_	8	110	90
14^d)^	1,4‐dioxane	K_2_CO_3_	8	110	6
15^e)^	1,4‐dioxane	K_2_CO_3_	8	110	5

^a)^ Product yields calculated via HPLC, using a calibration curve of benzyl phenyl sulphide.

^b)^ Catalyst amount reduced from 50 to 10 mg.

^c)^ Base and iodobenzene equivalents reduced to 1.5 and 1.2, respectively.

^d)^ Bare mpgCN*_x_* was used as catalyst.

^e)^ Reaction without catalyst.

**Figure 4:**
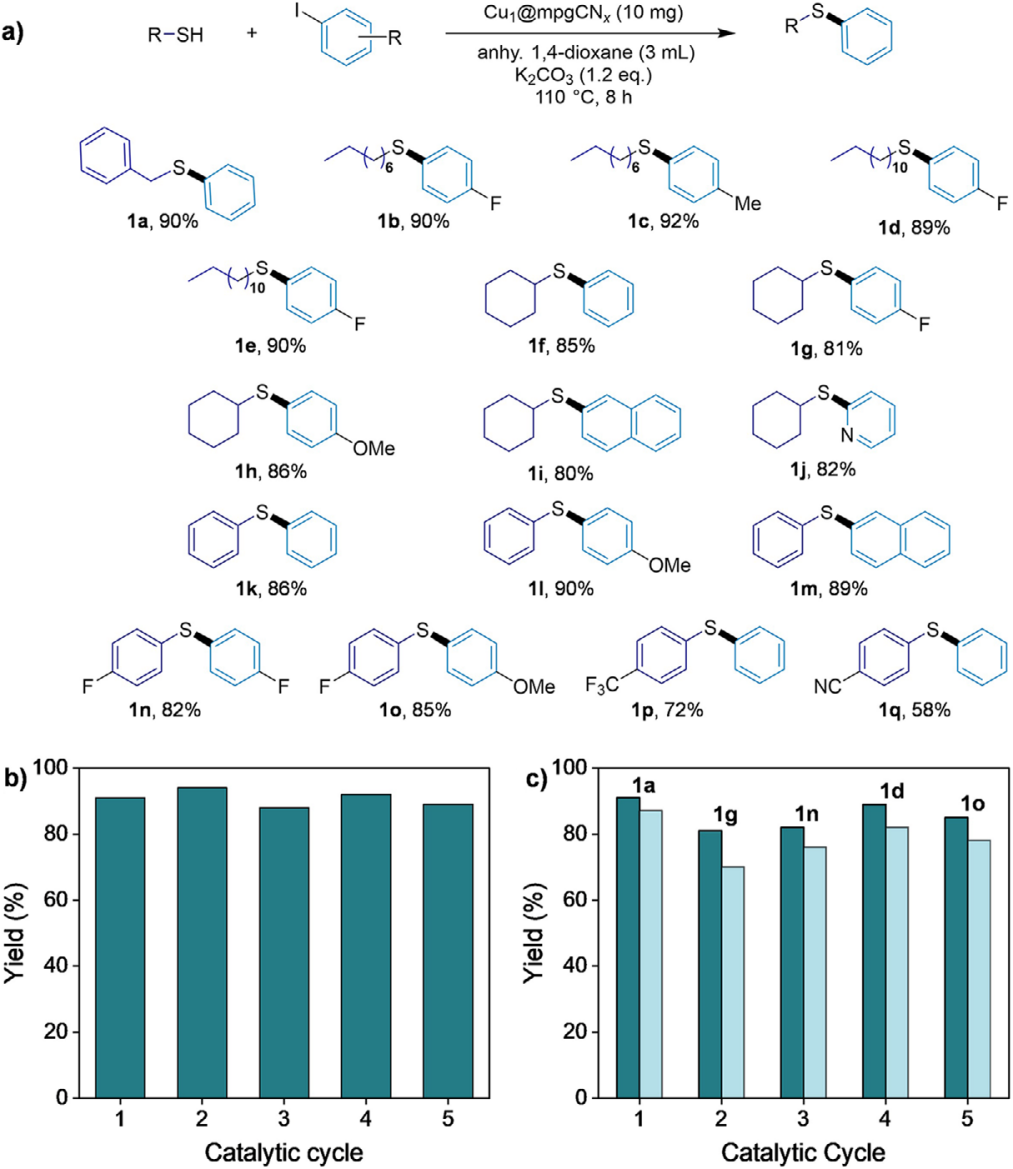
a) Substrate scope and isolated yields for the corresponding C─S coupling reactions. b) Catalyst recycling of Cu_1_@mpgCN_x_ utilizing **1a** as the model product (product yield determined by HPLC analysis). c) Evaluation of Cu_1_@mpgCN*_x_* recycling for different thioether products (product yields by ^19^F NMR spectroscopy using 4‐iodobenzotrifluoride as the internal standard). Color code: deep green = fresh catalyst; light green = recycled catalyst.

With suitable conditions in hand (Table 1), we assessed the reactivity of Cu_1_@mpgCN*_x_* across a balanced substrate scope, including alkyl, cyclic, and aryl thiols with various (hetero)aryl halides, exploring electronic and steric properties (Figure 4a). Tests using chlorobenzene and bromobenzene confirmed the expected reactivity trend, with bromobenzene (63%) exhibiting intermediate reactivity compared to the less reactive chlorobenzene (42%) and the superior iodobenzene (91%). Transitioning to alkyl thiols, these substrates demonstrated facile reactivity regardless of aliphatic chain length or electronic variations in the aryl iodides. Consequently, all alkyl‐aryl thioethers (**1b**‐**1e)** were afforded in excellent yields (89%–92%) within the allotted reaction time. Similarly, substrates of increased molecular complexity, such as cyclohexanethiol, were also efficiently converted to the desired products (**1f**‐**1j**). In these cases, subtle electronic effects became evident, with electron‐deficient aryl iodides (**1g**, 81%) slightly outperforming electron‐rich analogues (**1h**, 86%), likely due to enhanced electrophilicity. Additionally, the reaction protocol smoothly accommodated sterically hindered (**1i**, 80%) and biologically relevant heteroaromatic iodides (**1j**, 82%)^[^ ^61^ ^]^ emphasizing its broad applicability. Finally, we expanded our catalytic evaluation to diaryl thioether formation with diverse aromatic substituents (**1k‐1o**). Diphenyl sulfide (**1k**) was obtained in high yield (86%), while introducing electron‐donating (**1l**) or fused aromatic groups (**1m**) delivered comparable yields of 90% and 89%, respectively. Substrates with electron‐withdrawing groups, both symmetrical (**1n**, 82%) and unsymmetrical (**1o**, 85%), reacted smoothly to give thioethers, including CF_3_ (**1p**, 72%) and CN (**1q**, 58%) group containing products found in drugs like triflupromazine and periciazine, underscoring the broad utility of this catalytic method.

### Distinct Advantages of Single Atom Catalyst in C─S Cross‐Coupling Reactions

Recyclability and scalability are crucial metrics to assess the practical applicability of novel heterogeneous catalysts. To evaluate scalability, the benchmark reaction between benzyl mercaptan and iodobenzene was scaled up fivefold to the gram scale, while maintaining the catalyst loading at 10 mg. Despite the protracted reaction time of 24 h to ensure complete substrate conversion, Cu_1_@mpgCN*_x_* maintained excellent performance, achieving an 87% yield and a turnover number (TON) of 1260.

The significance of implementing SACs in thiocoupling reactions is supported by their exceptional recyclability. Iterative reaction cycles to prepare **1a** were performed under optimized conditions. After each reaction, Cu_1_@mpgCN*_x_* was recovered through centrifugation, washed thoroughly with EtOAc (2 × 5 mL) and H_2_O (2 × 5 mL), and dried before reuse. Pleasingly, the catalyst maintained stable catalytic activity over five consecutive runs (Figure 4b). To further demonstrate the robustness of Cu_1_@mpgCN_x_, the recovered catalyst was applied to consecutive C─S coupling reactions bearing distinct substituents. Following an initial 91% yield with benzyl mercaptan and iodobenzene, the same batch of catalyst was reused to produce cyclohexyl sulfide **1g** (89%), diaryl sulfide **1n** (87%), and finally diaryl sulfide **1o** (84%). The yields obtained closely matched those with fresh catalyst (Figure 4c). Importantly, post‐reaction characterization confirmed the integrity of the catalyst. XRD, BET, and XPS measurements revealed no significant alterations in the catalyst structure, surface area, porosity, or Cu active sites (Figure S4). Finally, ICP‐OES analysis verified the absence of copper leaching, ensuring compliance with stringent metal contamination standards essential for pharmaceutical and industrial applications.

**Figure 5:**
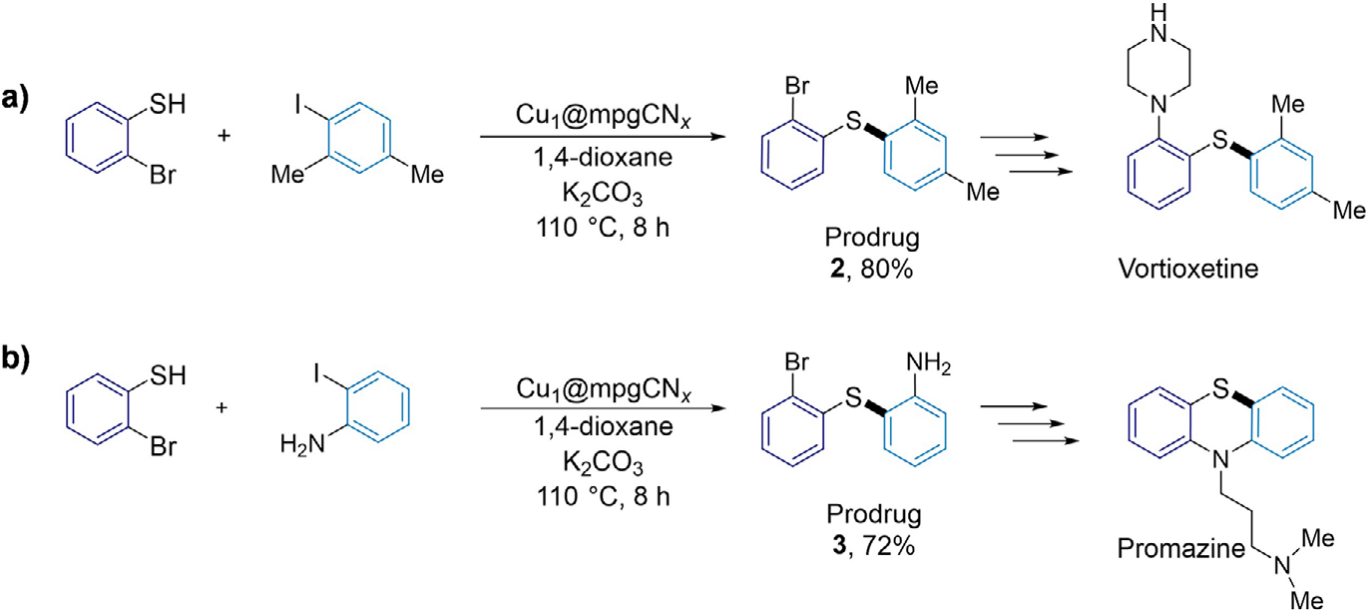
Application of the catalyst in a) vortioxetine and b) promazine synthesis.

The results obtained thus far demonstrate the strong predilection of our catalyst in the synthesis of model thioethers, greatly expanding access to versatile thioether synthetic building blocks. To showcase the aptitude of Cu_1_@mpgCN*_x_* in industrially relevant contexts, we targeted key precursors for two clinically relevant drugs (Figure 5). First, the crucial C─S coupling between commercially available 2‐bromobenzenethiol and 2,4‐dimethyliodobenzene efficiently provided a precursor to the antidepressant vortioxetine (**2**) in 80% yield (Figure 5a), matching recent literature methods while benefiting from an air‐stable catalyst and commercial substrates.^[^ ^14^ ^]^ Similarly, Cu_1_@mpgCN*_x_* effectively catalyzed the synthesis of a phenothiazine‐based intermediate (**3**, 72%) under optimized conditions, a critical building block for antipsychotic promazine (Figure 5b).^[^ ^62^ ^]^

### Mechanistic Insights into Cu SAC‐Catalyzed C─S Couplings

**Figure 6:**
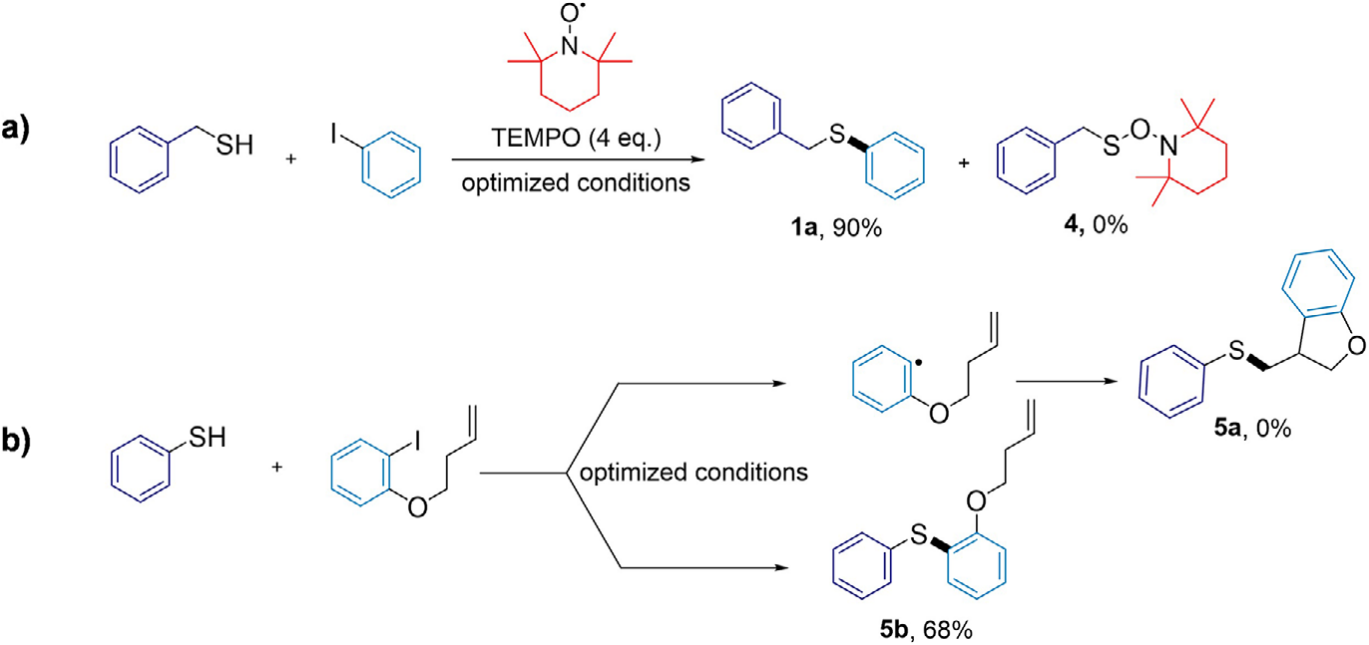
Visual representation of the radical probe experiments conducted to elucidate the electron transfer pathway in the C─S cross‐coupling reaction with the Cu catalyst using a) TEMPO as a radical scavenger and b) a radical clock experiment with 1‐(but‐3‐en‐1‐yloxy)‐4‐iodobenzene.

The inherent structural variability and dynamic behavior of nanoparticles often complicate mechanistic studies of catalytic processes. In contrast, SACs—despite minor coordination and support defects, feature atomically dispersed active sites that provide enhanced uniformity and enable more straightforward mechanistic investigation. To elucidate the mechanism underlying the Cu‐catalyzed C─S protocol, a combination of experimental probes (Figure 6) and theoretical analysis was employed. To exclude the formation of intermediate aryl radical species during catalytic turnover, two radical experiments were conducted. Under standard reaction conditions, the introduction of a fourfold excess of TEMPO (2,2,6,6‐tetramethylpiperidine *N*‐oxide) as a radical scavenger did not affect product formation, with potential product **4** not detected (Figure 6a). Additional support for a mechanism favoring concerted oxidative addition was provided by a radical clock experiment using the olefinic substrate 1‐(but‐3‐en‐1‐yloxy)‐4‐iodobenzene. If a radical intermediate were generated via C─I bond cleavage, rapid intramolecular cyclization would be expected, yielding dihydrobenzofuran **5a**. Instead, the exclusive formation of the uncyclized product (**5b**) in 68% yield was obtained, reinforcing a pathway consistent with concerted oxidative addition and excluding radical involvement (Figure 6b).

DFT calculations were conducted to elucidate the reaction mechanism of the C─S coupling between iodobenzene and benzyl mercaptan catalyzed by Cu single atoms anchored to the CN*_x_* framework. Figure S5a,b present the side and top views of the Cu_1_@mpgCN*_x_* catalyst, where the Cu atom is coordinated with four nitrogens with an average bond length of 2.11 Å. In its ground state, the Cu species adopts a +1 oxidation state, in agreement with XAS and XPS measurements. This is further corroborated by the absence of spin density at the Cu center, a Bader charge of +1.1 e, and DOS curves showing 4s orbital as empty at high energy values. From the eigenvalues, the bandgap excitation of Cu_1_@mpgCN*_x_* is 1.1 eV (Figure S5c). The spin isosurface distribution of Cu_1_@mpgCN*_x_* (Figure S5d) indicates a largely delocalized charge over the support, with partial localization on the Cu orbitals.

**Figure 7:**
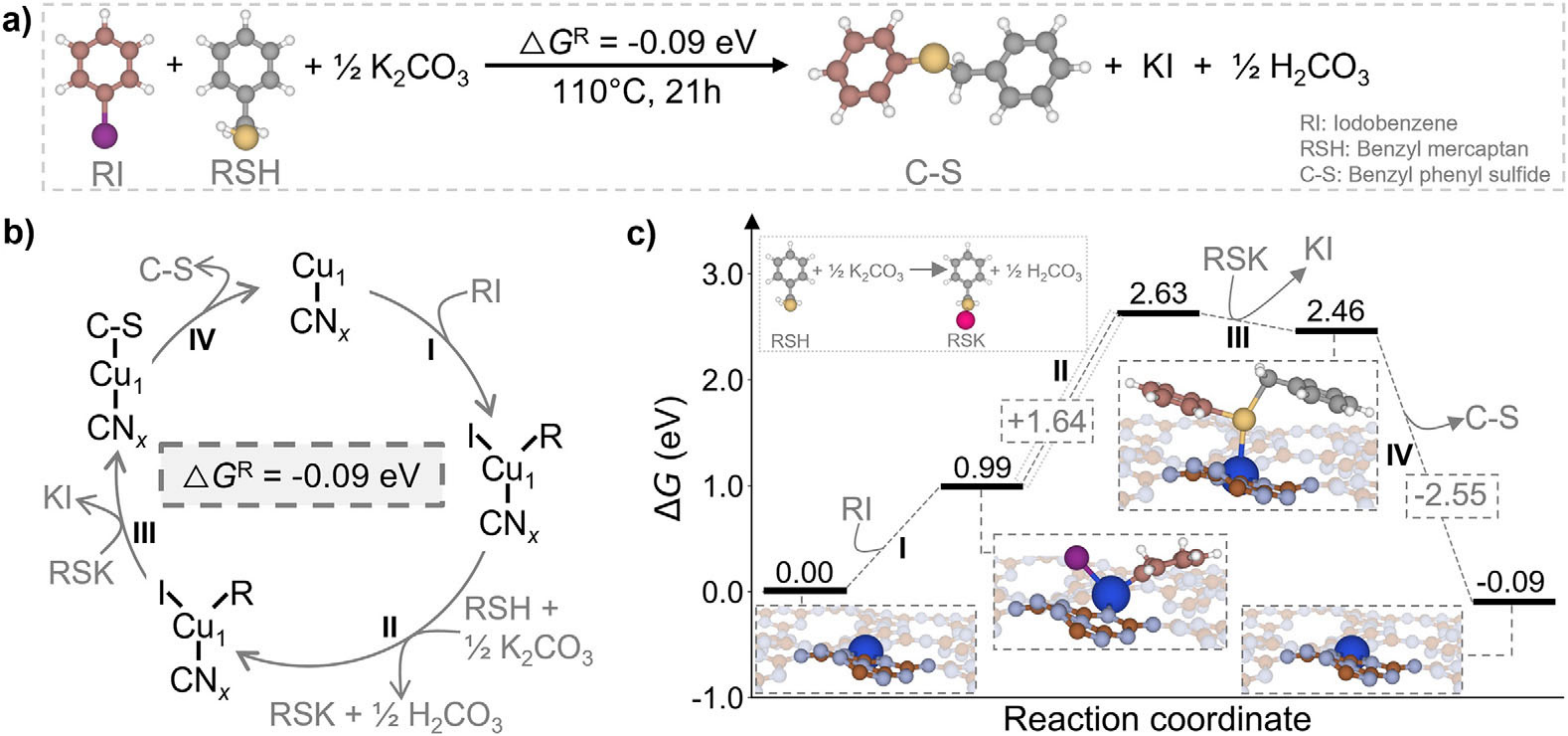
a) Global reaction for the C─S coupling between RI and RSH in the presence of K_2_CO_3_. b) Proposed mechanistic cycle for the C─S coupling reaction. c) Energetic profile for the different intermediates formed during the reaction.

Guided by experimental observations and reaction conditions reported in Table 1, a plausible mechanism for the C─S coupling was proposed, encompassing iodobenzene (RI), benzyl mercaptan (RSH), and K_2_CO_3_, identified as the most effective base for producing the C─S coupled product (**1a**). The overall reaction, illustrated in Figure 7a, yields benzyl phenyl sulfide (C─S), carbonic acid (H_2_CO_3_), and potassium iodine (KI). The organic transformation is slightly exergonic, with a computed Gibbs free energy change (Δ*G*) of −0.09 eV, and proceeds through four consecutive steps, as detailed in the following equations and Figure 7b.

(Step I) $$*\;+\;\mathrm{RI}\;\to RI^{*}\left(\mathrm{IM}1\right)$$

(Step II) $$\mathrm{RSH}+\frac{1}{2}K_{2}{\mathrm{CO}}_{3}\to \mathrm{RSK}\left(\mathrm{IM}2\right)+\frac{1}{2}H_{2}{\mathrm{CO}}_{3}$$

(Step III) $$\mathrm{IM}1+\mathrm{IM}2\to C-S^{*}\left(\mathrm{IM}3\right)+\mathrm{KI}$$

(Step IV) IM3→∗+C−S

In the first step of the proposed mechanism, RI reacts with the Cu_1_@mpgCN*_x_* catalyst (*) to form the first intermediate (IM1). This step is endergonic and requires an energetic cost of 0.99 eV. During the formation of IM1, the coordination of the Cu atom is altered, resulting in a slight shortening of the Cu─N bond lengths from 2.12 Å in the unbound catalyst to 2.06 Å. The newly formed Cu─I and Cu─C bonds measure 2.67 and 1.96 Å, respectively. The second step occurs indirectly and involves the reaction of RSH with K_2_CO_3_ to form the second intermediate (IM2) along with the byproduct H_2_CO_3_. This transformation requires overcoming an energetic barrier of 1.64 eV. In the third step, the interaction between IM1 and IM2 leads to the formation of the C─S coupling intermediate (IM3), accompanied by the release of KI. This step is exergonic, with a free energy of −0.17 eV. In IM3, the sulfur atom establishes a weak interaction with the Cu center at a bond length of 2.88 Å, while the catalyst regains coordination to four nitrogen atoms. The final step is a highly favorable process (Δ*G* = −2.55 eV) and results in the release of benzyl phenyl sulfide (C─S) and regeneration of the free catalyst. A schematic representation of the proposed reaction pathway is provided in Figure 7b, and the corresponding energy profile is illustrated in Figure 7c.

Additional DFT calculations were conducted to investigate the adsorption energy of sulfur species on the Cu SAC by comparing two different environments, namely (i) Cu coordinated to four nitrogen atoms (Cu─N_4_) and (ii) Cu coordinated to three nitrogen atoms and one carbon (Cu─N_3_C_1_). The results indicate that a nitrogen‐rich environment around the Cu atom may electronically shield the copper site (Cu─N_4_), making it less prone to strong sulfur adsorption and thus reducing the risk of catalyst poisoning. The formation energy of the Cu─S bond in the Cu─N_4_ environment (+3.43 eV) is even higher than values reported in a previous study on S poisoning on SACs,^[^ ^63^ ^]^ which suggests that our Cu_1_@mpgCN*_x_* catalyst has resistance to sulfur poisoning.

## Conclusion

In summary, a Cu‐based SAC was successfully designed and synthesized to efficiently promote C─S coupling reactions. Extensive characterization employing advanced spectroscopic and microscopic methods confirmed the uniform distribution of isolated Cu sites anchored onto a mesoporous CN*_x_* support. The resulting Cu catalyst exhibited excellent activity, selectivity, and stability across multiple catalytic cycles, including gram‐scale operations. Catalytic screening demonstrated its versatility, enabling efficient coupling of diverse aryl, alkyl, and cyclic thiols with (hetero)aryl halides. Furthermore, the catalyst's practical applicability was further validated through the synthesis of key intermediates relevant to pharmaceutical manufacturing, underscoring its potential for industrial‐scale processes. The unique single‐atom configuration was identified as critical for mitigating thiol‐induced catalyst deactivation, a common limitation in conventional heterogeneous Cu systems. Mechanistic investigations combining radical probe experiments and theoretical calculations provided clear evidence for a concerted oxidative addition pathway, devoid of radical intermediates. Collectively, these findings establish a promising platform for overcoming long‐standing challenges in C─S bond formation using a novel Cu SAC system, paving the way for next‐generation catalytic systems in pharmaceutical and materials science applications.

## Supporting Information

Supporting information for this article is given via a link at the end of the document. The Supporting Information includes additional characterization and catalytic results of the materials; EXAFS fitting results; density of states and supplementary DFT calculations; and NMR characterizations of the reaction products.

## Conflict of Interests

The authors declare no conflict of interest.

## Supporting information



Supporting Information

## Data Availability

The data that support the findings of this study are available from the corresponding author upon reasonable request.
