# Supplementary material for: Copper Single‐Atom Catalyst for Efficient C─S Coupling in Thioether Synthesis
Source: Angew Chem Int Ed Engl. 2025 Aug 6;64(38):e202510632. doi: 10.1002/anie.202510632 (PMC12435431; doi:10.1002/anie.202510632)
Supplement: Supplementary file 1 — Supporting Information [file ANIE-64-e202510632-s001.pdf]

Supporting Information

Copper Single-Atom Catalyst for Efficient C–S Coupling in Thioether Synthesis

Theodore A. Gazis,[†,a] Shilpa Palit,[†,a] Luis A. Cipriano,[a] Nicolò Allasia,[a] Sean M. Collins,[b,c] Quentin M. Ramasse,[c,d] Ik Seon Kwon,[e] Martin Sterrer,[f] Giovanni Di Liberto,[g] and Gianvito Vilé,[a]*

[a] *Department of Chemistry, Materials, and Chemical Engineering "Giulio Natta", Politecnico di Milano, Piazza Leonardo da Vinci 32, 20133 Milano, Italy.*

[b] *Bragg Centre for Materials Research, School of Chemical and Process Engineering and School of Chemistry, University of Leeds, Woodhouse Lane, LS2 9JT Leeds, United Kingdom.*

[c] *SuperSTEM Laboratory, SciTech Daresbury Campus, Keckwick Lane, WA4 4AD Daresbury, United Kingdom.*

[d] *School of Chemical and Process Engineering and School of Physics, University of Leeds, Woodhouse Lane, LS2 9JT Leeds, United Kingdom.*

[e] *Department of Energy Science and Engineering, Kunsan National University, 558 Daehak-ro, Gunsan-si, Republic of Korea.*

[f] *Institute of Physics, University of Graz, Universitätsplatz 5, 8010 Graz, Austria.*

[g] *Department of Materials Science, University of Milan Bicocca, via Roberto Cozzi 55, 20125 Milano, Italy.*

† Theodore A. Gazis and Shilpa Palit contributed equally to this work.

** Corresponding author. E-mail: gianvito.vile@polimi.it (Prof. Dr. Gianvito Vilé).*

Table S1. *Elemental composition and textural properties of mpgCN$_x$ and Cu$_1$@mpgCN$_x$.*

**Catalyst**	**C[a] (wt %)**	**N[a] (wt %)**	**H[a] (wt %)**	**C/N (-)**	**Cu[b] (wt.%)**	**S_{BET}[c] (m^2 g^{-1})**	**d_{pore}[d] (nm)**
mpgCN$_x$	31.90	48.75	2.43	0.65	-	184	11.08
Cu$_1$@mpgCN$_x$	31.53	51.68	1.85	0.61	2.55	240	14.85

[a]C, N, and H content from combustion analysis; [b]ICP-OES data; [c]BET specific surface area by N_2 isotherms collected at 77 K, in the $0.05 < p/p_0 < 0.3$ range. [d]BJH pore diameter.

Table S2. *Structural information derived from EXAFS analysis of reference materials and Cu$_1$@mpgCN$_x$.*

**Sample**	**Scattering path**	***N*[a]**	***R*[b] (Å)**	**σ^2[c] (10^{-3}Å^2)**	**ΔE_0[d] (eV)**	***R*-factor[e]**
Cu foil	Cu-Cu	12	2.540±0.004	8.30±0.62	4.31±0.68	0.001
CuO	Cu-O	4	1.956±0.012	4.87±0.94	3.16±0.92	0.017
	Cu-Cu(1)	4	2.896±0.017	7.51±1.64		
	Cu-Cu(2)	6	3.106±0.038	11.4±2.65		
Cu_2O	Cu-O	2	1.826±0.008	4.58±0.66	4.37±0.82	0.012
	Cu-Cu	12	3.041±0.009	20.0±0.77		
Cu$_1$@mpgCN$_x$	Cu-N	4.0±0.3	1.938±0.006	7.00±0.90	5.00±0.65	0.006
	Cu-C	2.2±0.4	2.195±0.036	16.6±1.36		

[a]Coordination number. [b]Interatomic distance. [c]Debye-Waller factor. [d]Edge-energy shift. [e]The fit was carried out in R-space, and the R-factor is the measure of the goodness of fit (the higher the R-factor, the lower the misfit is).

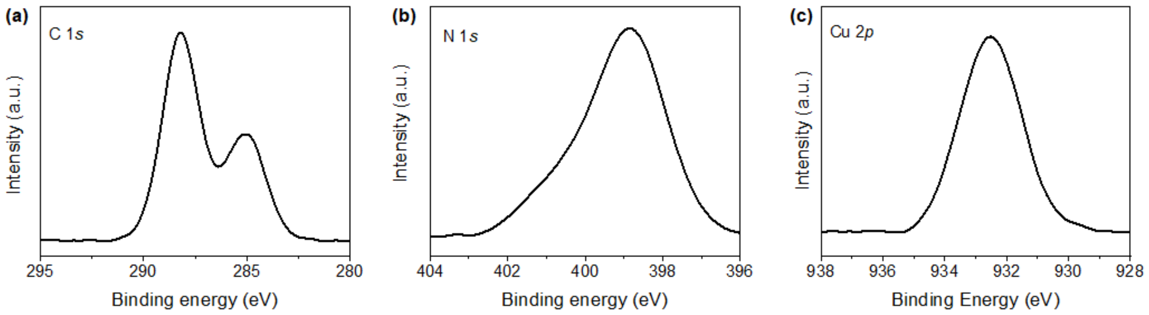

Figure S1. *(a) C 1s, (b) N 1s, and (c) Cu 2p XPS spectra of Cu$_1$@mpgCN$_x$*

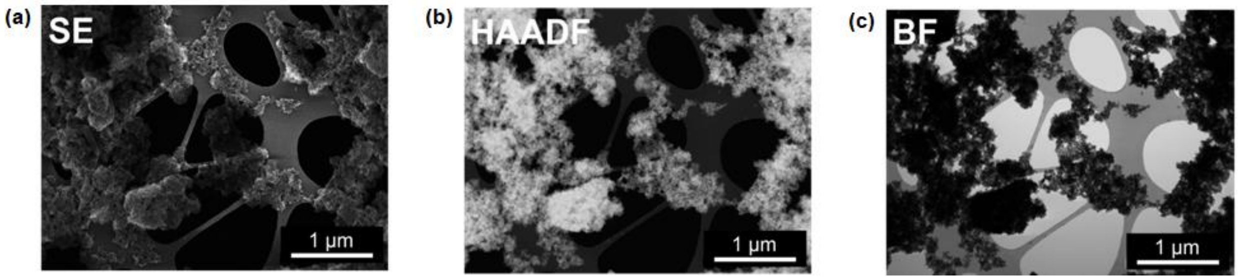

Figure S2. *(a) Secondary electron (SE), (b) HAADF, and (c) bright field (BF) images of* Cu_1*@mpgCN*$_x$*.*

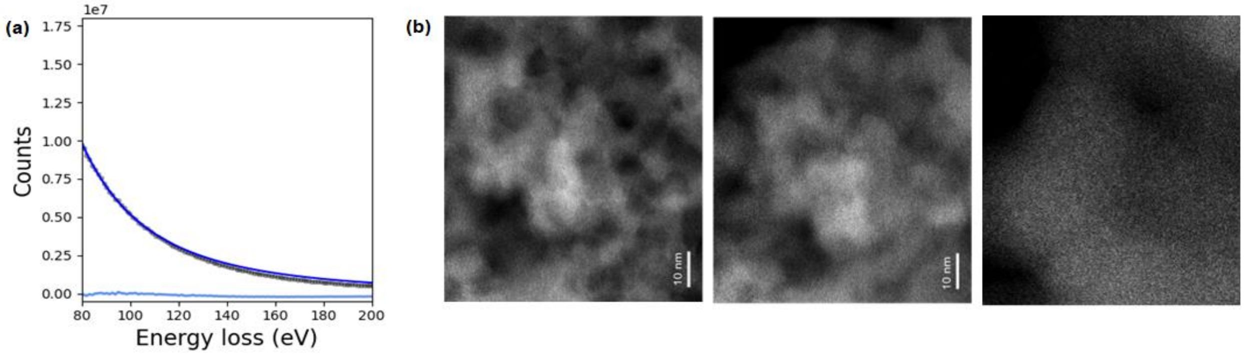

Figure S3*. (a) EEL spectrum corresponding to Figure 3 displayed over the energy loss window of the Si* L_{23} *edge. The as-acquired data is shown as gray circles, with a power-law background fit over the pre-edge region plotted as a solid blue line, resulting in a reducing quality of fit at higher energy losses beyond the onset at 99 eV. (b) Three additional fields of view used for EELS acquisition and summation for retrieving the Cu signal presented in Figure 3. None of the four fields of view examined (Figure 3 and the three shown here) exhibit any nanoparticles or aggregates.*

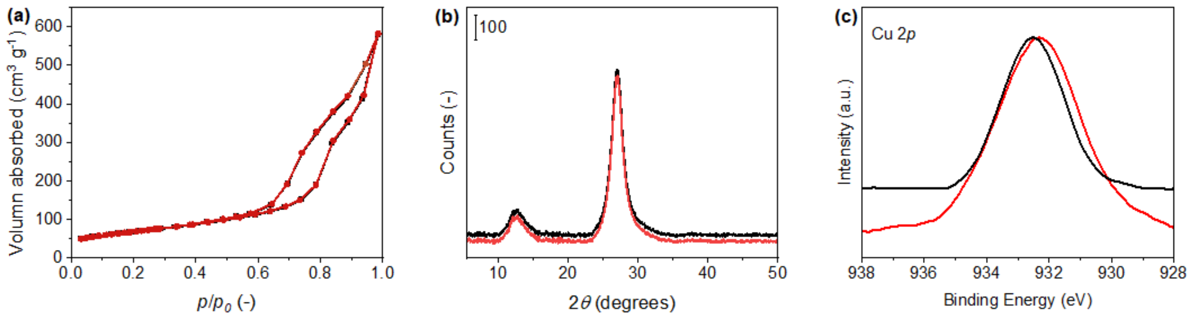

Figure S4. *(a) N_2 physisorption isotherms, (b) XRD patterns, and (c) Cu 2p XPS spectra of Cu_1@mpgCN_x before (black) and after (red) catalytic tests at optimized conditions.*

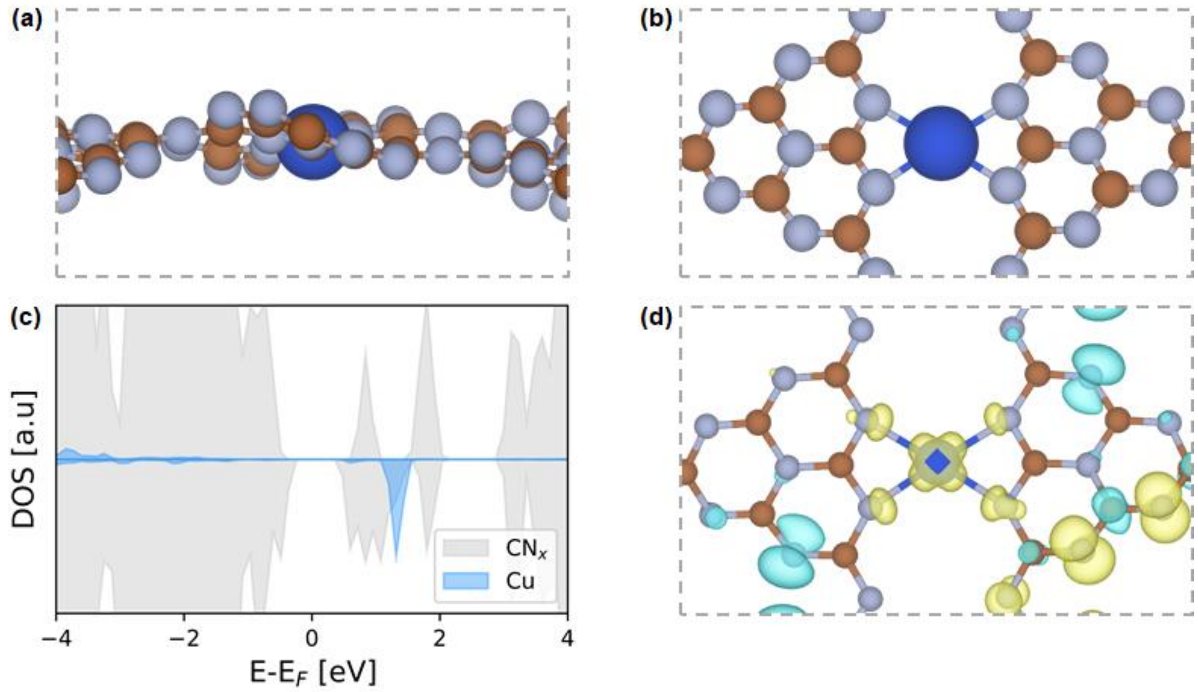

Figure S5. *(a) Side and (b) top view of a Cu single atom anchored within the cavity of the support framework in Cu_1@mpgCN_x. (c) Density of states for the Cu_1@mpgCN_x catalyst. (d) Isosurface distribution of Cu_1@mpgCN_x; the spin density isosurface was set at 0.005 e/A^3. Color code: light purple = N; brown = C; blue = Cu.*

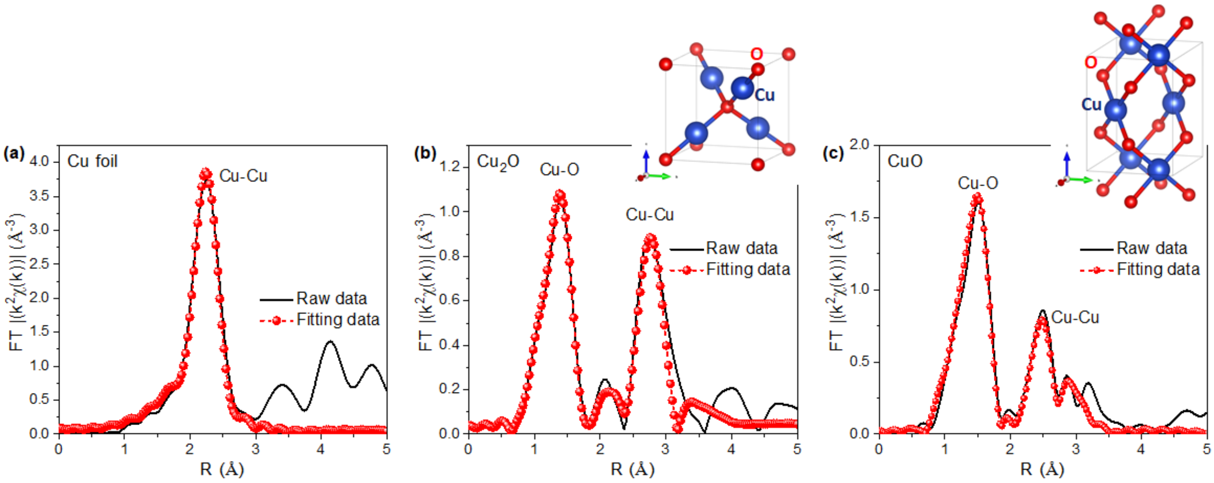

Figure S6. *EXAFS fitting curves of reference samples. (a) Cu foil, (b) Cu_2O, and (c) CuO.*

NMR characterization of the compounds in Figure 4a

Benzyl phenyl sulfide (1a): Colorless oil, 90 % yield.

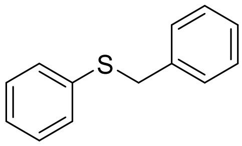

^{1}H NMR (400 MHz, CDCl$_3$): δ 7.40–7.21 (m, 10H), 4.19 (s, 2H). **^{13}C{^{1}H} NMR (101 MHz, CDCl$_3$):** δ 137.6, 136.5, 129.9, 128.9, 128.6, 126.4, 39.1. The spectroscopic data agrees with established values in the literature[1].

4-Fluorophenyl octyl sulfide (1b): Colorless oil, 90 % yield.

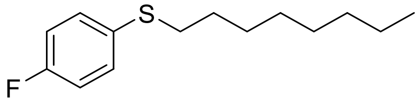

^{1}H NMR (400 MHz, CDCl$_3$): δ 7.34–7.31 (m, 2H), 7.01–6.95 (m, 2H), 2.85 (t, 2H), 1.60 (q, 2H), 1.40 (q, 2H), 1.29–1.25 (m, 8H), 0.88 (t, 3H). **^{13}C{^{1}H} NMR (101 MHz, CDCl$_3$):** δ 163.0, 160.6, 132.2, 131.9, 116.2, 115.9, 35.2, 31.9, 29.3, 29.3, 29.3, 28.9, 22.8, 14.2. **^{19}F{^{1}H} NMR (376 MHz, CDCl$_3$):** δ -116.23 (s, 1F). The spectroscopic data agrees with established values in the literature.[2]

4-Methylphenyl octyl sulfide (1c): Colorless oil, 92 % yield.

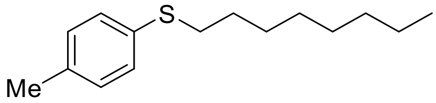

^{1}H NMR (400 MHz, CDCl$_3$): δ 7.25–7.23 (m, 2H), 7.11–7.07 (m, 2H), 2.87 (t, 2H), 2.32 (s, 1H), 1.62 (q, 2H), 1.40 (q, 2H), 1.30–1.24 (m, 8H), 0.88 (t, 3H). **^{13}C{^{1}H} NMR (101 MHz, CDCl$_3$):** δ 134.0, 133.3, 131.3, 129.8, 34.5, 32.0, 29.4, 29.3, 29.0, 29.2, 22.8, 21.1, 14.2. The spectroscopic data agrees with established values in the literature.[2]

4-Fluorophenyl dodecyl sulfide (1d): Colorless oil, 89 % yield.

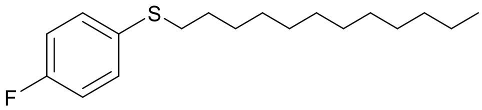

^{1}H NMR (400 MHz, CDCl$_3$): δ 7.35 –7.30 (m, 2H), 7.01–6.95 (m, 2H), 2.85 (t, 2H), 1.64–1.56 (m, 2H), 1.42–1.25 (m, 18H), 0.88 (t, 3H). **^{13}C{^{1}H} NMR (101 MHz, CDCl$_3$):** δ 163.0, 160.6, 132.1, 132.0, 131.9, 116.2,

115.9, 35.2, 32.1, 29.9, 29.8, 29.6, 29.5, 29.3, 29.3, 28.9, 22.8, 14.3. **^{19}F{^{1}H} NMR (376 MHz, $CDCl_3$):** *δ* -116.23 (s, 1F). The spectroscopic data agrees with established values in the literature.[3]

Dodecyl(p-tolyl)sulfane (1e): Colorless oil, 90 % yield

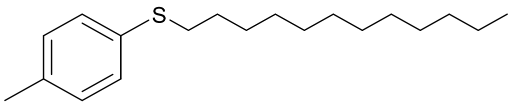

^{1}H NMR (400 MHz, $CDCl_3$): *δ* 7.24 (d, 2H), 7.09 (d, 2H), 2.87 (t, 2H), 2.42 (s, 1H), 2.31 (s, 2H), 1.69–1.58 (m, 2H), 1.42–1.25 (m, 18H), 0.88 (t, 3H). **^{13}C{^{1}H} NMR (101 MHz, $CDCl_3$):** *δ* 135.8, 133.2, 129.8, 129.6, 34.4, 31.9, 29.7, 29.6, 29.6, 29.5, 29.4, 29.2, 28.8, 22.7, 21.0, 14.1. The spectroscopic data agrees with established values in the literature.[3]

Cyclohexyl(phenyl)sulfane (1f): Colorless oil, 85 % yield

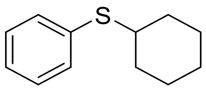

^{1}H NMR (400 MHz, $CDCl_3$): *δ* 7.41–7.38 (m, 2H), 7.30–7.27 (m, 2H), 7.23–7.19 (m, 1H), 3.13–3.07 (m, 1H), 2.00–1.94 (m, 2H), 1.79–1.76 (m, 2H), 1.63–1.59 (m, 1H), 1.42–1.23 (m, 5H). **^{13}C{^{1}H} NMR (101 MHz, $CDCl_3$):** *δ* 135.3, 132.0, 128.9, 126.7, 46.7, 33.5, 26.2, 25.9. The spectroscopic data agrees with established values in the literature.[4]

Cyclohexyl(4-fluorophenyl)sulfane (1g): Colorless oil, 85 % yield

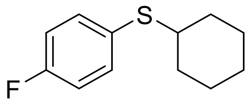

^{1}H NMR (400 MHz, $CDCl_3$): *δ* 7.42–7.37 (m, 2H), 7.01–6.95 (m, 2H), 3.01–2.94 (m, 1H), 1.96–1.92 (m, 2H), 1.78–1.74 (m, 2H), 1.62–1.59 (m, 1H), 1.37–1.26 (m, 5H). **^{13}C{^{1}H} NMR (101 MHz, $CDCl_3$):** *δ* 162.4 (d), 135.2 (d), 130.0 (d), 115.9 (d), 47.8, 33.5, 26.2, 25.9. **^{19}F{^{1}H} NMR (376 MHz, $CDCl_3$):** *δ* -114.92 (s, 1F). The spectroscopic data agrees with established values in the literature.[4]

Cyclohexyl(4-methoxyphenyl) sulfane (1h): Colorless oil, 86 % yield.

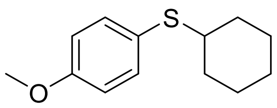

^{1}H NMR (400 MHz, $CDCl_3$): δ 7.40–7.36 (m, 2H), 6.85–6.82 (m, 2H), 3.80 (s, 3H), 2.93–2.86 (m, 1H), 1.95–1.91 (m, 2H), 1.77–1.73 (m, 2H), 1.61–1.57 (m, 1H), 1.36–1.19 (m, 5H). **$^{13}C\{^{1}H\}$ NMR (101 MHz, $CDCl_3$):** δ 159.5, 135.7, 125.2, 114.4, 55.5, 48.1, 33.5, 26.3, 26.0. The spectroscopic data agrees with established values in the literature.[4]

Cyclohexyl(naphthalen-1-yl) sulfane (1i): Colorless oil, 80 % yield

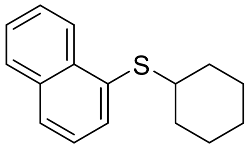

^{1}H NMR (400 MHz, $CDCl_3$): δ 8.55–8.53 (m, 1H), 7.86–7.83 (m, 1H), 7.78 (d, 1H), 7.70–7.68 (m, 1H), 7.58–7.49 (m, 2H), 7.43–7.39 (m, 1H), 3.17–3.10 (m, 1H), 1.99–1.95 (m, 2H), 1.79–1.74 (m, 2H), 1.60–1.58 (m, 1H), 1.49–1.39 (m, 2H), 1.29–1.24 (m, 3H). **$^{13}C\{^{1}H\}$ NMR (101 MHz, $CDCl_3$):** δ 134.6, 134.2, 132.5, 132.0, 128.6, 128.3, 126.5, 126.2, 126.1, 125.6, 47.4, 33.7, 26.2, 26.0. The spectroscopic data agrees with established values in the literature.[5]

2-(cyclohexylthio) pyridine (1i): Colorless oil, 82 % yield.

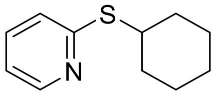

^{1}H NMR (400 MHz, $CDCl_3$): δ 8.44–8.42 (m, 1H), 7.48–7.44 (m, 1H), 7.16–7.14 (m, 1H), 6.98–6.94 (m, 1H), 3.85–3.78 (m, 1H), 2.12–2.04 (m, 2H), 1.79–1.76 (m, 2H), 1.67–1.61 (m, 1H), 1.54–1.40 (m, 4H), 1.37–1.26 (m, 1H). **$^{13}C\{^{1}H\}$ NMR (101 MHz, $CDCl_3$):** δ 159.5, 149.7, 136.0, 123.0, 119.4, 43.1, 33.4, 26.2, 26.0. The spectroscopic data agrees with established values in the literature.[6]

Diphenylsulphane (1k): Colorless oil, 86 % yield.

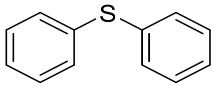

^{1}**H NMR (400 MHz, CDCl$_3$):** δ 7.46 – 7.29 (m, 10H). **^{13}C{^{1}H} NMR (101 MHz, CDCl$_3$):** δ 135.9, 131.0, 129.2, 127.1. The spectroscopic data agrees with established values in the literature.[1]

4-Methoxyphenyl) (phenyl)sulfane (1l): Colorless oil, 90 % yield.

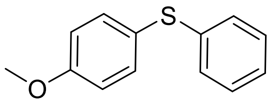

^{1}**H NMR (400 MHz, CDCl$_3$):** δ 7.46 (d, 2H), 7.30–7.16 (m, 5H), 6.94 (d, 2H), 3.87 (s, 3H). **^{13}C{^{1}H} NMR (101 MHz, CDCl$_3$):** δ 160.0, 138.7, 135.5, 129.1, 128.4, 125.9, 124.5, 115.1, 55.5. The spectroscopic data agrees with established values in the literature.[7]

Naphthalen-1-yl(phenyl)sulfane (1m): Colorless oil, 89 % yield.

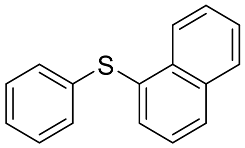

^{1}**H NMR (400 MHz, CDCl$_3$):** δ 8.41–8.37 (m, 1H), 7.90–7.86 (m, 2H), 7.67 (dd, 1H), 7.53 (dt, 2H), 7.44 (dd, 1H), 7.25–7.14 (m, 5H). **^{13}C{^{1}H} NMR (101 MHz, CDCl$_3$):** δ 137.1, 134.4, 133.8, 132.7, 131.4, 129.3, 129.2, 129.1, 128.7, 127.1, 126.6, 126.3, 126.0, 125.8. The spectroscopic data agrees with established values in the literature.[7]

Bis(4-fluorophenyl) sulfide (1n): Colorless oil, 82 % yield.

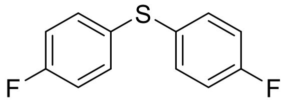

^{1}**H NMR (400 MHz, CDCl$_3$):** δ 7.31 (dt, 4H), 7.01 (t, 4H). **^{13}C{^{1}H} NMR (101 MHz, CDCl$_3$):** δ 163.5, 161.0, 133.1, 133.0, 131.4, 131.3, 116.5, 116.3. **^{19}F{^{1}H} NMR (376 MHz, CDCl$_3$):** δ -114.41, 113.48. The spectroscopic data agrees with literature established values in the literature.[8]

(4-fluorophenyl)(4-methoxyphenyl)sulfane (1o): Colorless oil, 85% yield.

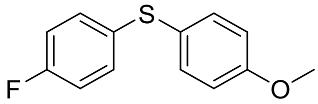

^{1}H NMR (400 MHz, $CDCl_3$): δ 7.36 (d, 2H), 7.23–7.18 (m, 2H), 6.95 (t, 2H), 6.88 (d, 2H), 3.81 (s, 3H). **^{13}C{^{1}H} NMR (101 MHz, $CDCl_3$):** δ 161.7 (d), 159.8, 134.6, 133.3 (d), 131.2 (d), 125.5, 116.2 (d), 115.2, 55.5. **^{19}F{^{1}H} NMR (376 MHz, $CDCl_3$):** δ 116.17. The spectroscopic data agrees with literature established values in the literature.[9]

4-(trifluoromethyl)phenyl phenyl sulfide (1p): Colorless oil, 72% yield).

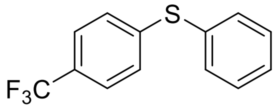

^{1}H NMR (400 MHz, $CDCl_3$): δ 7.49–7.45 (m, 5H), 7.41–7.37 (m, 3H), 7.28 (s, 1H). **^{13}C{^{1}H} NMR (101 MHz, $CDCl_3$):** δ 142.8, 133.5, 132.6, 129.7, 128.3 (q), 127.6, 127.2, 125.8 (q), 125.5. **^{19}F{^{1}H} NMR (376 MHz, $CDCl_3$):** δ 62.51. The spectroscopic data agrees with literature established values in the literature.[10]

4-(Phenylthio)benzonitrile (1q): Colorless oil, 58% yield).

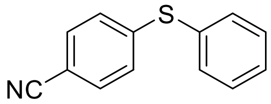

^{1}H NMR (400 MHz, $CDCl_3$): δ 7.52–7.47 (m, 4H), 7.33–7.28 (m, 3H), 7.25–7.21 (m, 2H)). **^{13}C{^{1}H} NMR (101 MHz, $CDCl_3$):** δ 137.2, 133.7, 129.8, 129.2, 128.8, 128.5, 127.7, 127.3, 126.0. The spectroscopic data agrees with literature established values in the literature.[11]

Pharmaceutical derivative

(**2-bromophenyl)(2,4-dimethylphenyl)sulfane (2):** Colorless oil, 80 % yield.

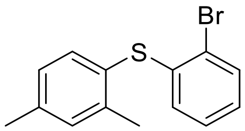

^{1}H NMR (400 MHz, $CDCl_3$): δ 7.52 (dd, 1H), 7.39 (d, 1H), 7.17 (s, 1H), 7.09–7.04 (m, 2H), 6.95 (td, 1H), 6.57 (dd, 1H), 2.36 (s, 3H), 2.33 (s, 3H). **^{13}C{^{1}H} NMR (101 MHz, $CDCl_3$):** δ 142.4, 139.9, 139.5, 136.2,

132.8, 131.9, 128.0, 127.7, 127.4, 127.1, 126.1, 121.2, 21.2, 20.5. The spectroscopic data agrees with established values in the literature.[12]

Radical clock

o-(But-3-enyloxy)phenyl Phenyl Sulfide (4b): Colorless oil, 68% yield.

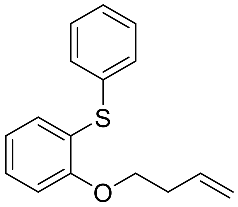

^{1}H NMR (400 MHz, $CDCl_3$): δ 7.72 – 6.63 (m, 8H), 5.98 – 5.88 (m, 1H), 5.19 – 5.07 (m, 2H), 4.01 (t, 2H), 2.56 (q, 2H). **$^{13}C\{^1H\}$ NMR (101 MHz, $CDCl_3$):** δ 157.5, 139.5, 137.1, 134.4, 129.4, 129.1, 127.6, 127.2, 122.5, 117.3, 112.2, 68.7, 33.6. The spectroscopic data agrees with literature established values in the literature.[13]

Benzyl phenyl sulfide

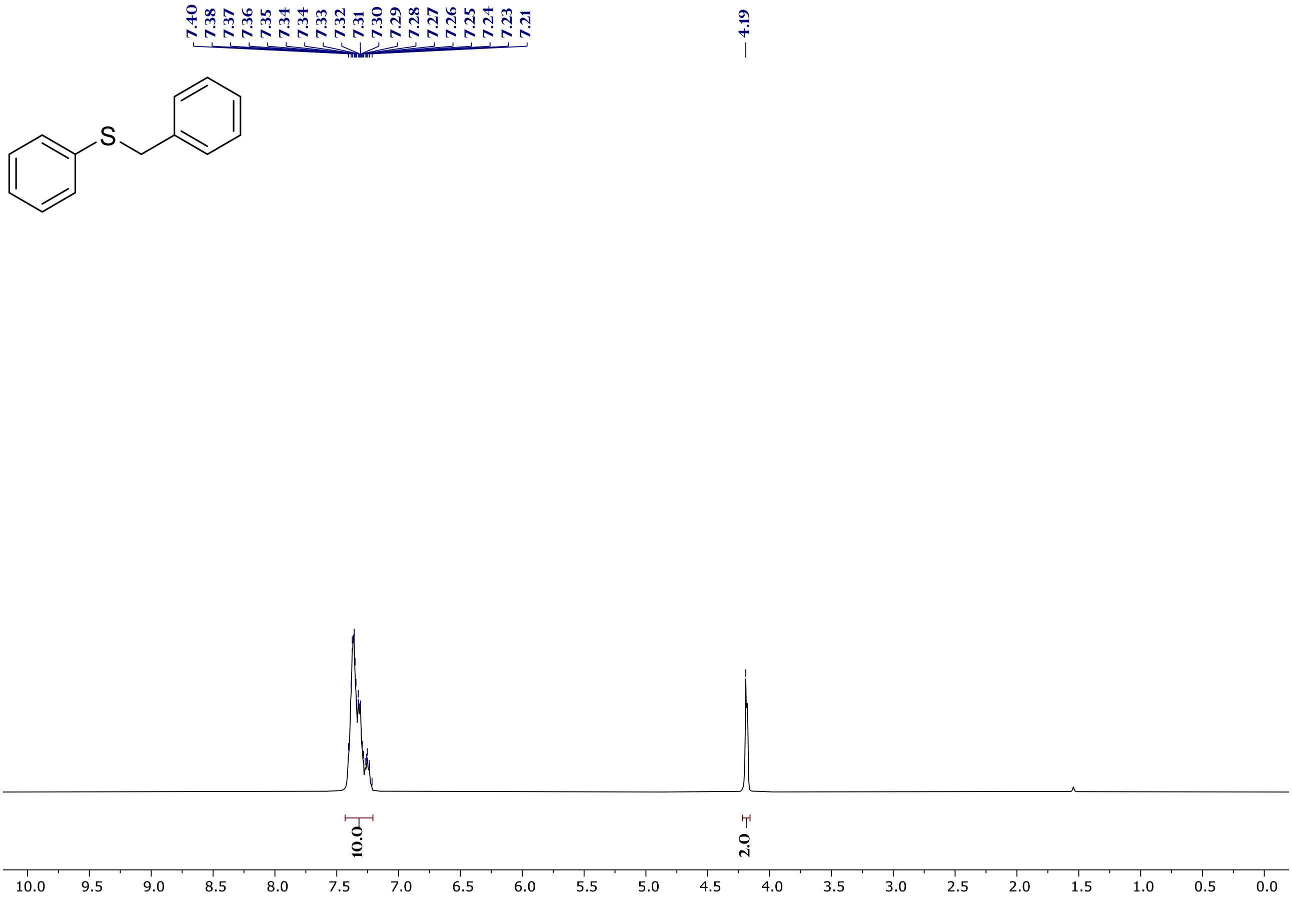

^{1}H-NMR spectrum of benzyl phenyl sulfide.

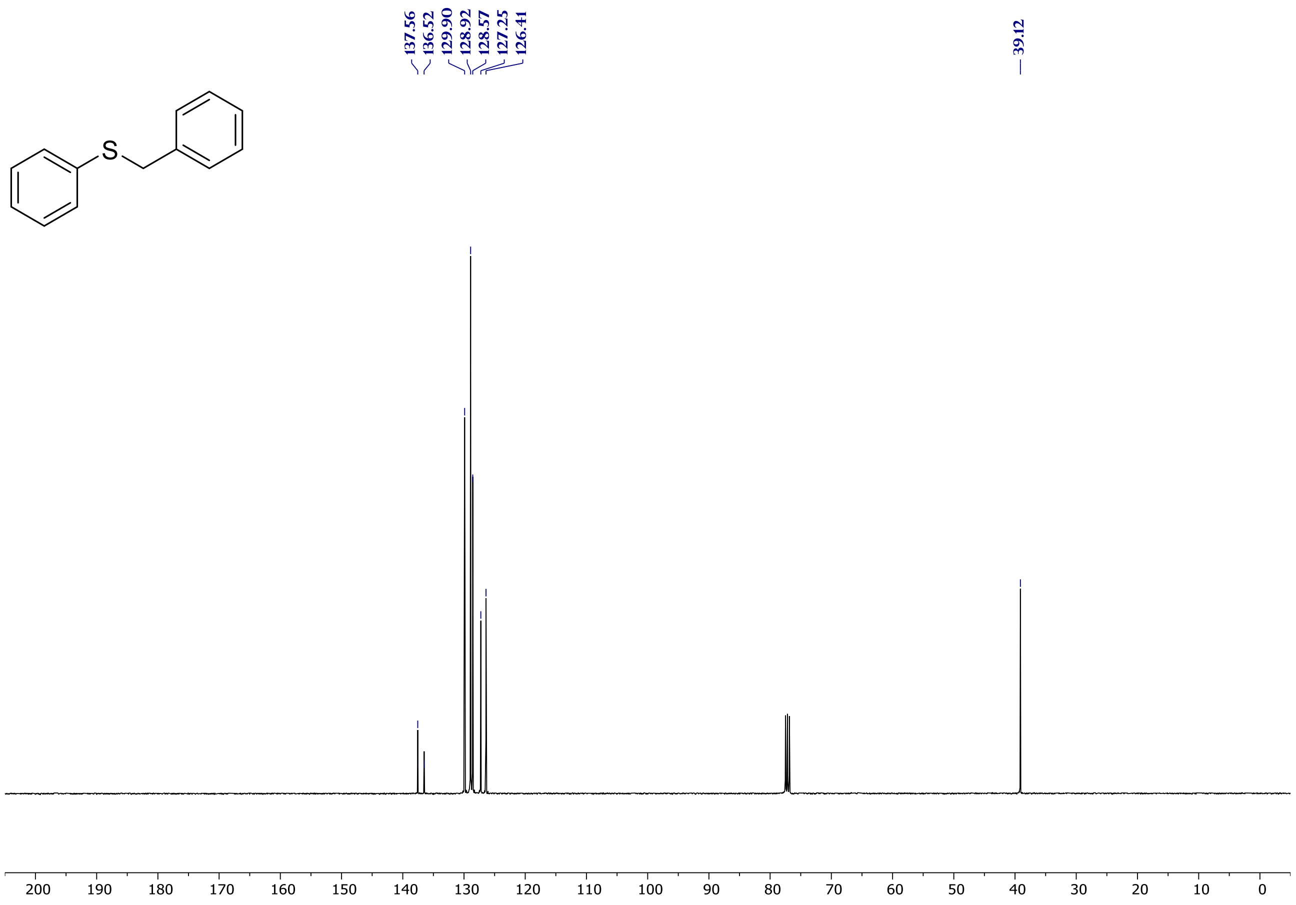

^{13}C-NMR spectrum of benzyl phenyl sulfide.

4-Fluorophenyl octyl sulfide

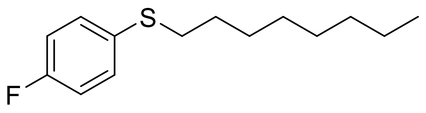

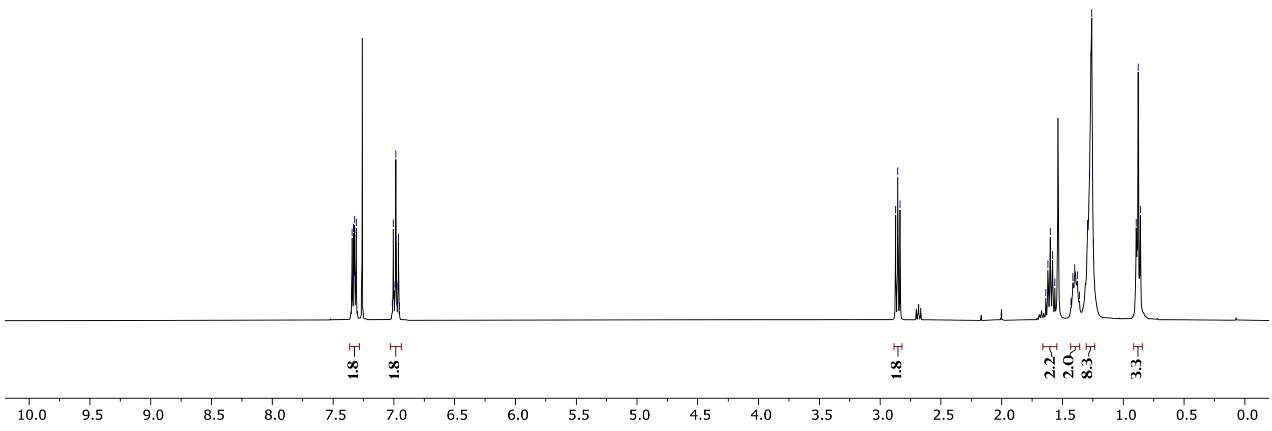

^{1}H-NMR spectrum of 4-fluorophenyl octyl sulfide.

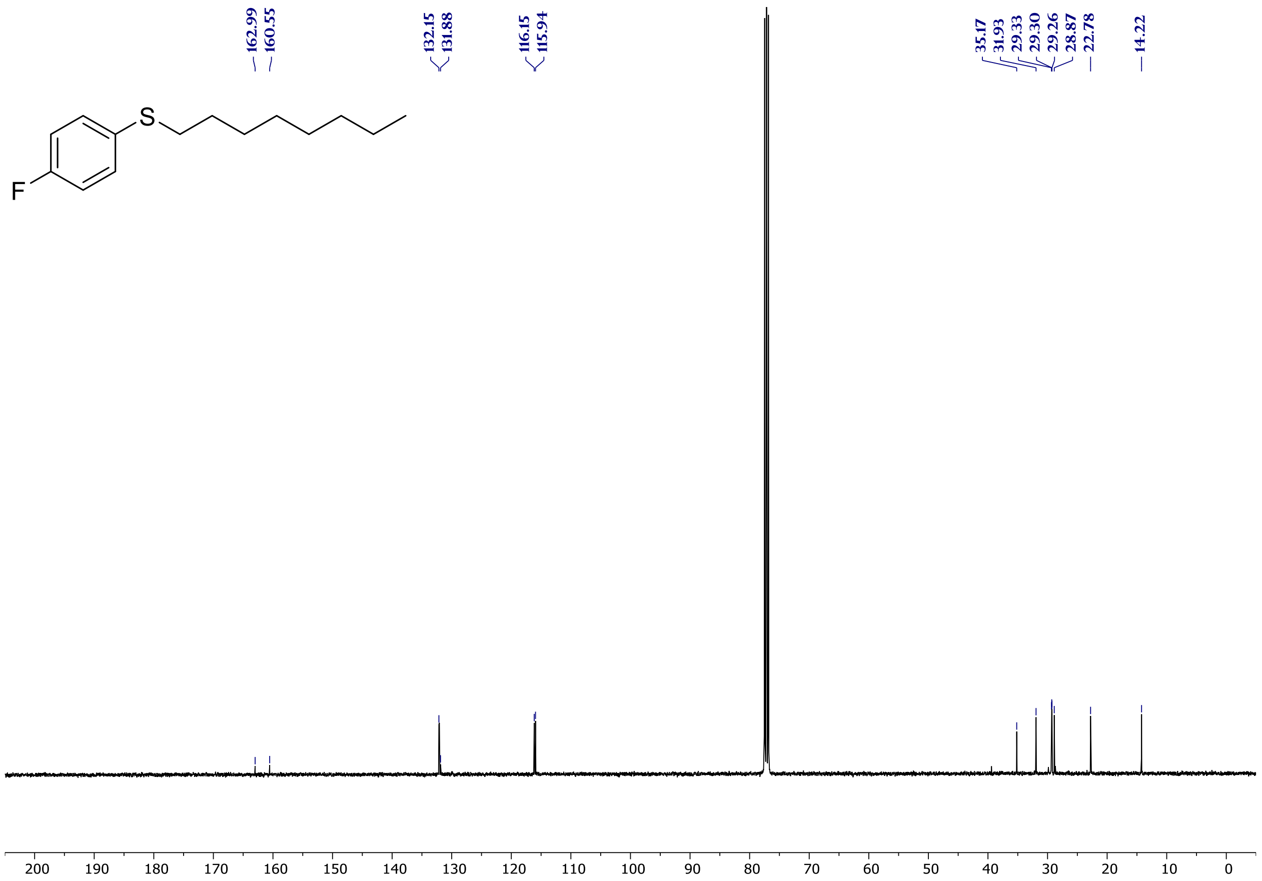

^{13}C-NMR spectrum of 4-fluorophenyl octyl sulfide.

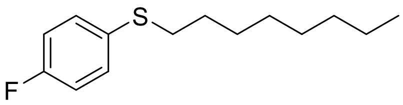

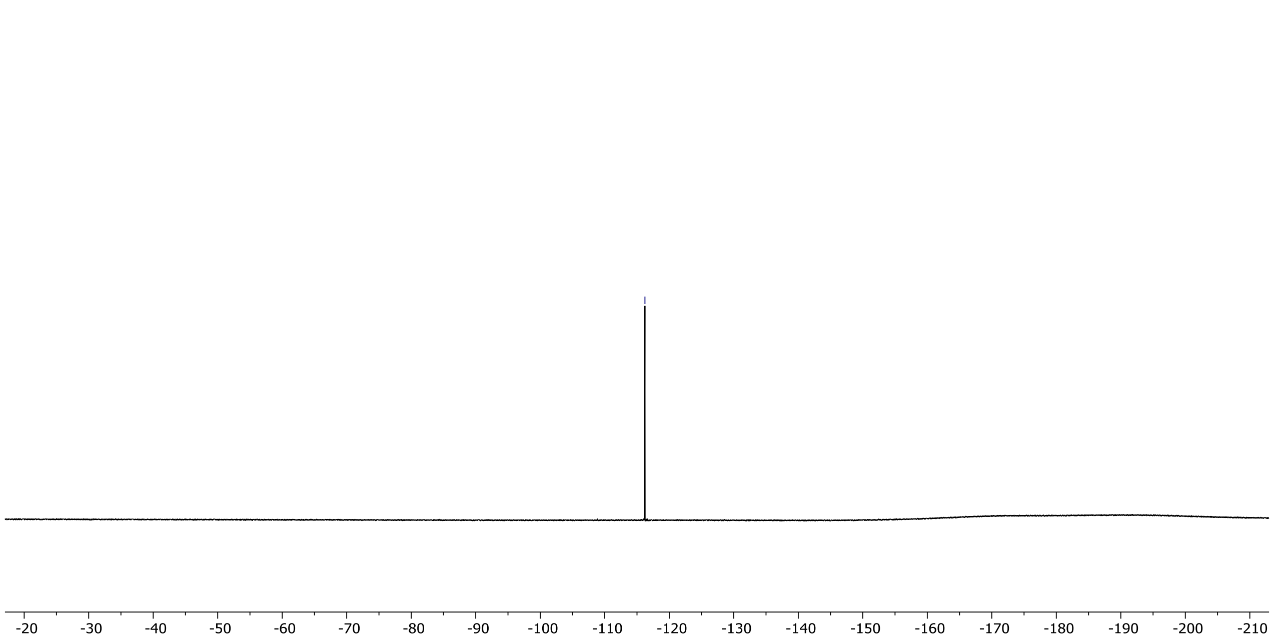

^{19}F-NMR spectrum of 4-fluorophenyl octyl sulfide.

4-Methylphenyl octyl sulfide

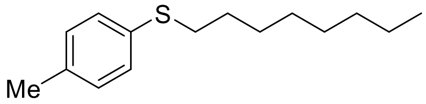

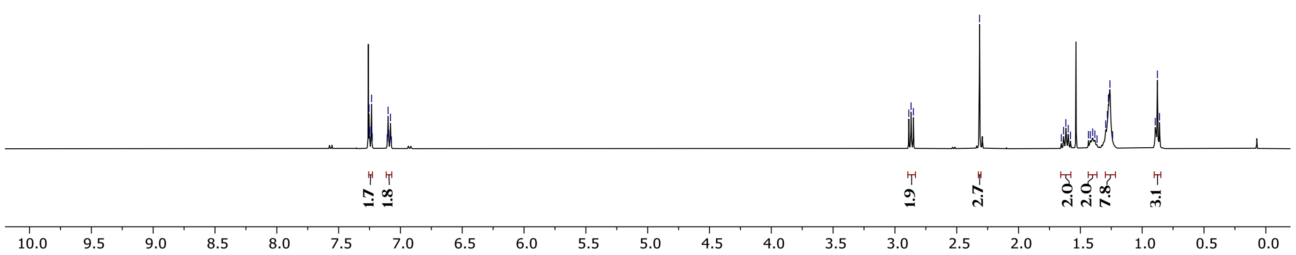

^{1}H-NMR spectrum of 4-methylphenyl octyl sulfide.

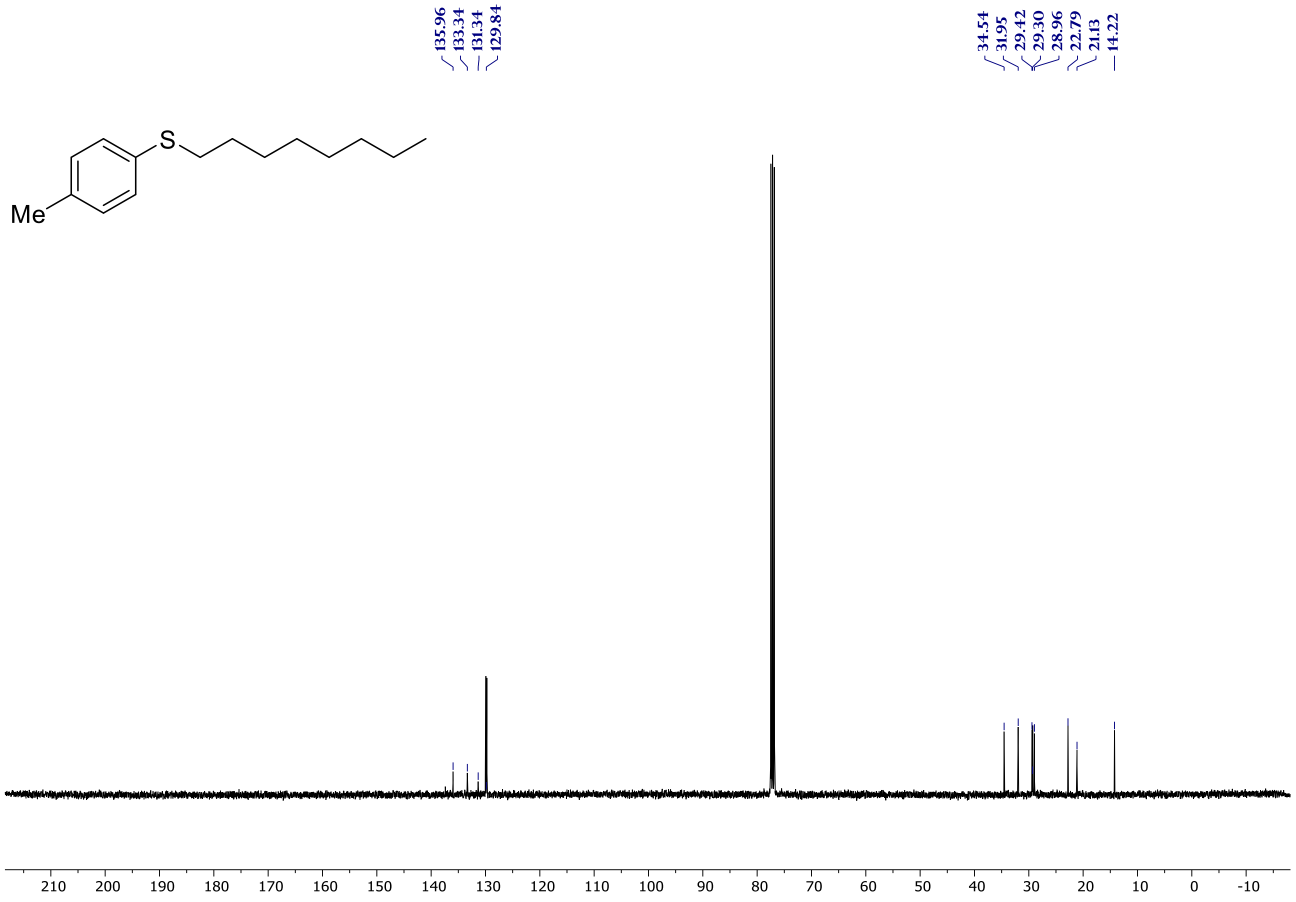

^{13}C-NMR spectrum of 4-methylphenyl octyl sulfide.

4-Fluorophenyl dodecyl sulfide

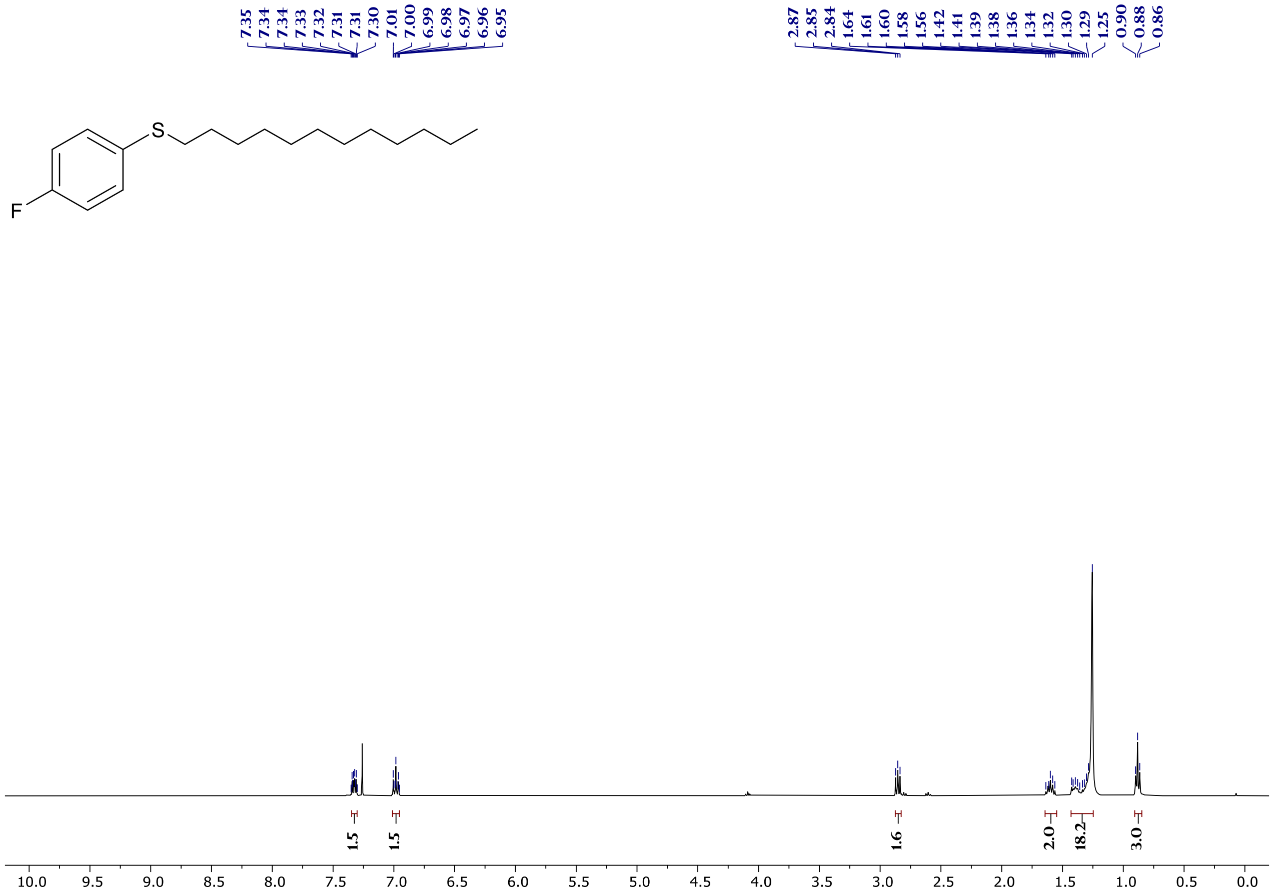

^{1}H-NMR spectrum of 4-fluorophenyl dodecyl sulfide.

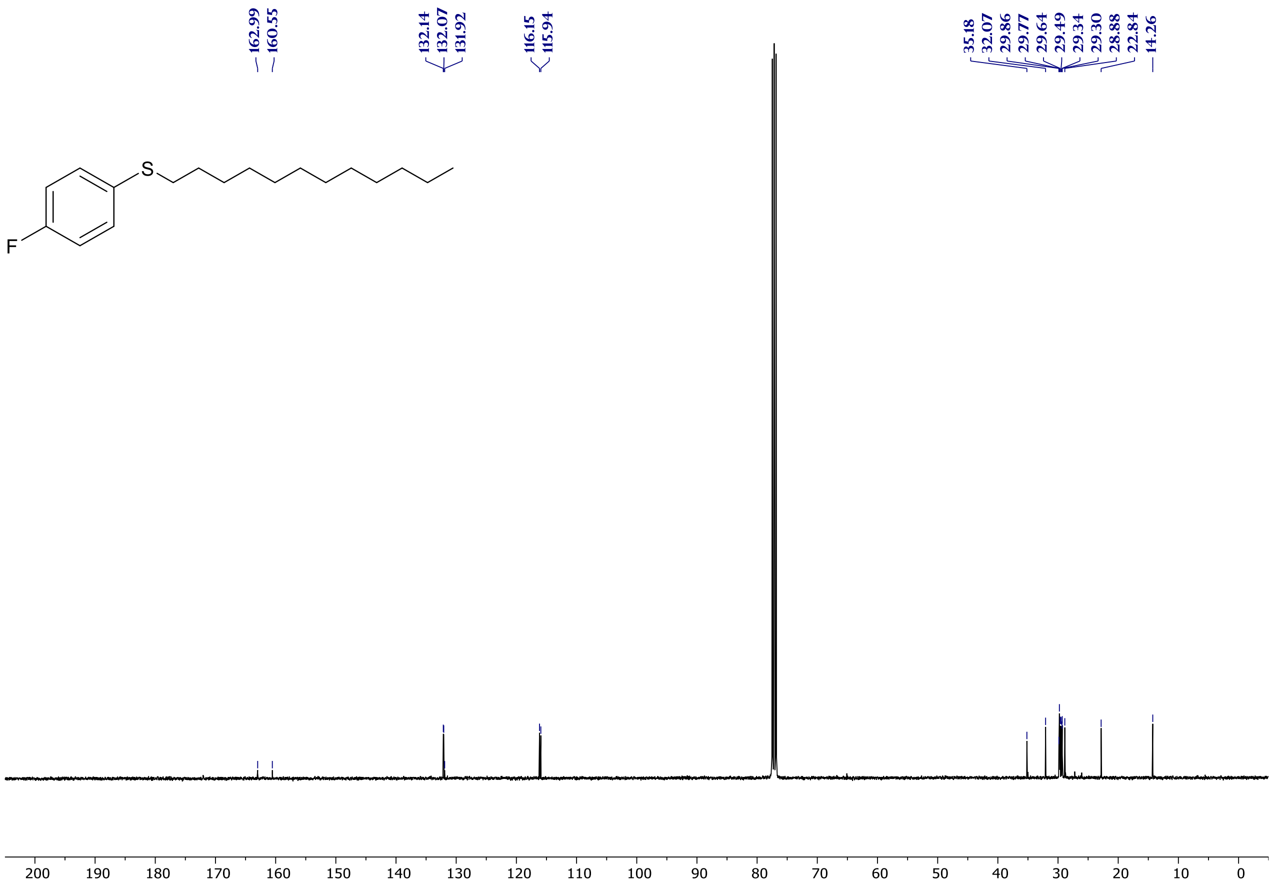

^{13}C-NMR spectrum of 4-fluorophenyl dodecyl sulfide.

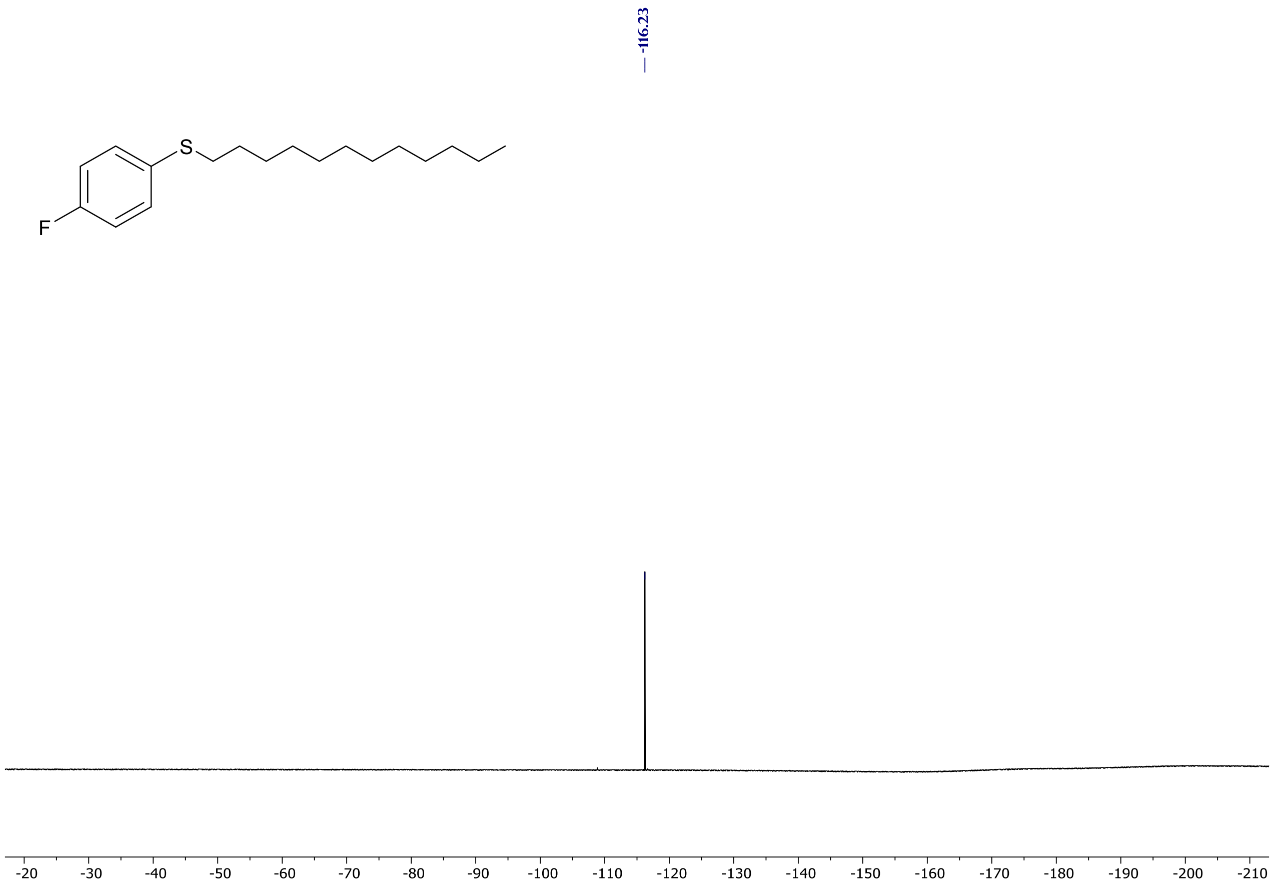

^{19}F-NMR spectrum of 4-fluorophenyl dodecyl sulfide.

Dodecyl(p-tolyl)sulfane

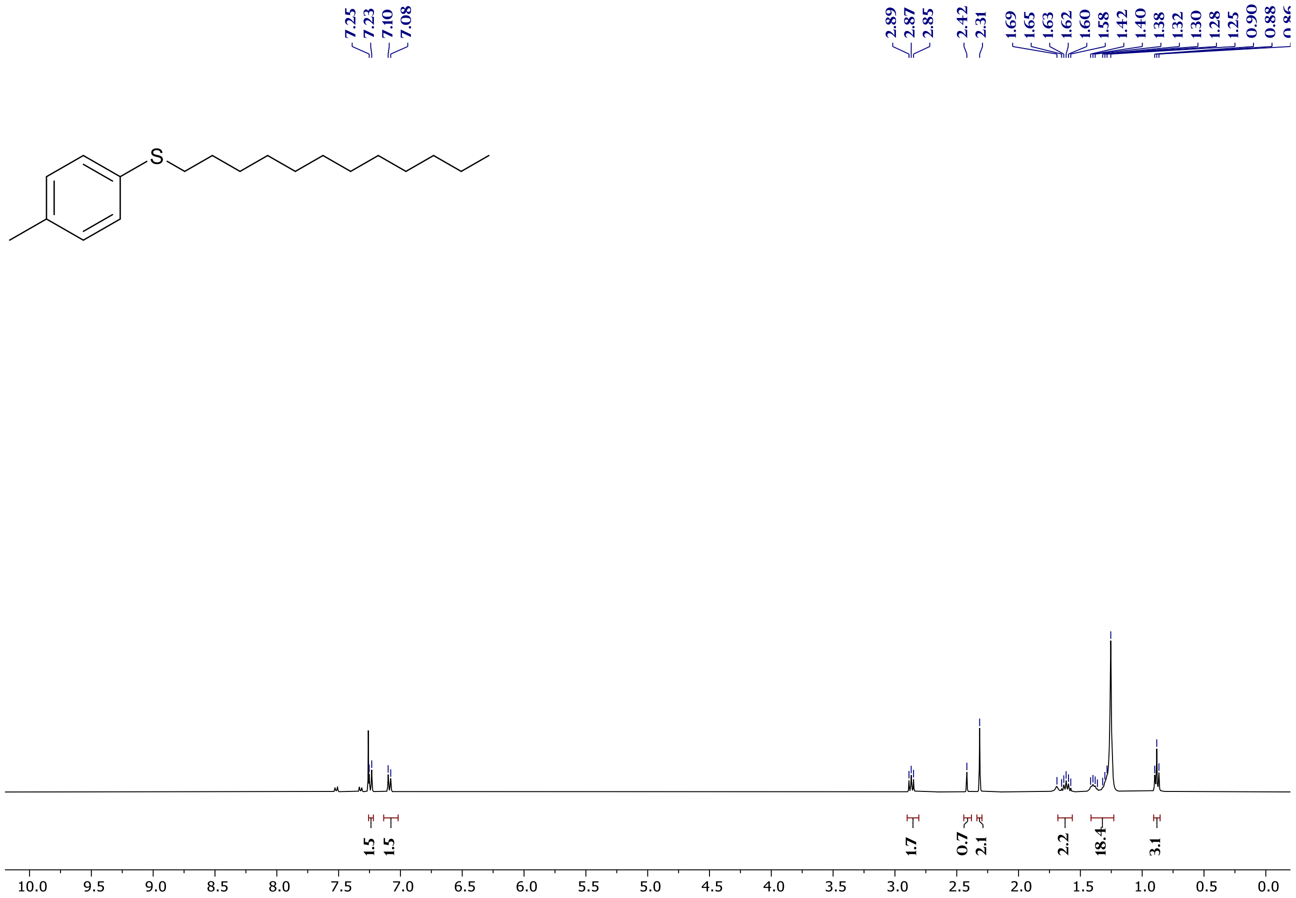

^{1}H-NMR spectrum of dodecyl(p-tolyl)sulfane.

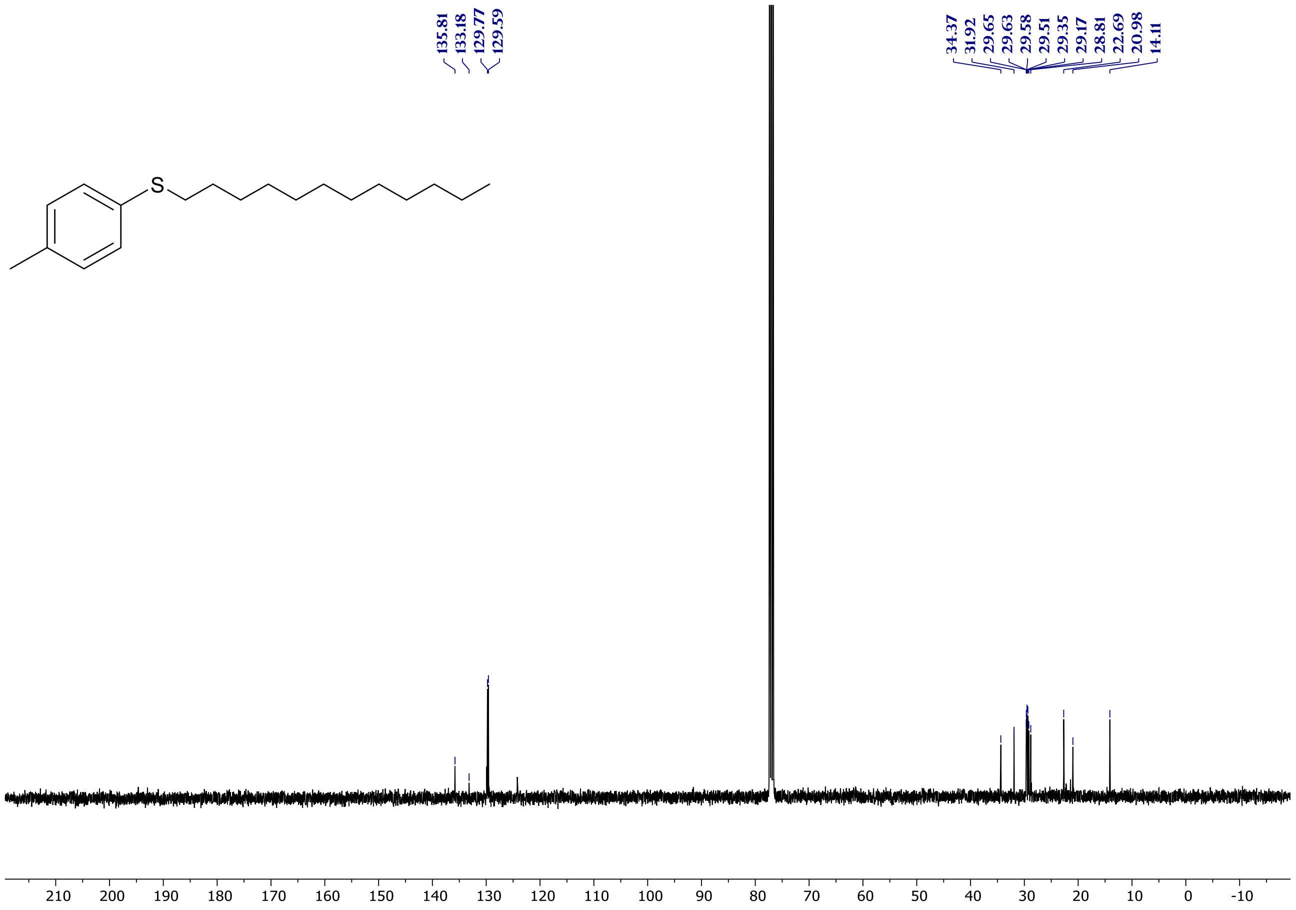

^{13}C-NMR spectrum of dodecyl(p-tolyl)sulfane.

Cyclohexyl(phenyl)sulfane

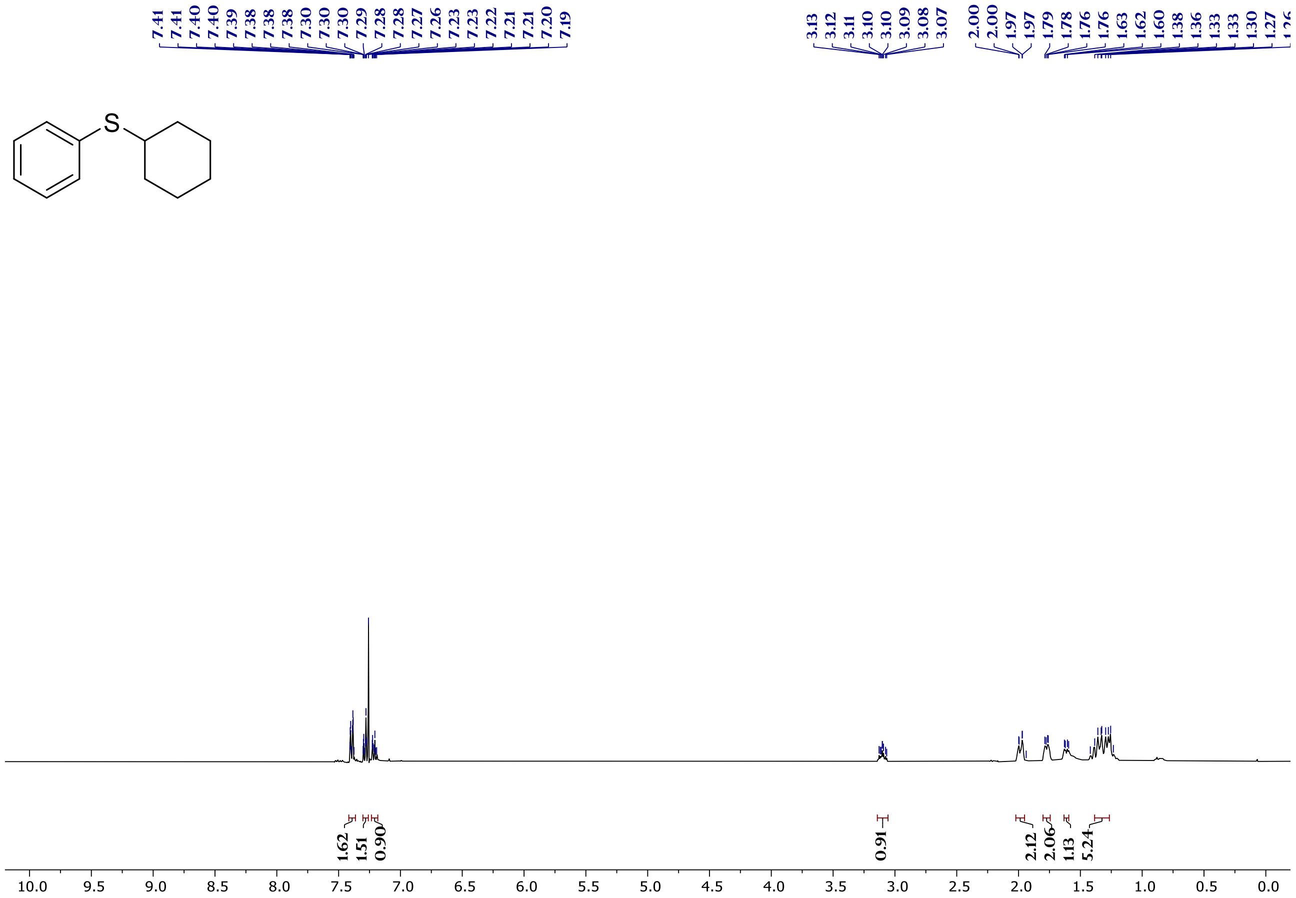

^{1}H-NMR spectrum of cyclohexyl(phenyl)sulfane.

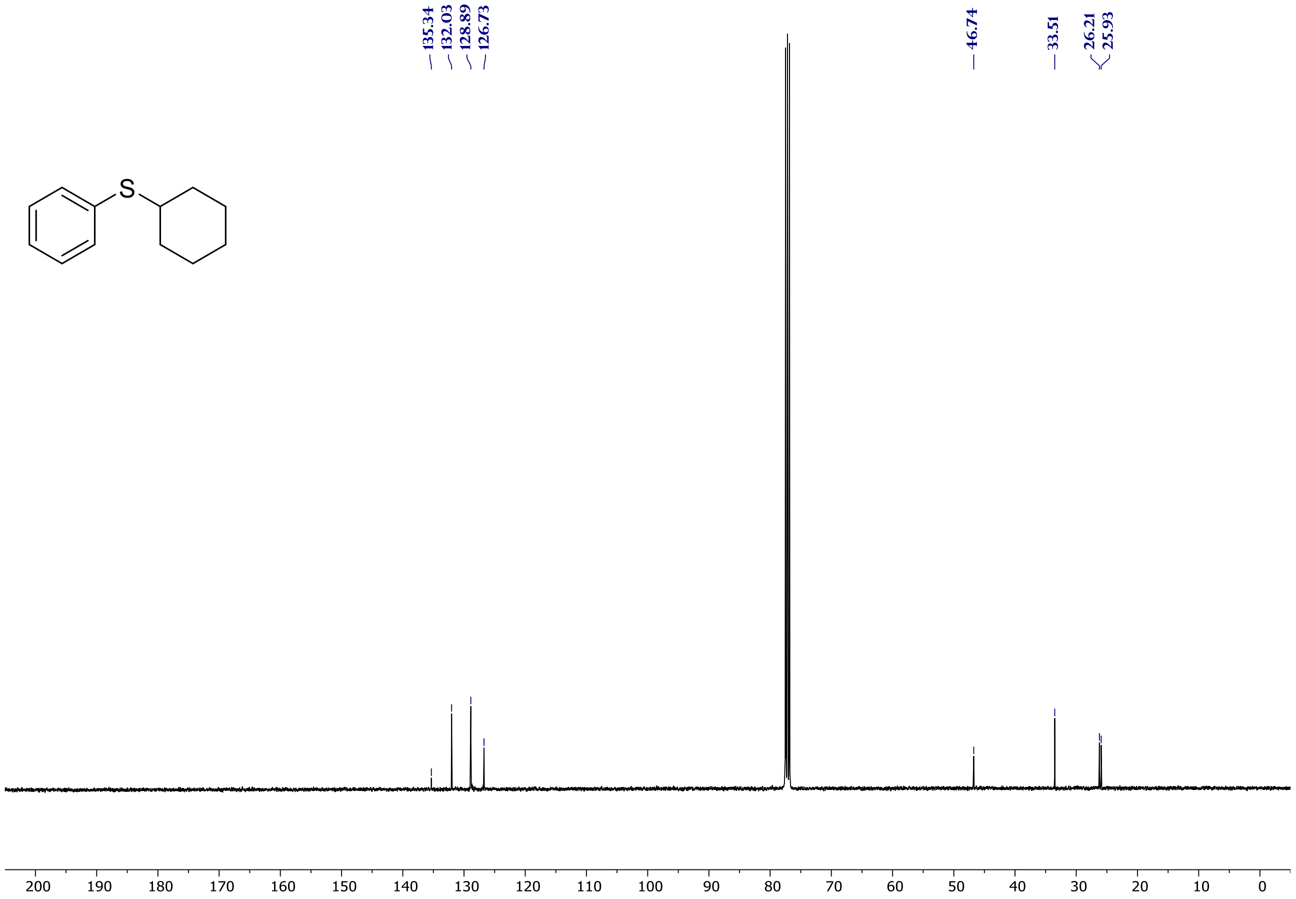

^{13}C-NMR spectrum of cyclohexyl(phenyl)sulfane.

Cyclohexyl(4-fluorophenyl) sulfane

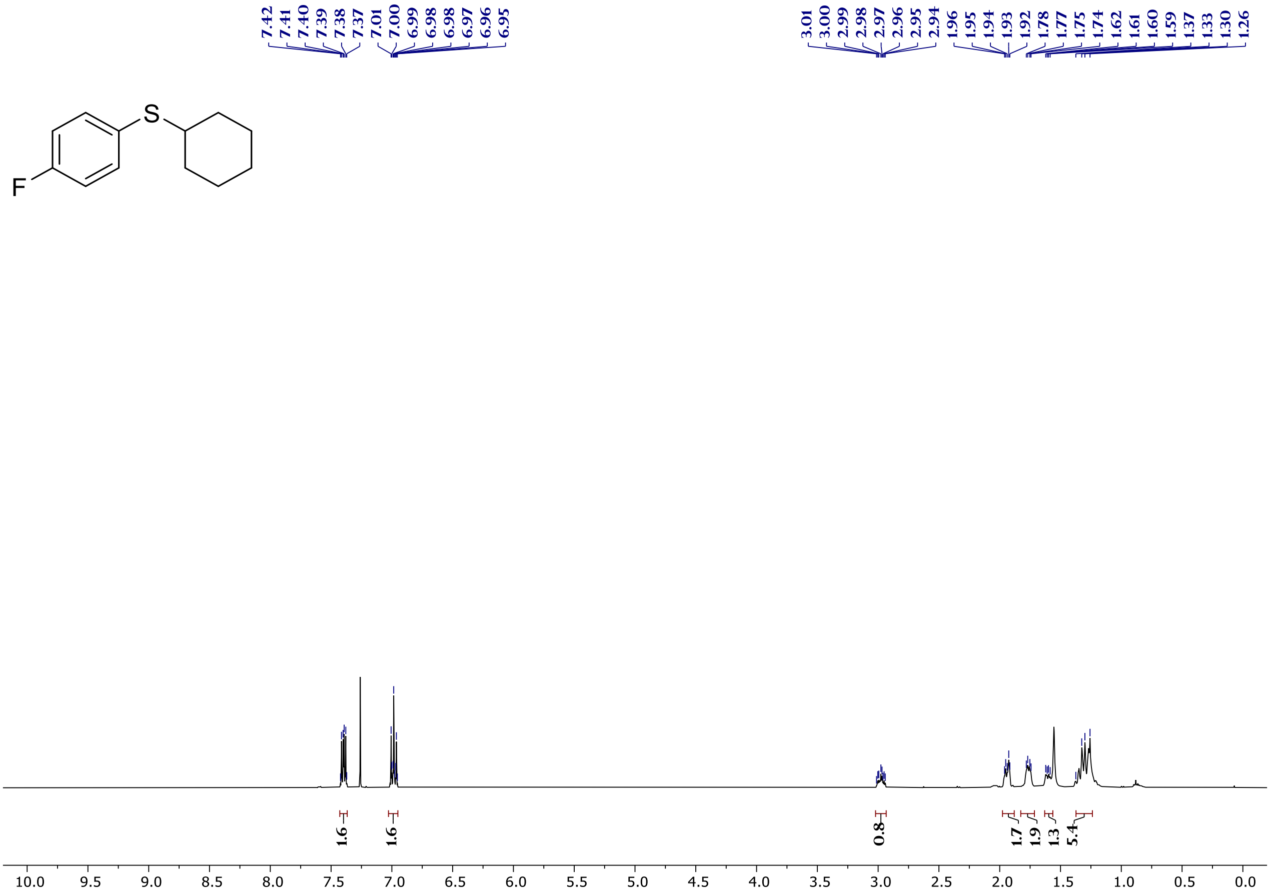

^{1}H-NMR spectrum of cyclohexyl(4-fluorophenyl) sulfane.

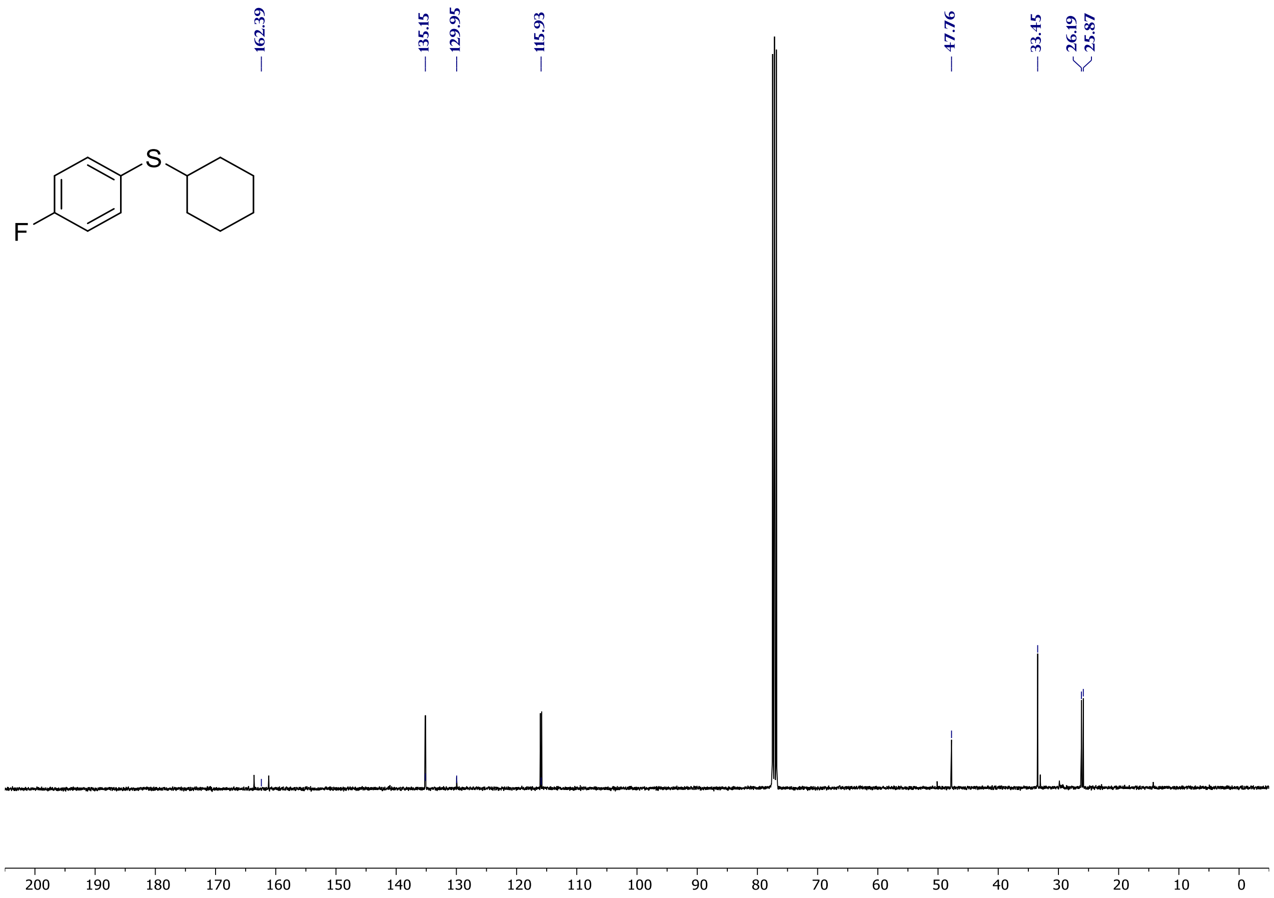

^{13}C-NMR spectrum of cyclohexyl(4-fluorophenyl) sulfane.

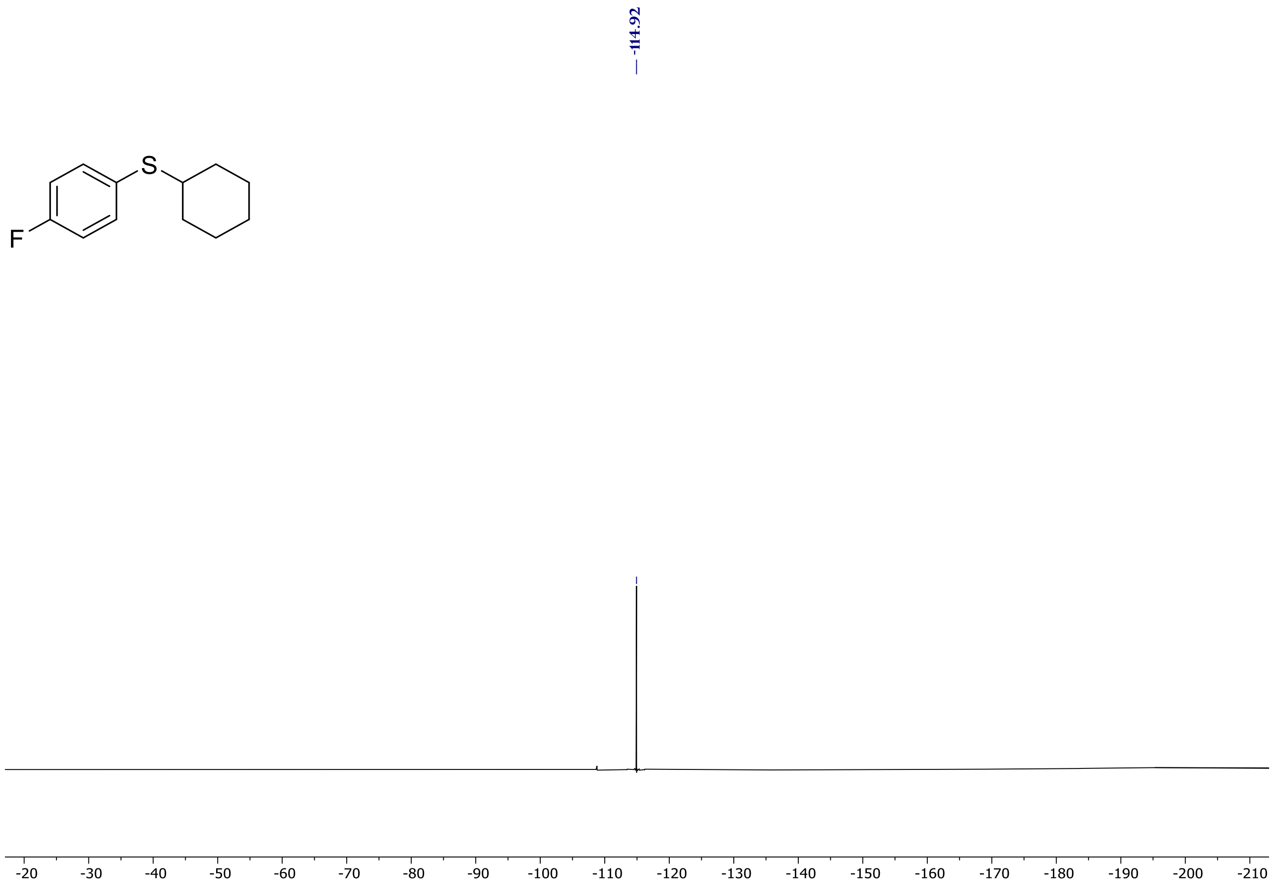

^{19}F-NMR spectrum of cyclohexyl(4-fluorophenyl) sulfane.

Cyclohexyl(4-methoxyphenyl) sulfane

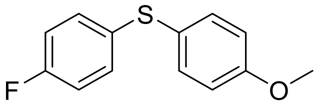

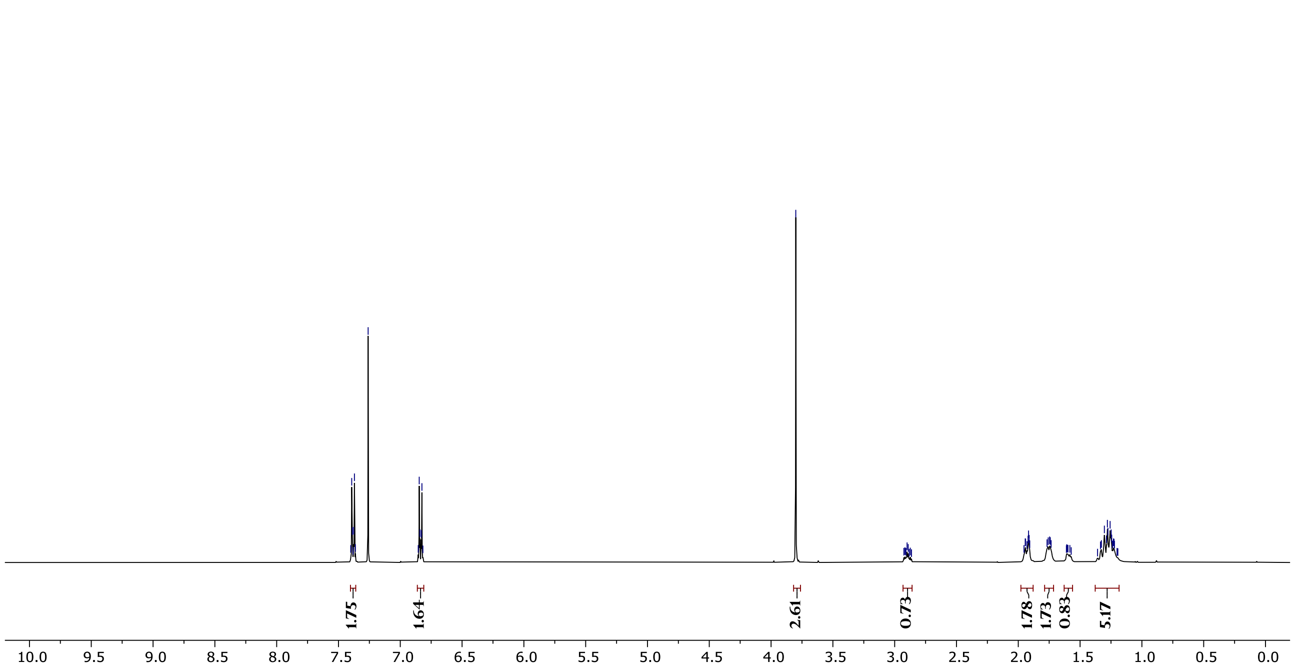

^{1}H-NMR spectrum of cyclohexyl(4-methoxyphenyl) sulfane.

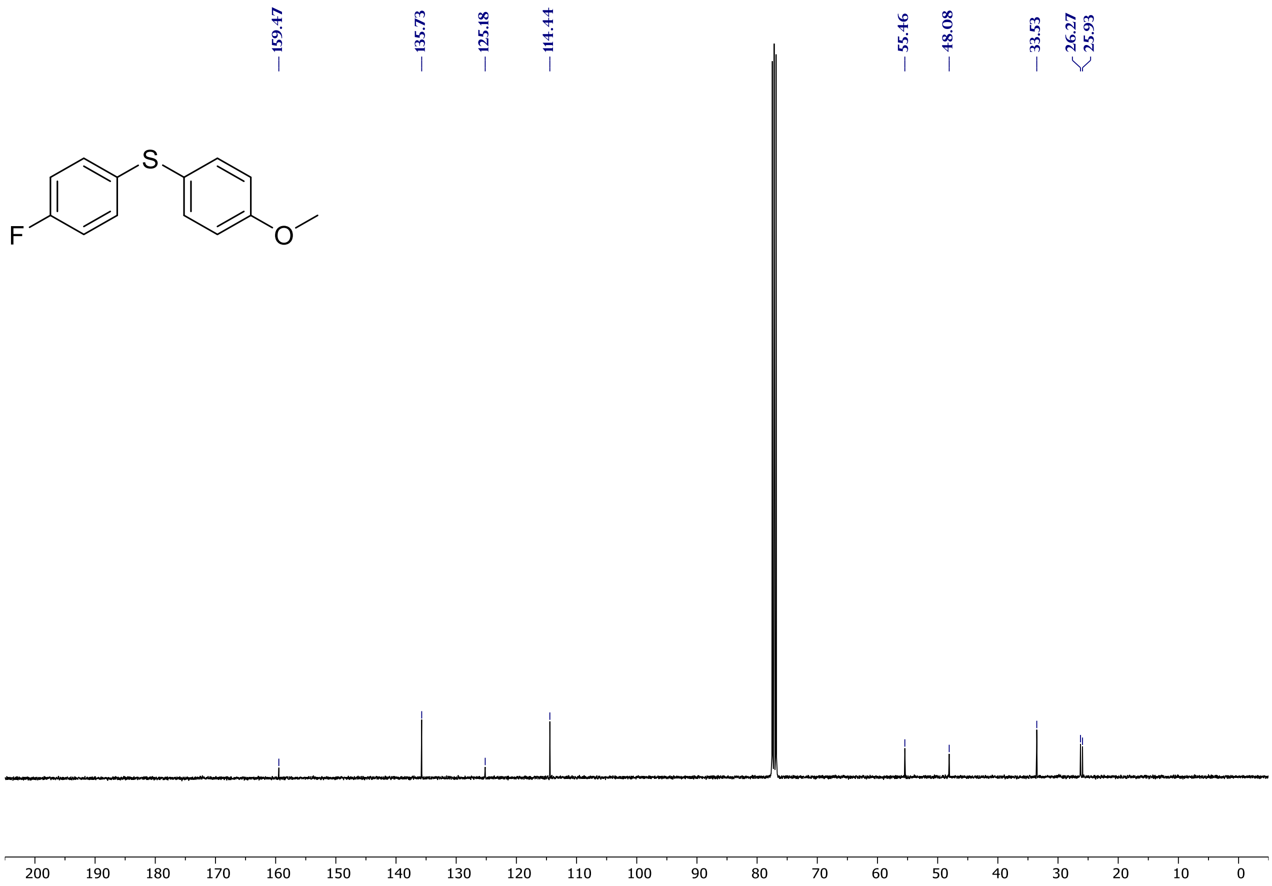

^{13}C-NMR spectrum of cyclohexyl(4-methoxyphenyl) sulfane.

Cyclohexyl(naphthalen-1-yl) sulfane

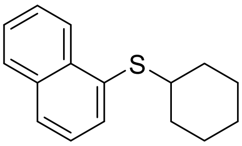

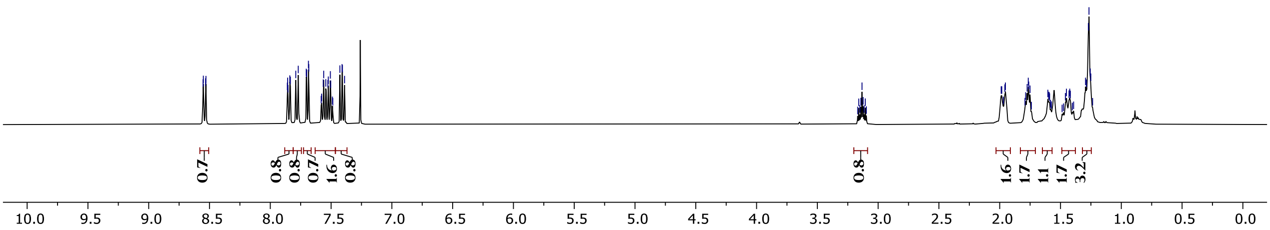

^{1}H-NMR spectrum of cyclohexyl(naphthalen-1-yl) sulfane.

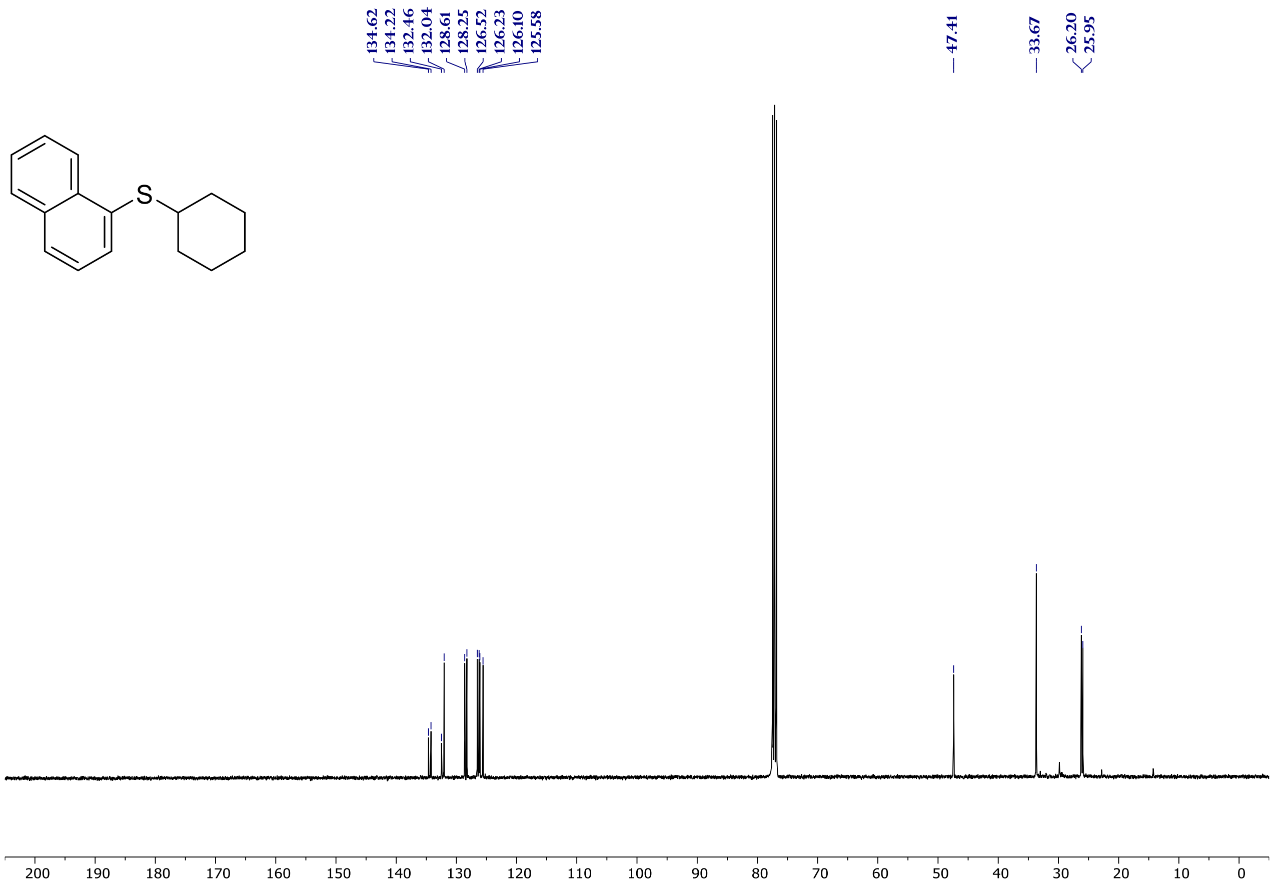

^{13}C-NMR spectrum of cyclohexyl(naphthalen-1-yl) sulfane.

2-(cyclohexylthio) pyridine

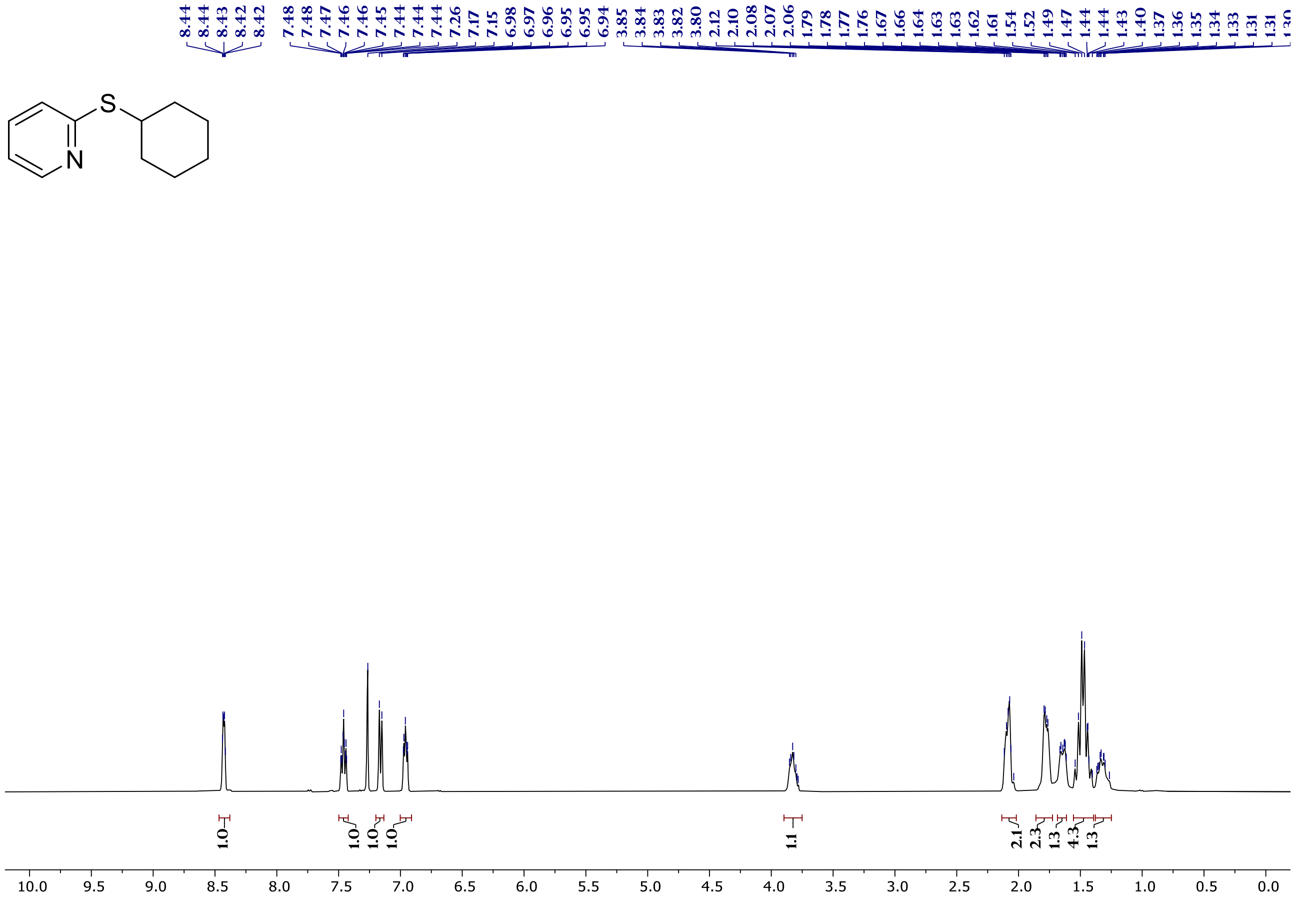

^{1}H-NMR spectrum of 2-(cyclohexylthio) pyridine.

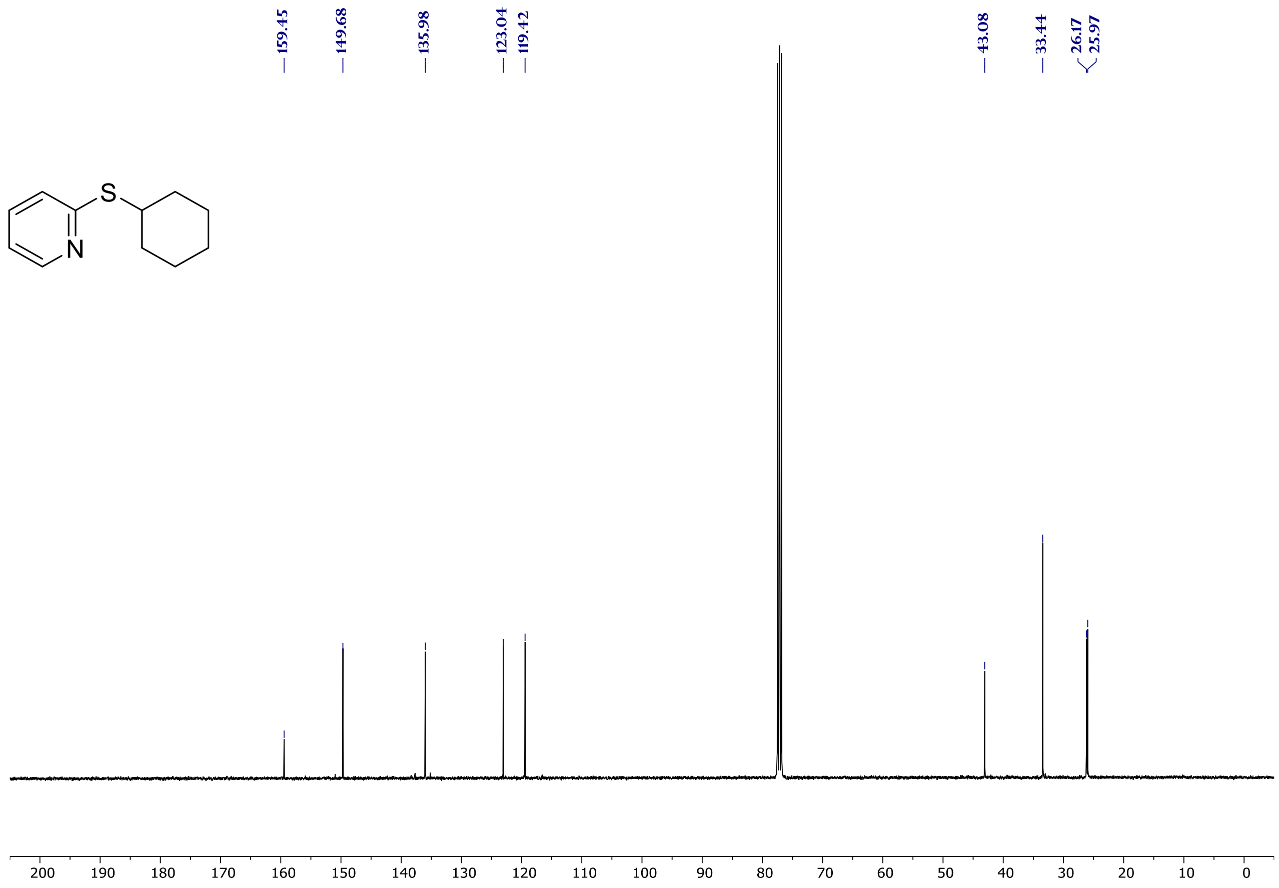

^{13}C-NMR spectrum of 2-(cyclohexylthio) pyridine.

Diphenylsulphane

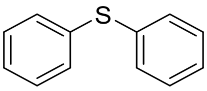

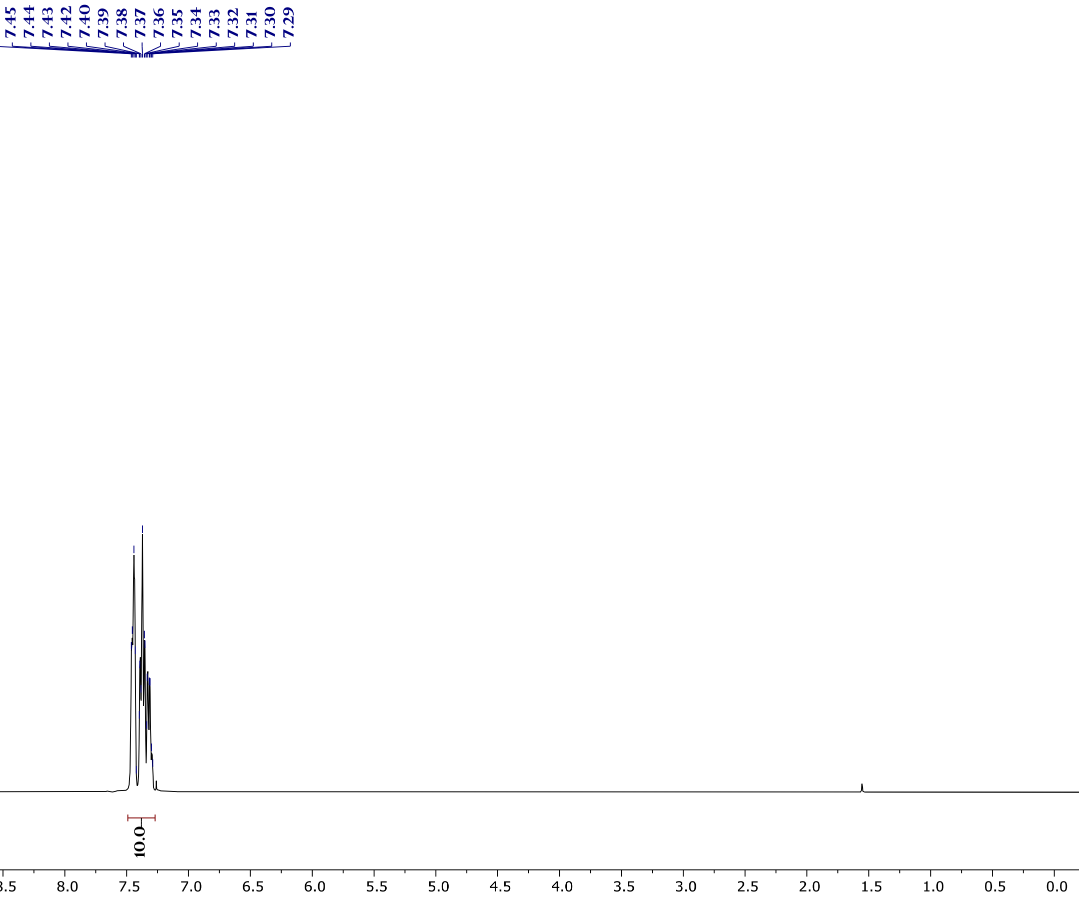

^{1}H-NMR spectrum of diphenylsulphane.

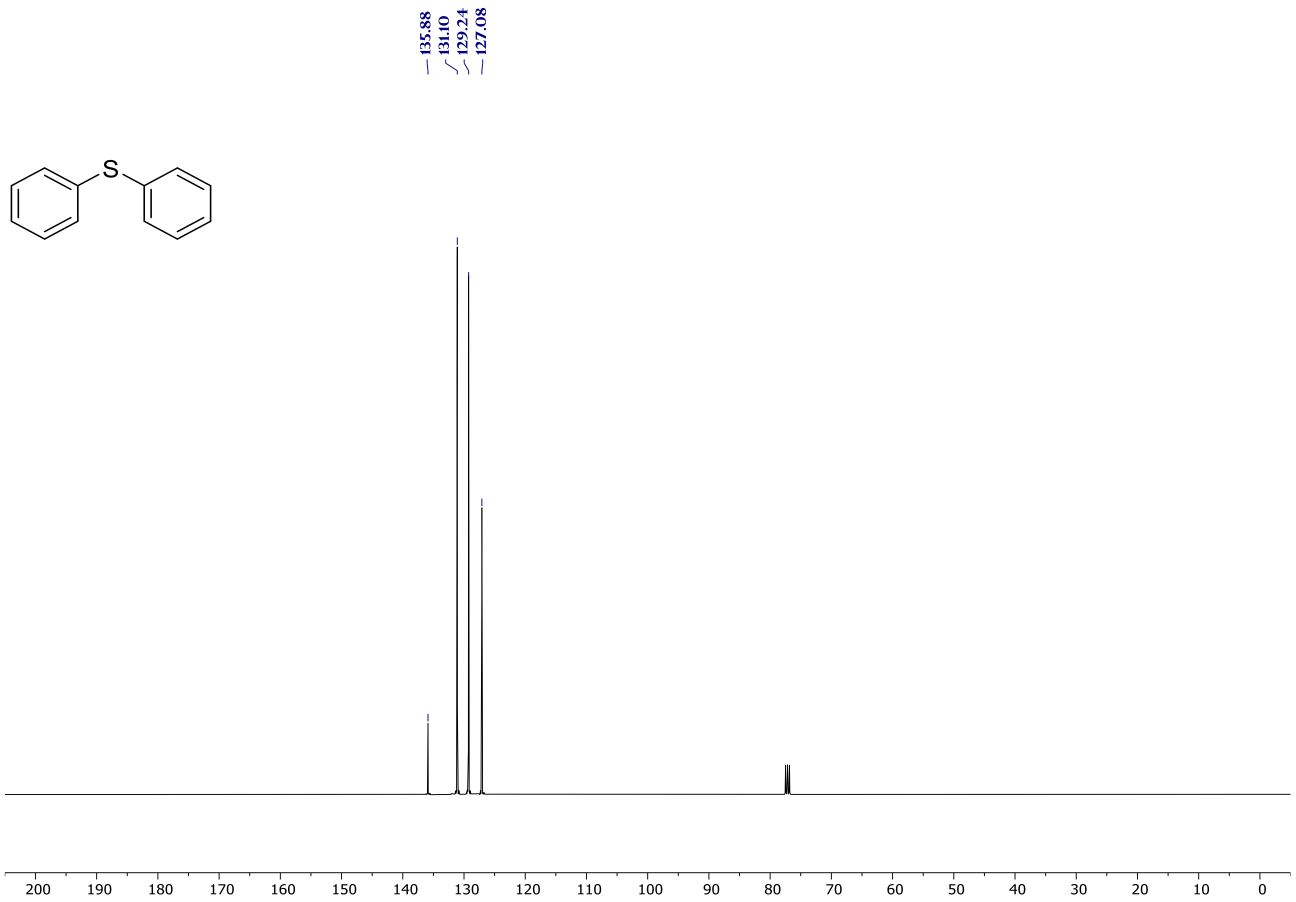

^{13}C-NMR spectrum of diphenylsulphane.

(4-Methoxyphenyl)(phenyl)sulfane

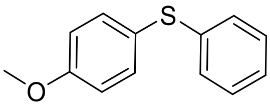

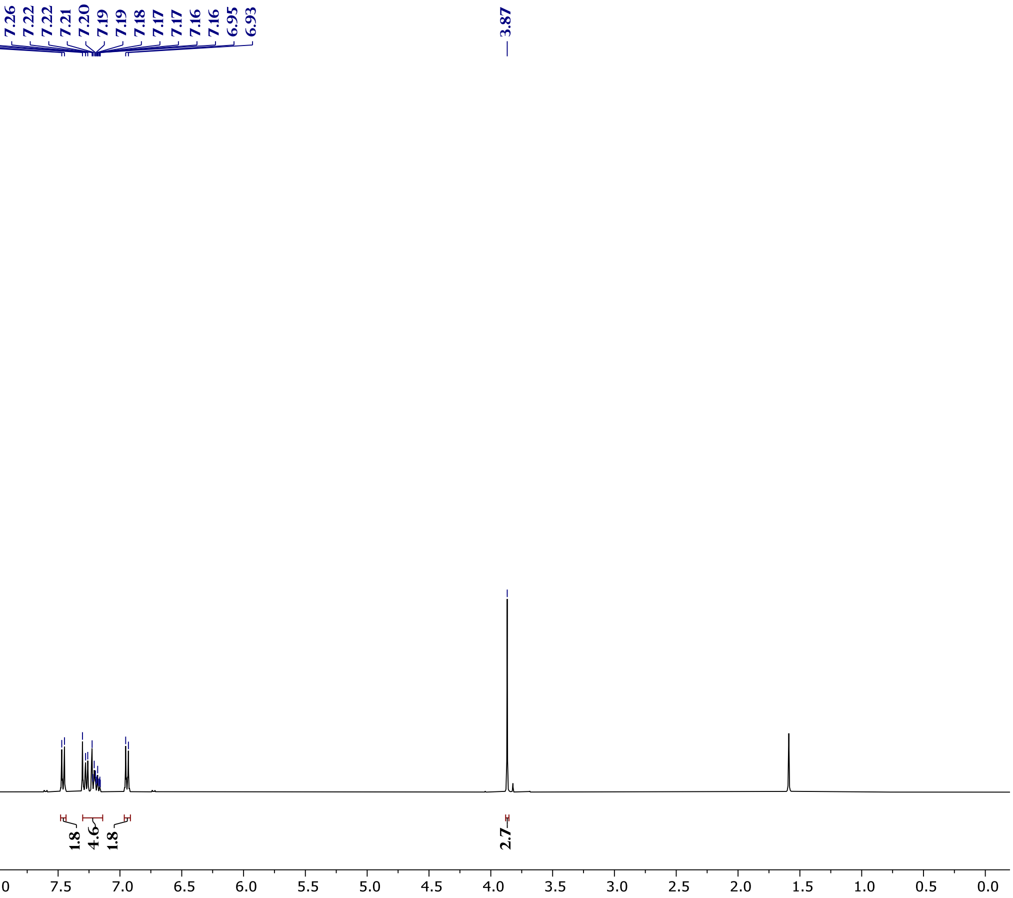

^{1}H-NMR spectrum of (4-methoxyphenyl)(phenyl)sulfane.

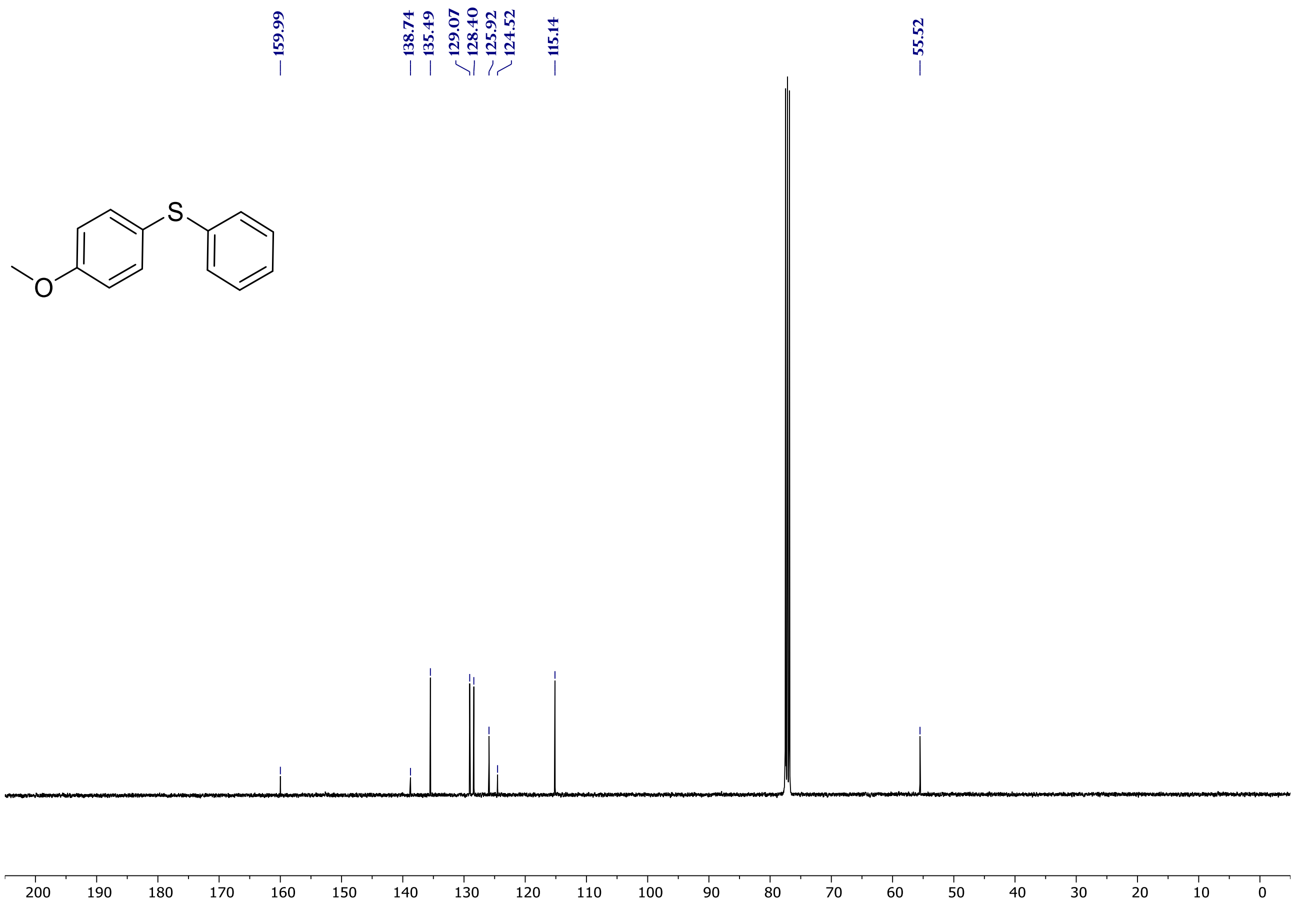

^{13}C-NMR spectrum of (4-methoxyphenyl)(phenyl)sulfane.

Naphthalen-1-yl(phenyl)sulfane

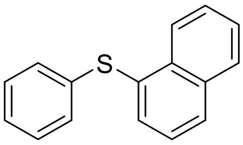

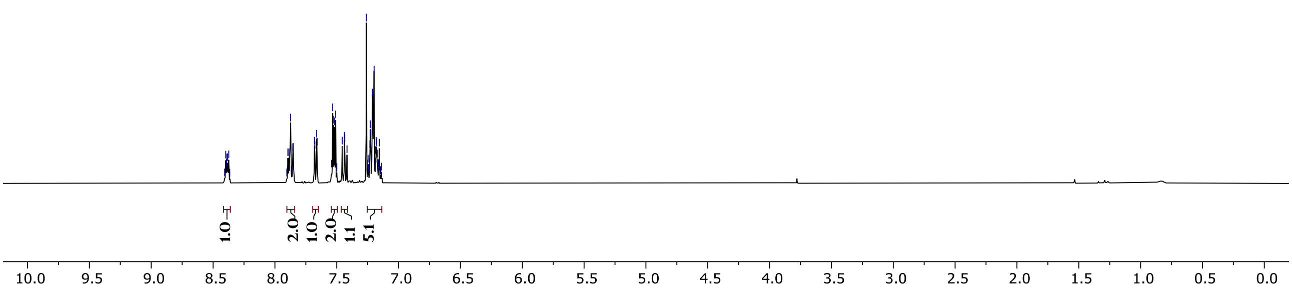

^{1}H-NMR spectrum of naphthalen-1-yl(phenyl)sulfane.

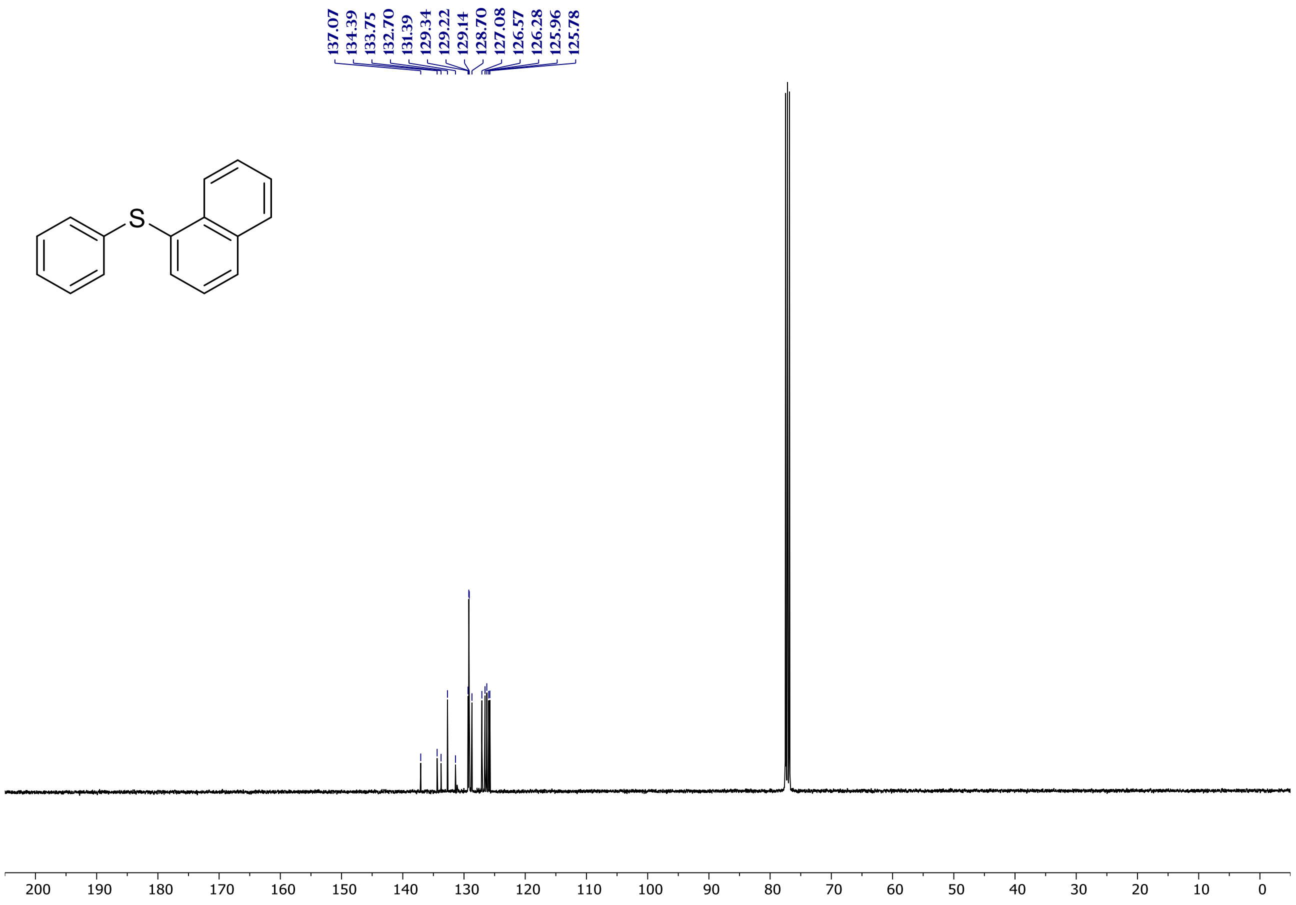

^{13}C-NMR spectrum of naphthalen-1-yl(phenyl)sulfane.

Bis(4-fluorophenyl) sulfide

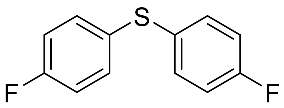

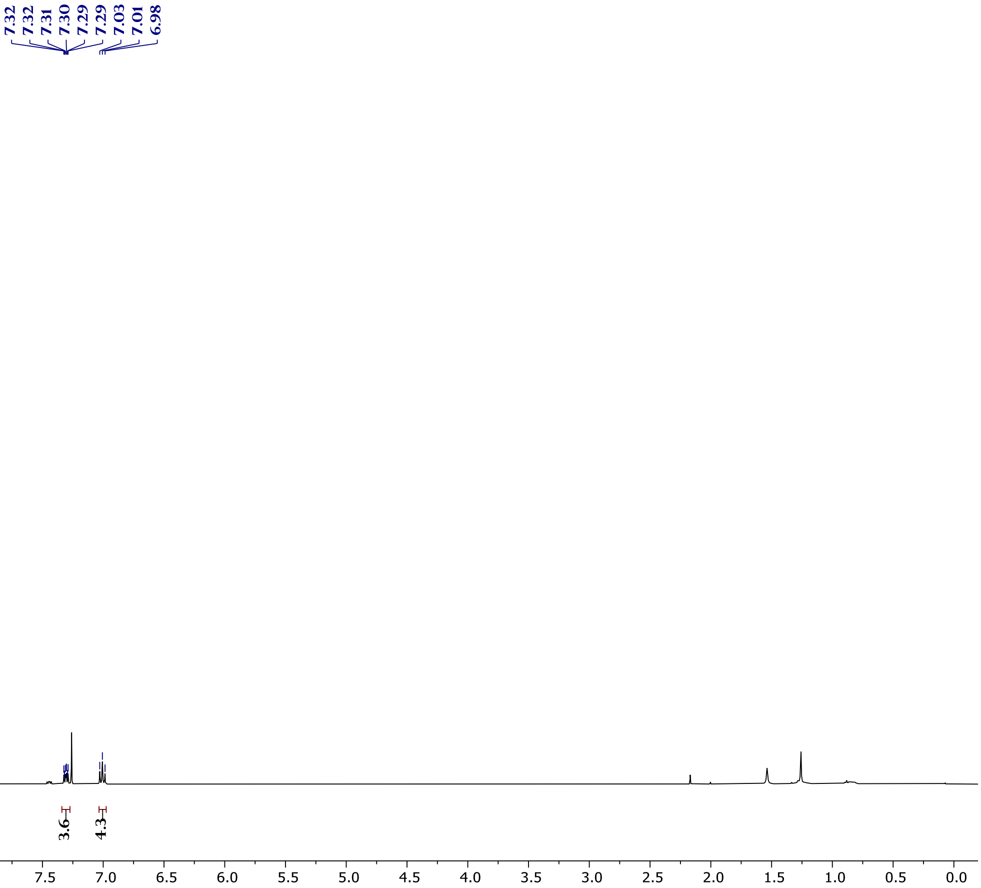

^{1}H-NMR spectrum of bis(4-fluorophenyl) sulfide.

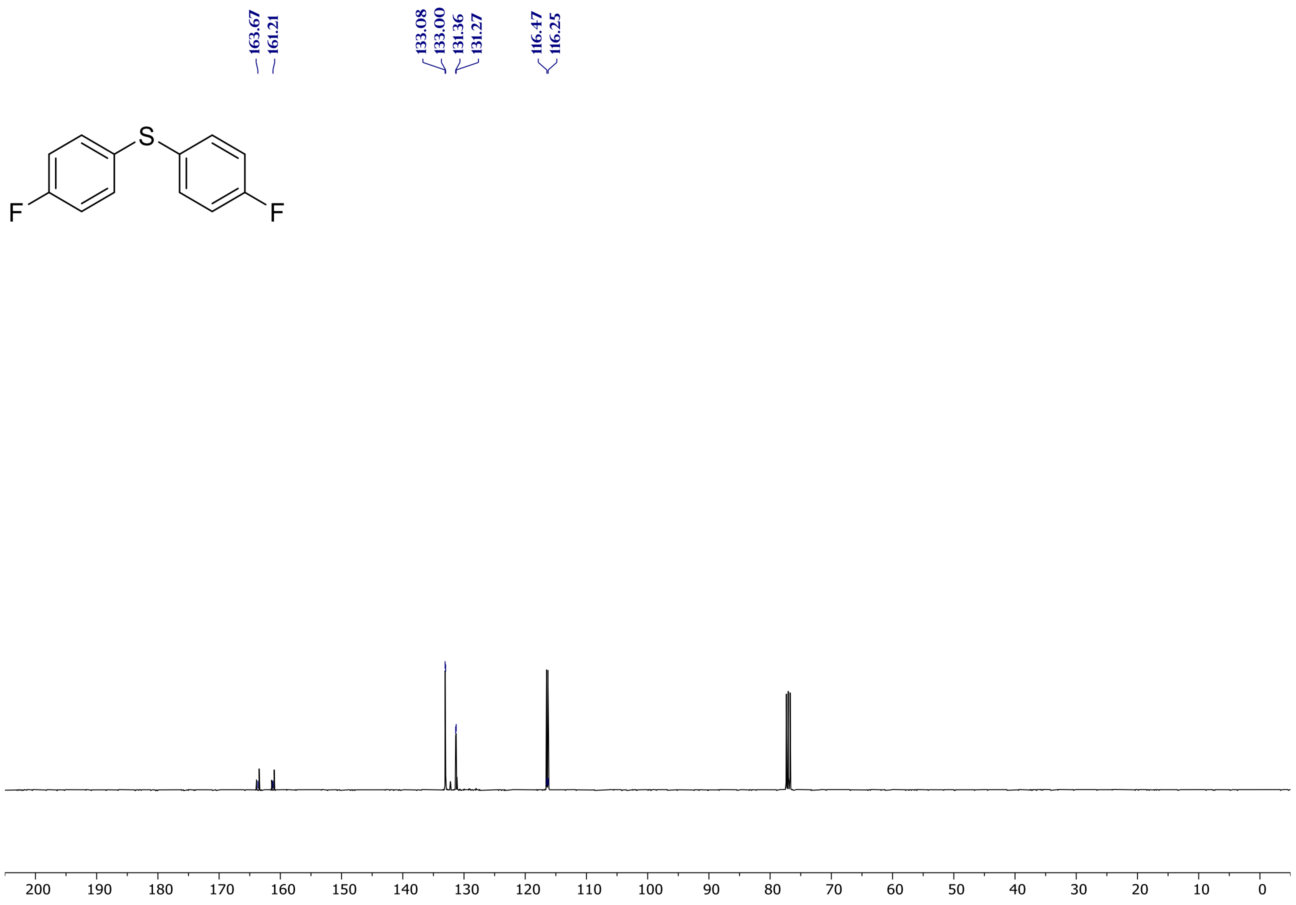

^{13}C-NMR spectrum of bis(4-fluorophenyl) sulfide.

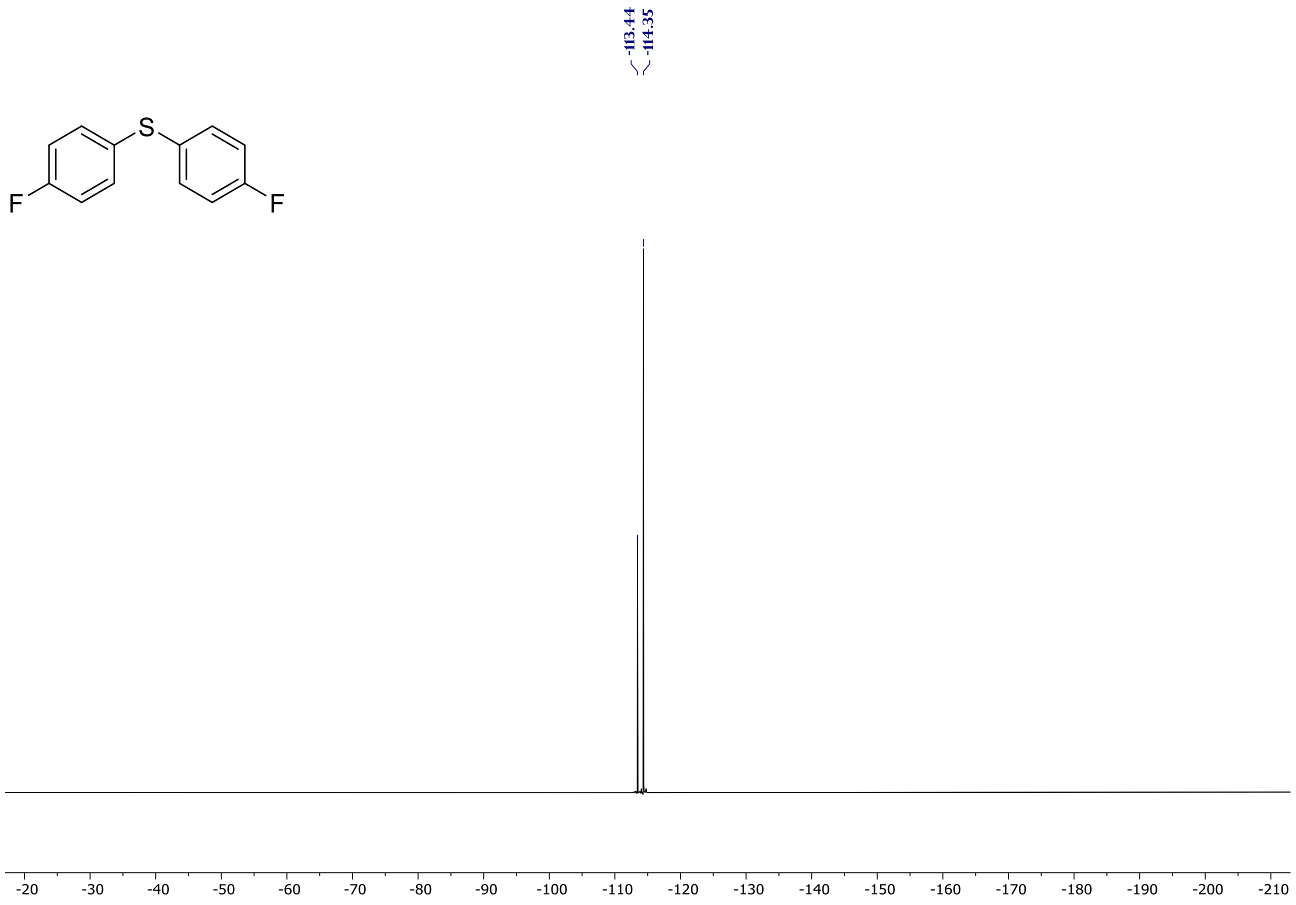

^{19}F-NMR spectrum of bis(4-fluorophenyl) sulfide.

(4-fluorophenyl)(4-methoxyphenyl)sulfane

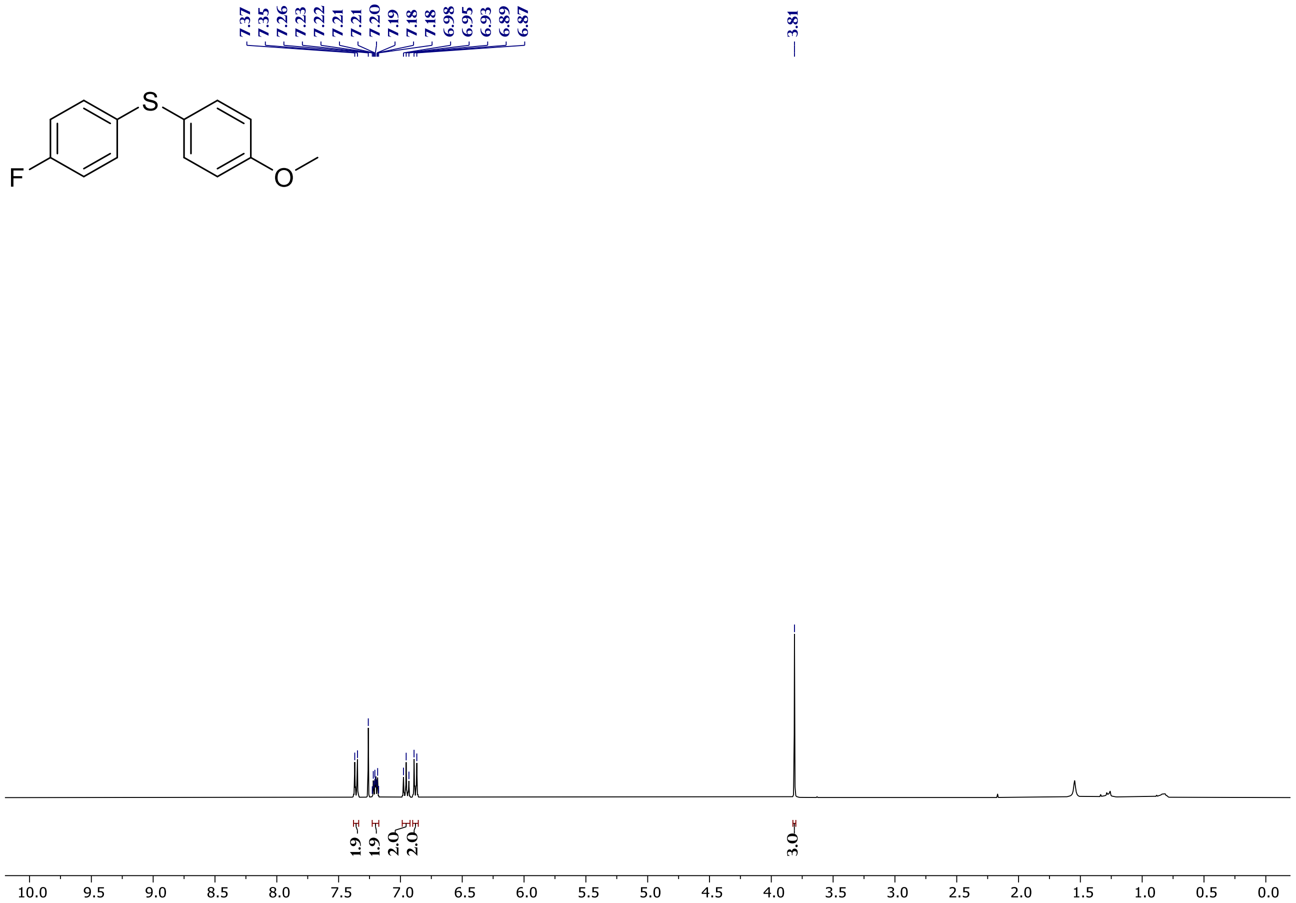

^{1}H-NMR spectrum of (4-fluorophenyl)(4-methoxyphenyl)sulfane.

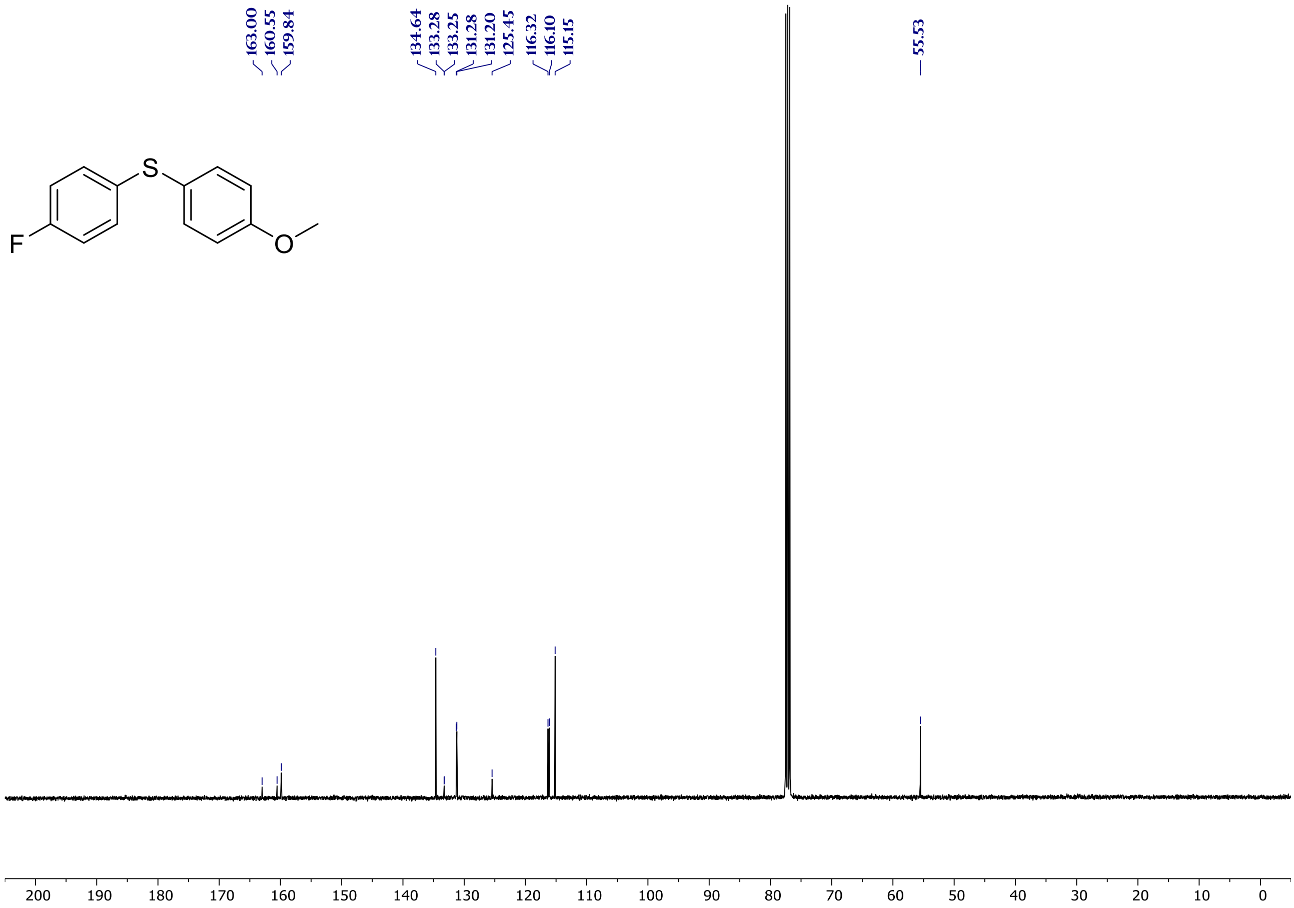

^{13}C-NMR spectrum of (4-fluorophenyl)(4-methoxyphenyl)sulfane.

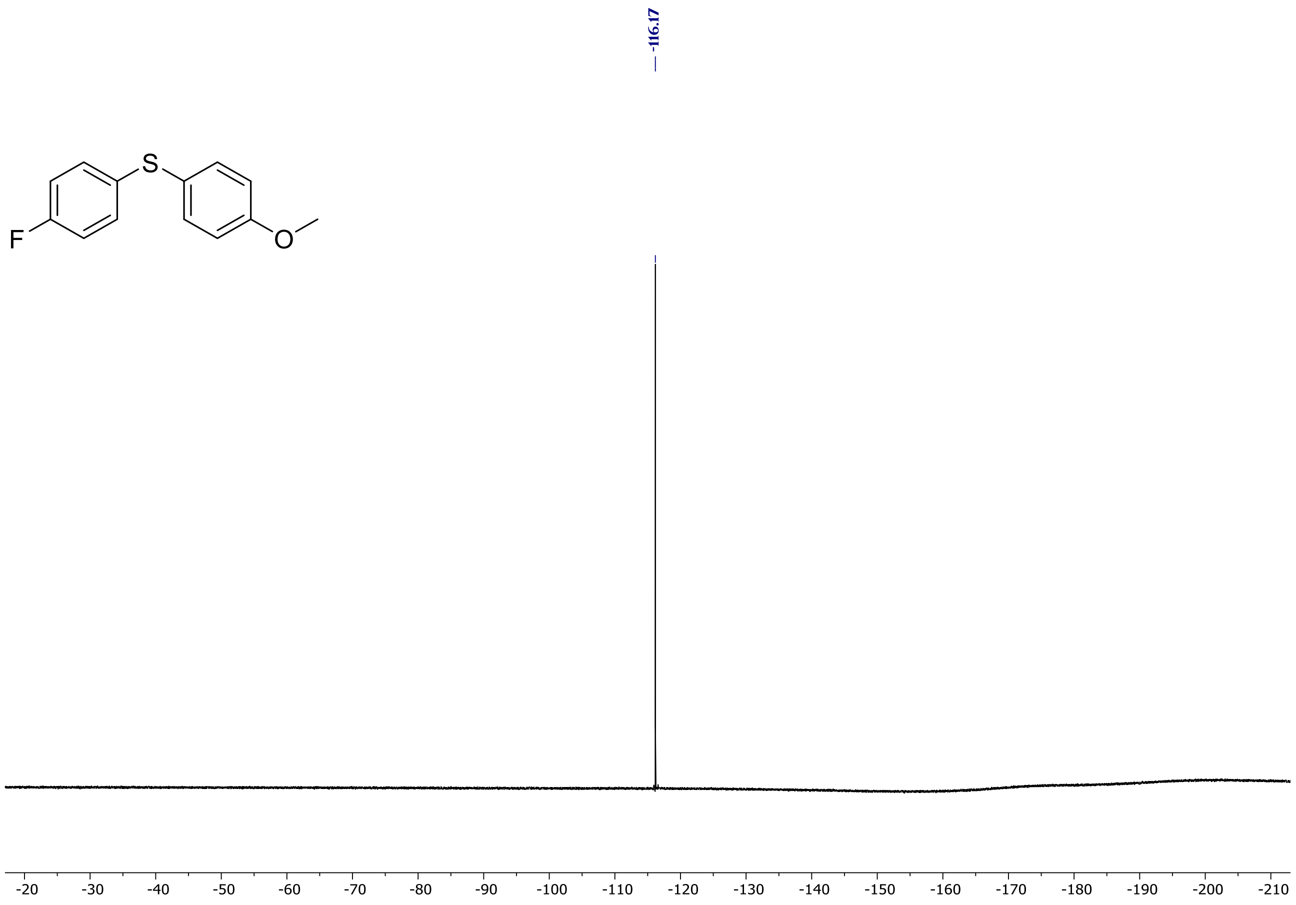

^{19}F-NMR spectrum of (4-fluorophenyl)(4-methoxyphenyl)sulfane.

4-(trifluoromethyl)phenyl phenyl sulfide

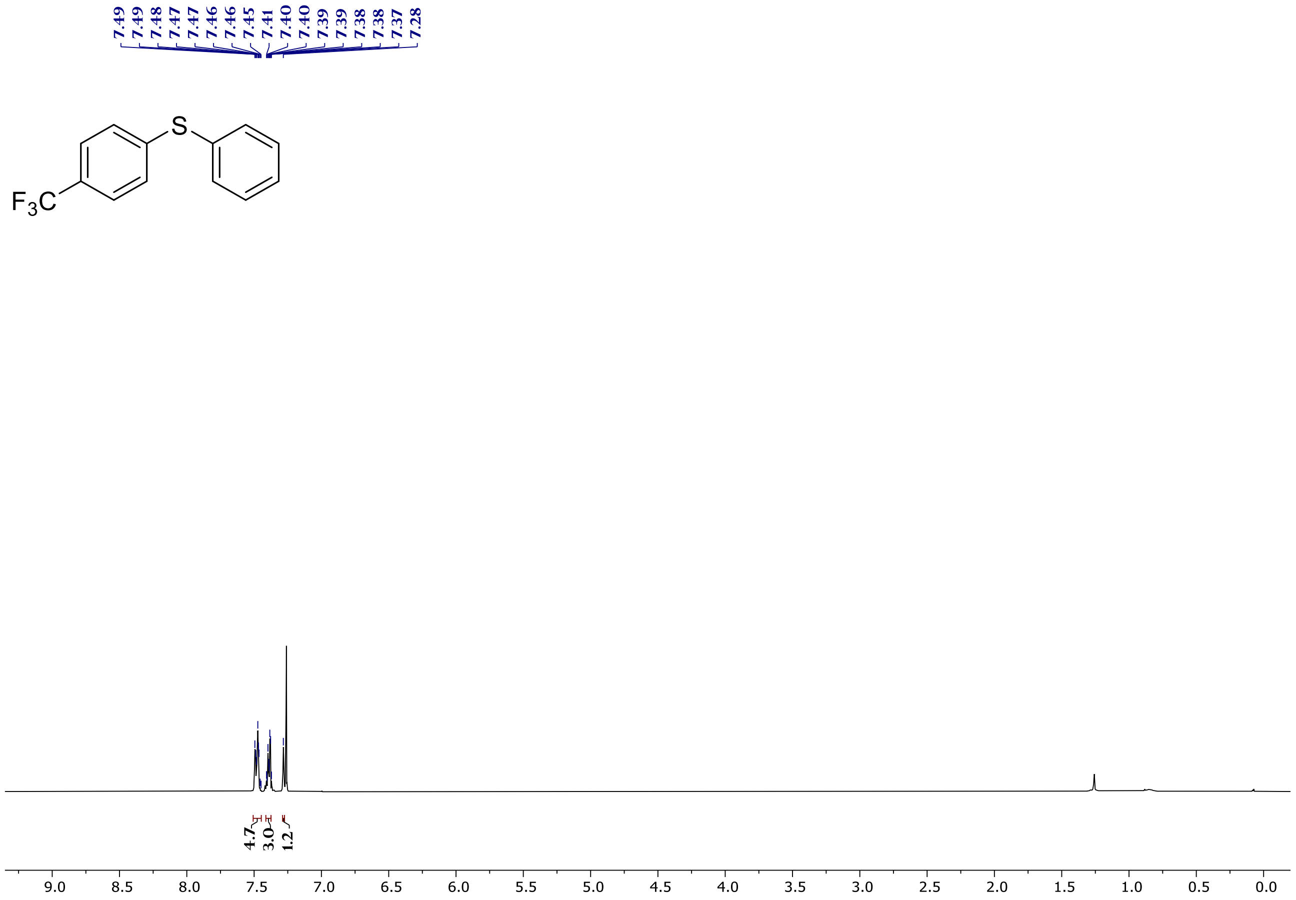

^{1}H-NMR spectrum of 4-(trifluoromethyl)phenyl phenyl sulfide

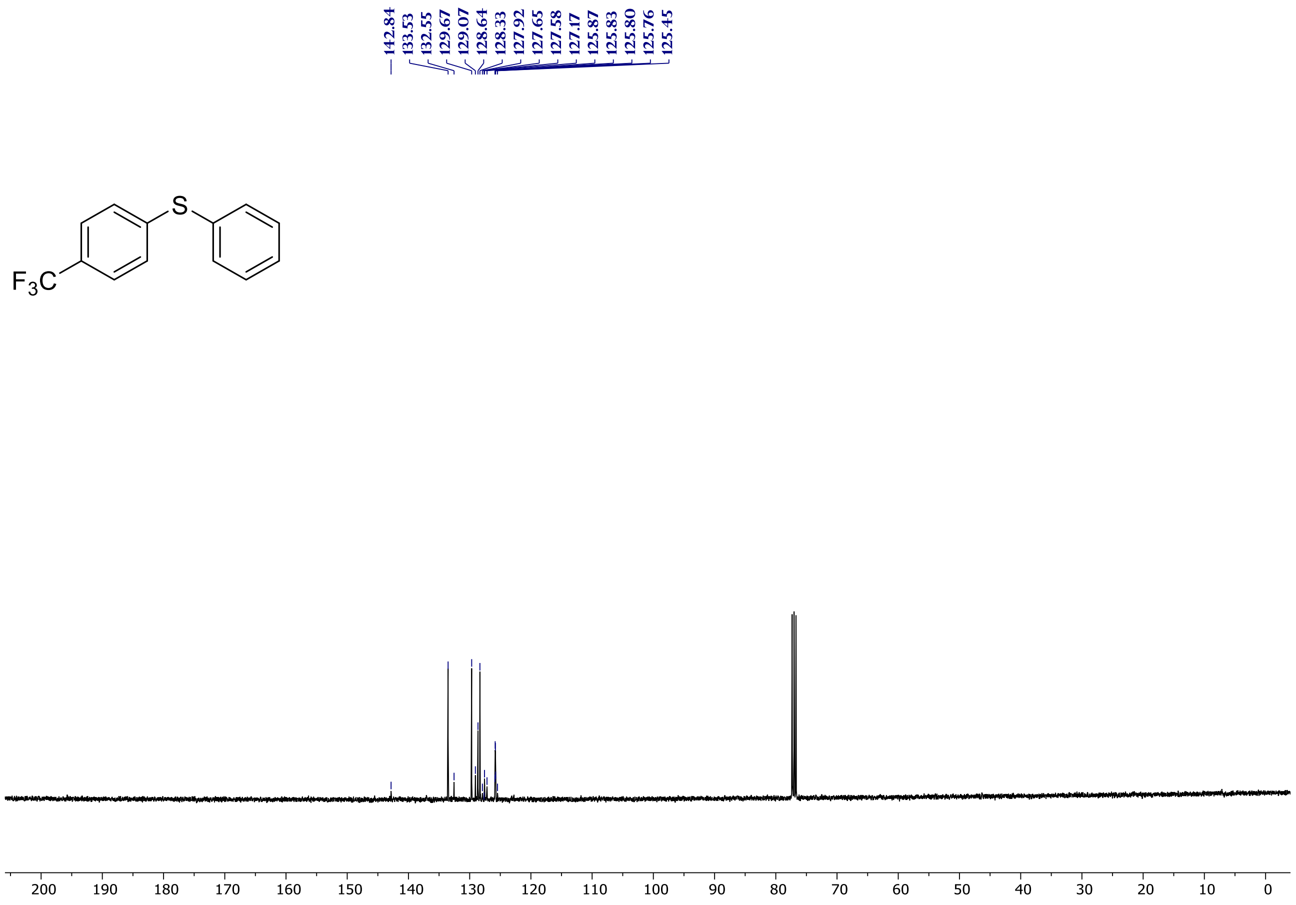

^{13}C-NMR spectrum of 4-(trifluoromethyl)phenyl phenyl sulfide.

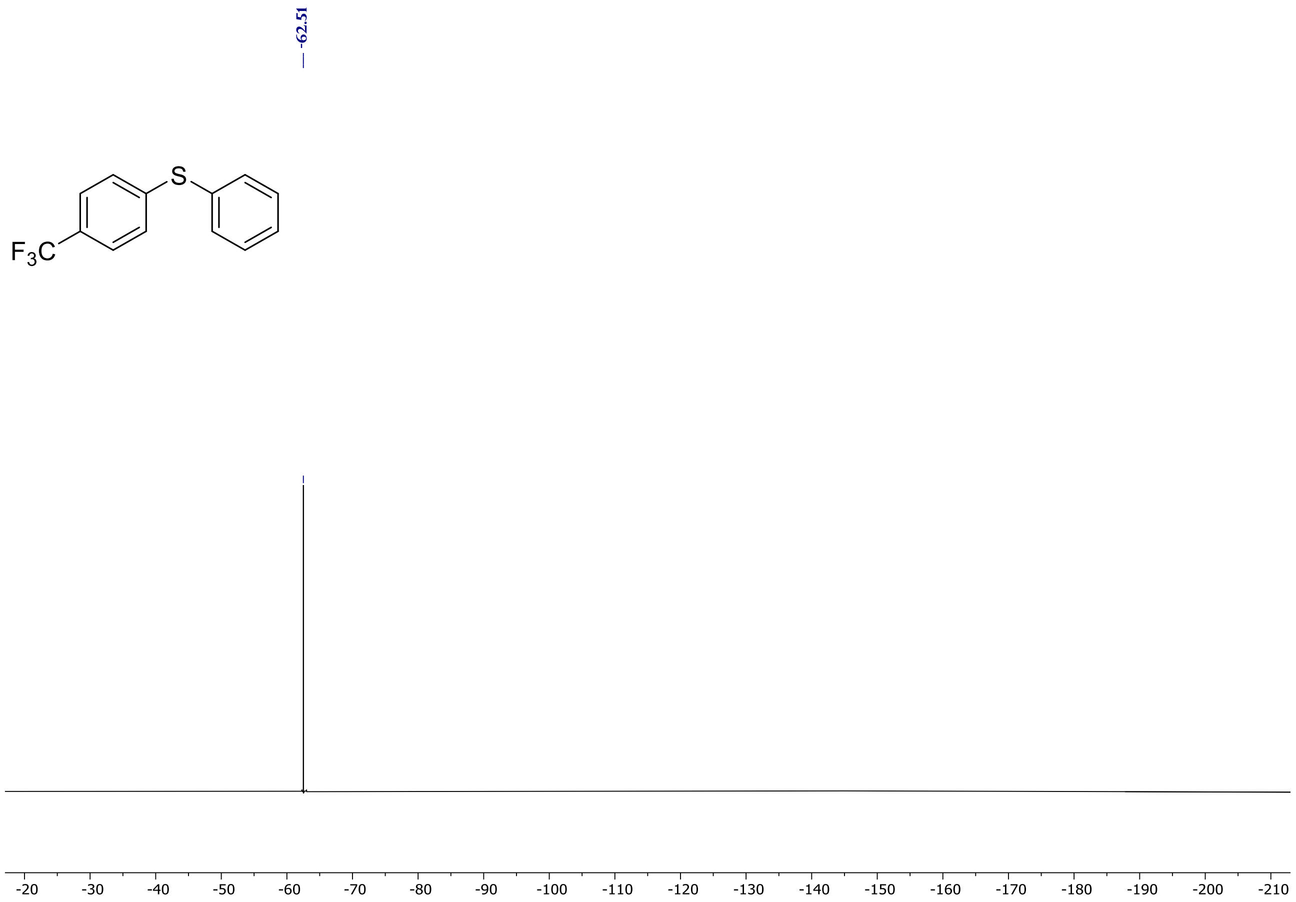

^{19}F-NMR spectrum of 4-(trifluoromethyl)phenyl phenyl sulfide

4-(Phenylthio)benzonitrile

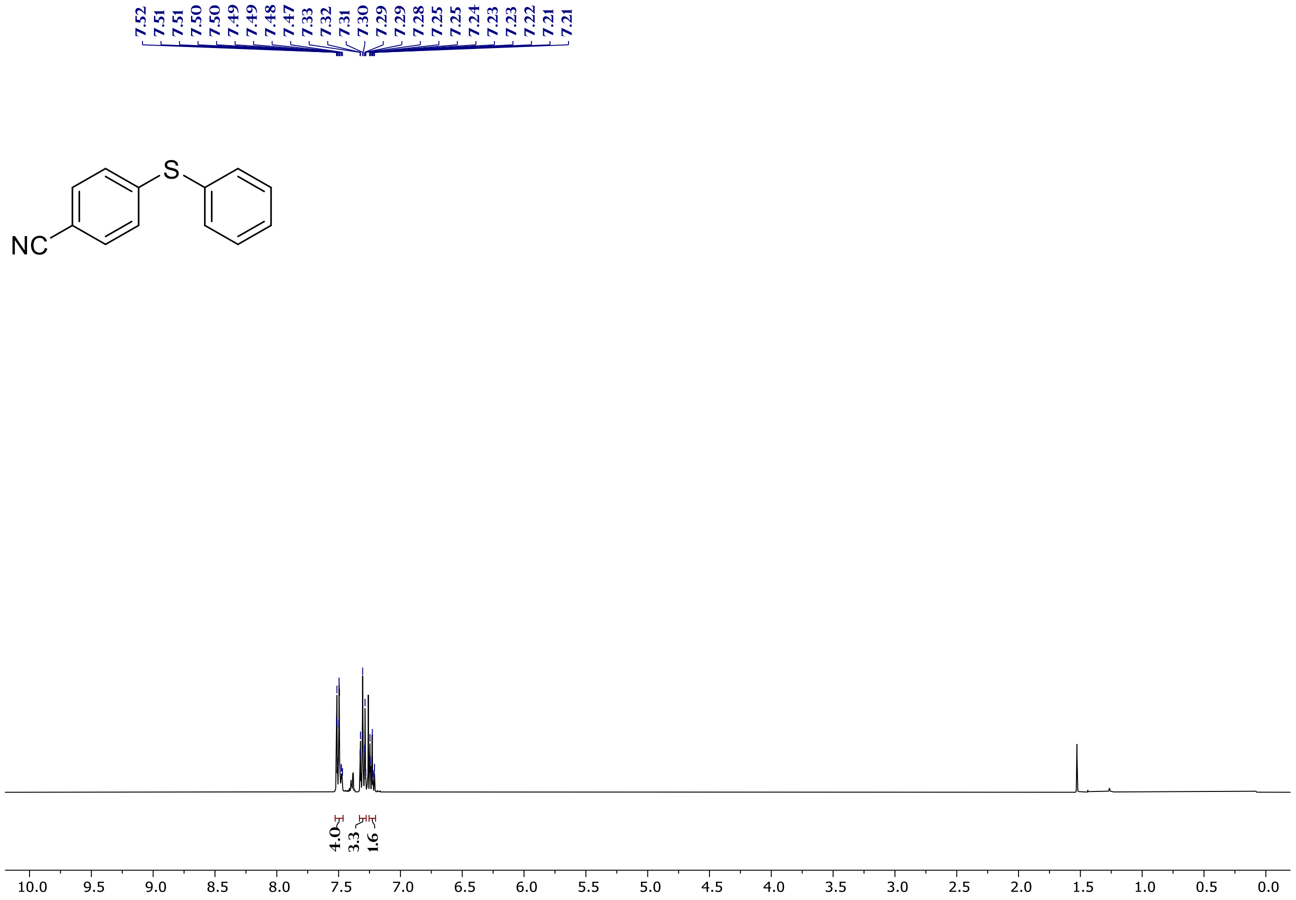

^{1}H-NMR spectrum of 4-(Phenylthio)benzonitrile.

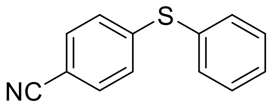

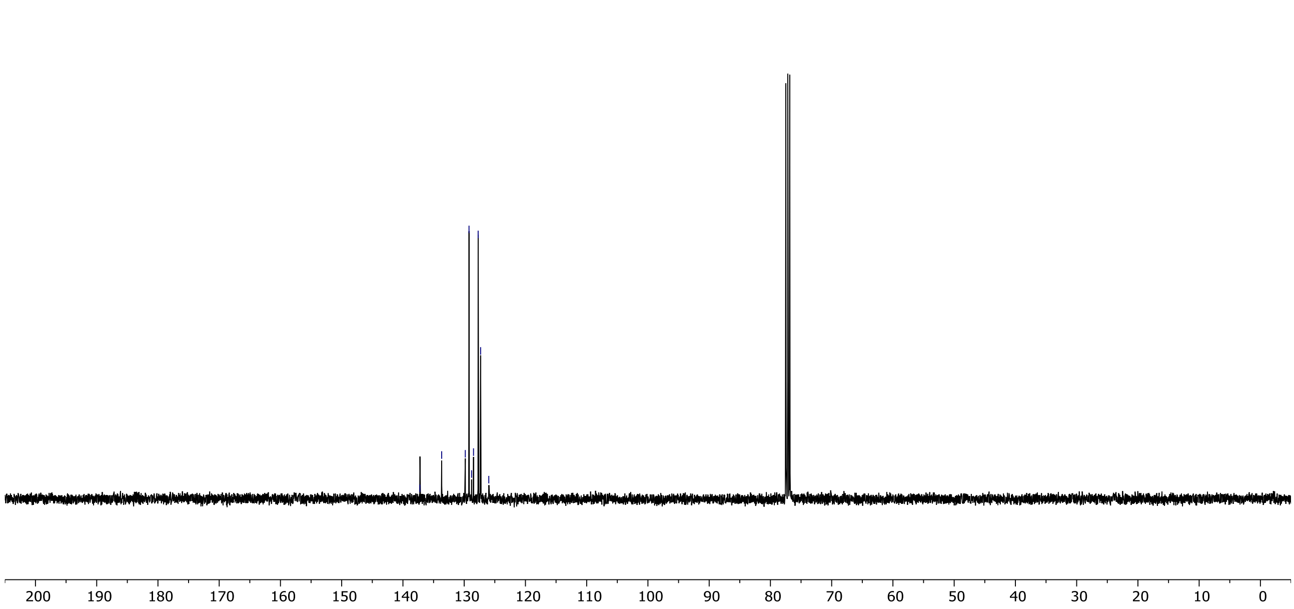

^{13}C-NMR spectrum of 4-(Phenylthio)benzonitrile.

(2-bromophenyl)(2,4-dimethylphenyl)sulfane

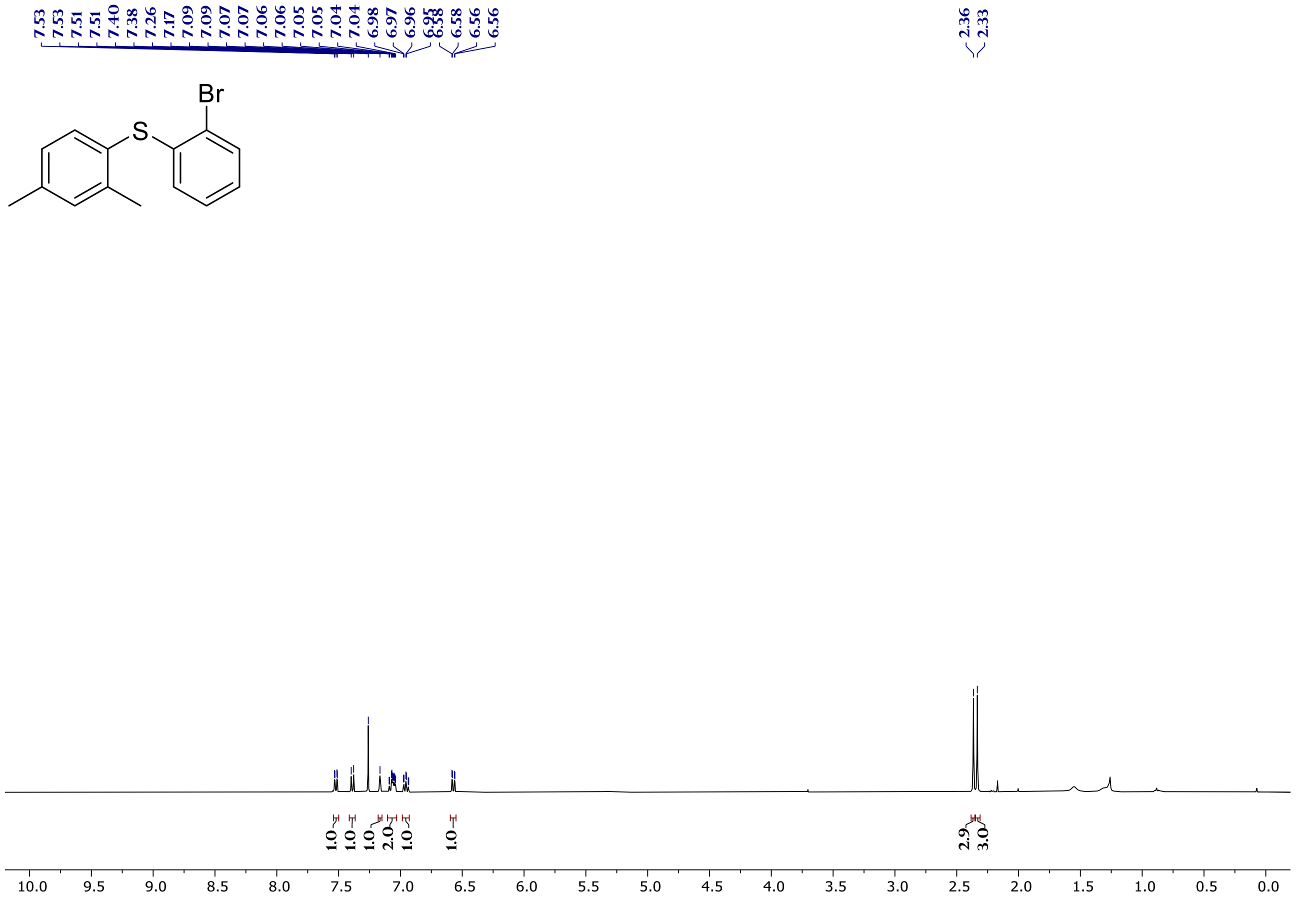

^{1}H-NMR spectrum of (2-bromophenyl)(2,4-dimethylphenyl)sulfane.

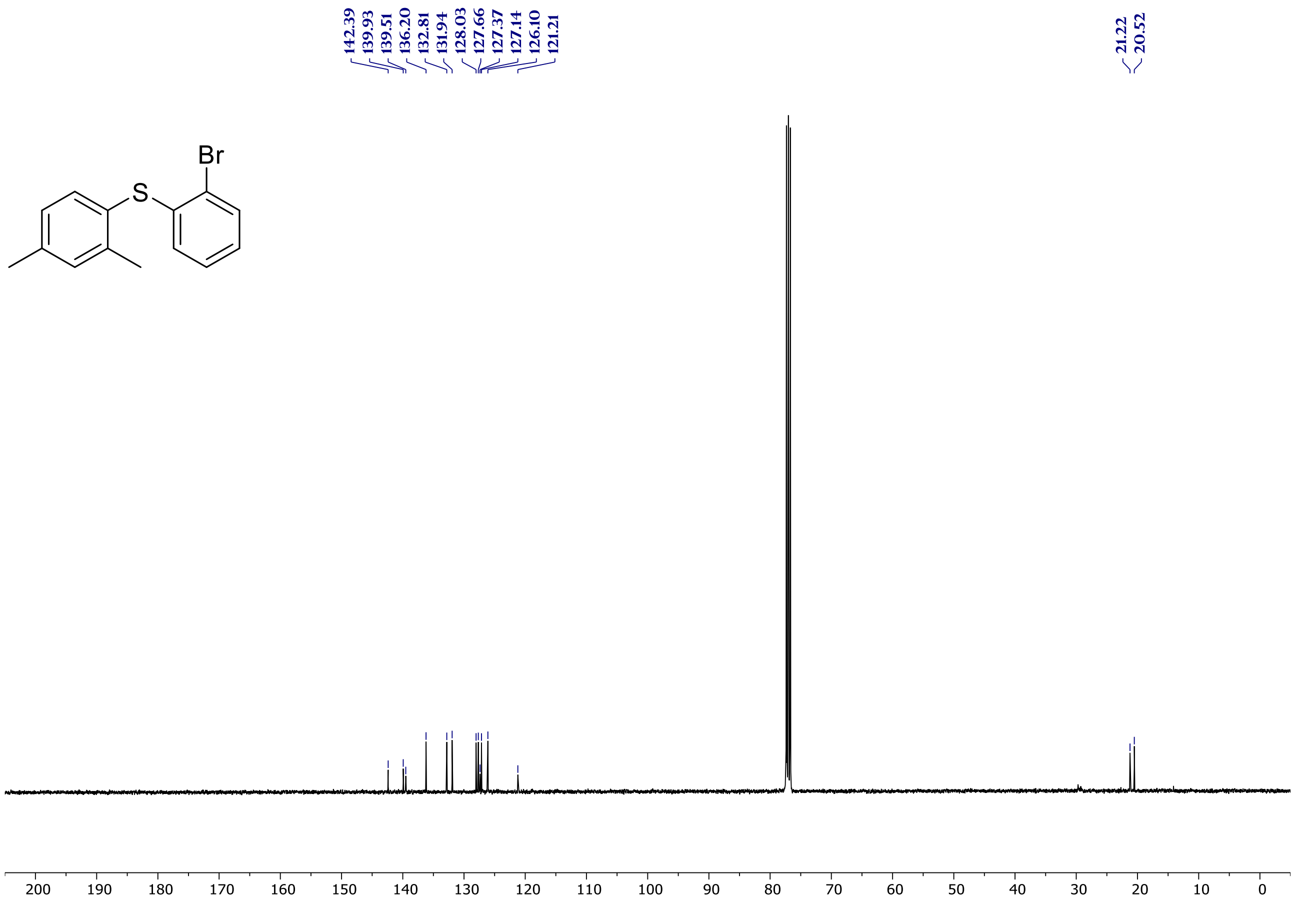

^{13}C-NMR spectrum of (2-bromophenyl)(2,4-dimethylphenyl)sulfane.

o-(but-3-enyloxy)phenyl phenyl sulfide

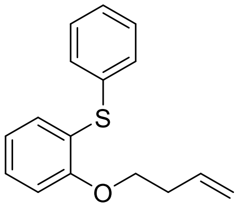

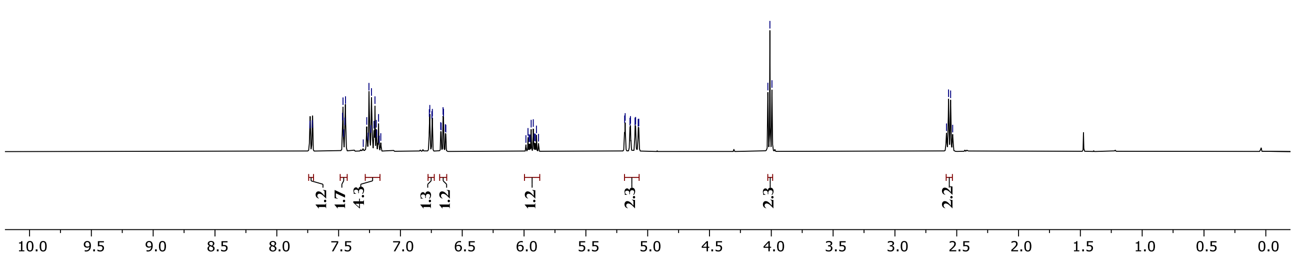

^{1}H-NMR spectrum of o-(but-3-enyloxy)phenyl phenyl sulfide.

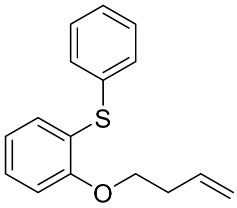

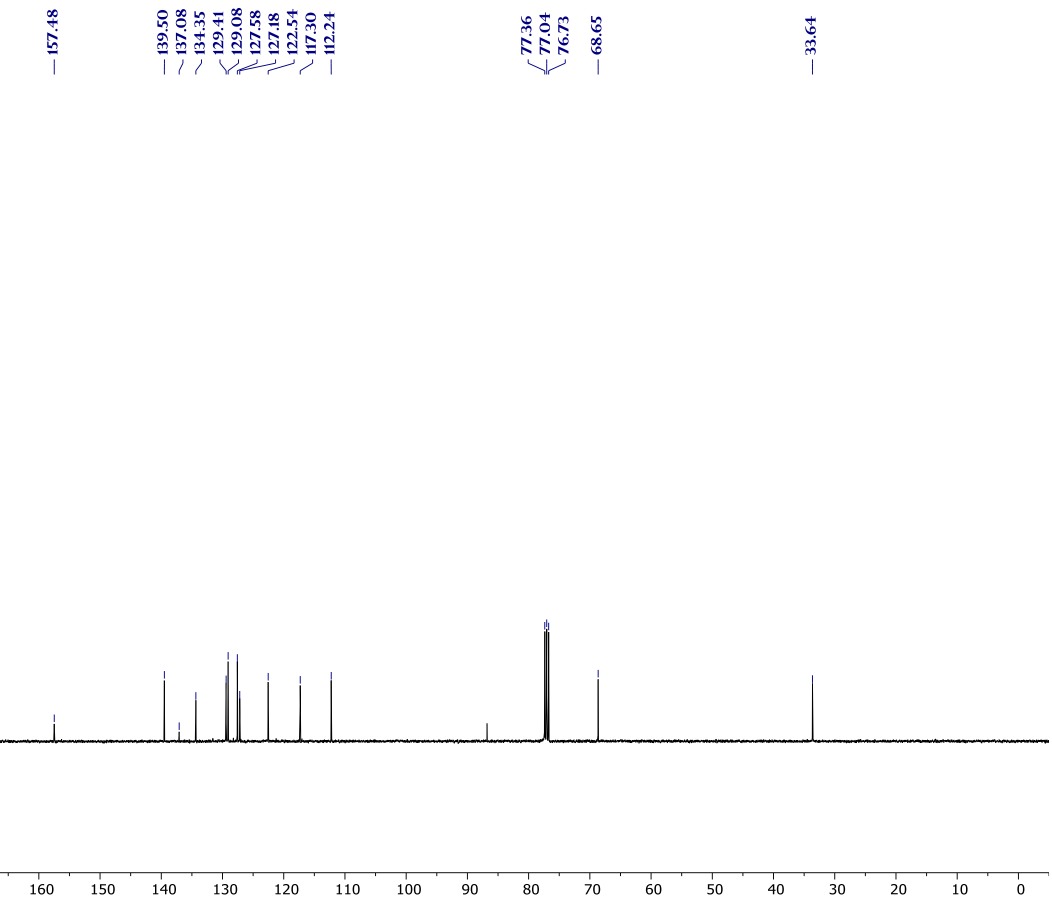

^{13}C-NMR spectrum of o-(but-3-enyloxy)phenyl phenyl sulfide.

References of the Supporting Information.

(1) Ishikawa, H.; Yamaguchi, S.; Nakata, A.; Nakajima, K.; Yamazoe, S.; Yamasaki, J.; Mizugaki, T.; Mitsudome, T. Phosphorus-Alloying as a Powerful Method for Designing Highly Active and Durable Metal Nanoparticle Catalysts for the Deoxygenation of Sulfoxides: Ligand and Ensemble Effects of Phosphorus. *JACS Au.* **2022**, *2*, 419–427.

(2) Okauchi, T.; Kuramoto, K.; Kitamura, M. Facile Preparation of Aryl Sulfides Using Palladium Catalysis under Mild Conditions. *Synlett.* **2010**, *2010*, 2891–2894.

(3) Cheng, J. H.; Ramesh, C.; Kao, H. L.; Wang, Y. J.; Chan, C. C.; Lee, C. F. Synthesis of Aryl Thioethers through the N -Chlorosuccinimide-Promoted Cross-Coupling Reaction of Thiols with Grignard Reagents. *J. Org. Chem.* **2012**, *77*, 10369–10374.

(4) Li, P.; Tu, J. L.; Hu, A. M.; Guo, L.; Yang, C.; Xia, W. Photoinduced Decatungstate-Catalyzed C(Sp3)–H Thioetherification by Sulfinate Salts. *Org. Biomol. Chem.* **2024**, *22*, 3420–3424.

(5) Bao, Y.; Yang, X.; Dai, Z.; Ji, S.; Zhou, Q.; Yang, F. Iodine-Promoted Tunable Synthesis of 2-Naphthyl Thioethers and 1-Naphthyl Thioethers. *Adv. Synth. Catal.* **2019**, *361*, 2154–2158.

(6) Martín, M. T.; Marín, M.; Maya, C.; Prieto, A.; Nicasio, M. C. Ni(II) Precatalysts Enable Thioetherification of (Hetero)Aryl Halides and Tosylates and Tandem C−S/C−N Couplings. *Chem. Eur.J.* **2021**, *27*, 12320–12326.

(7) Cao, H.; Shi, Y.; Ma, J.; Yan, P.; Cong, X.; Bie, F. Palladium- and Nickel-Catalyzed Synthesis of Thioethers via Thioesters – Aryl Halides Coupling. *Tetrahedron Lett.* **2023**, *119*, 154414.

(8) Li, J. X.; Tian, R.; Zhu, Y. M. Nickel-Catalyzed Decarbonylation of Aryl Anhydrides for the Synthesis of Thioesters and Thioethers: CS2/DBU as a Surrogate Sulfur Source. *Tetrahedron.* **2024**, *152*, 133814.

(9) Li, B.; Wang, J.; Tu, G.; Ju, G.; Zhou, K.; Zhao, Y. Hemin-Promoted Direct C−H Thiolation in the Synthesis of Diaryl Sulfides. *Eur. J. Org. Chem.* **2023**, *26*, e202201458.

(10) Lui, C.; Szostak, M. Decarbonylative Thioetherification by Nickel Catalysis Using Air- and Moisture-Stable Nickel Precatalysts. *Chem.Commun.* **2018**, *54*, 2130–2133.

(11) Li, Y.; Liu, L.; Shan, D.; Liang, F.; Wang, S.; Yu, L.; Liu, J., Wang, Q., Shao, X., Zhu, D. Exploration on Metal-Catalytic Conversion of Sulfinyl Sulfones. *ACS Catal.* **2023**, *13*, 13474–13483.

(12) Pramanik, M.; Das, S.; Babaahmadi, R.; Pahar, S.; Wirth, T.; Richards, E.; Melen, R. L. B(C6F5)3-Catalyzed Selective C–H Chalcogenation of Arenes and Heteroarenes. *Chem.* **2024**, *10*, 2901–2915.

(13) Abeywickrema, A. N.; Beckwith, A. L. J. Mechanistic and Kinetic Studies of the Thiodediazoniation Reaction. *J. Am. Chem. Soc.* **1986**, *108*, 8227–8229.
